# Effectiveness and safety of chronic diuretic use in older adults: an umbrella review of recently published systematic reviews and meta-analyses of randomized-controlled trials

**DOI:** 10.1007/s41999-025-01229-5

**Published:** 2025-05-25

**Authors:** Eveline van Poelgeest, Konstantinos Prokopidis, Tuğba Erdogan, Min Ji Kwak, Karolina Piotrowicz, Luca Paoletti, Annette Eidam, Fatma Özge Kayhan Koçak, Birkan Ilhan, Alessia Beccacece, George Soulis, Serdar Özkök, Gulistan Bahat, Eva Topinková, Joost Daams, M. Louis Handoko, Parag Goyal, Jerzy Gąsowski, Antonio Cherubini, Nicola Veronese, Giuseppe Dario Testa, Wade Thompson, Nathalie van der Velde, European Geriatric Medicine Society Special Interest Groups of i. Pharmacology; ii. Cardiovascular Disease and iii. Systematic Review and Meta-analysis

**Affiliations:** 1 Section of Geriatrics, Department of Internal Medicine, Amsterdam University Medical Center, Location University of Amsterdam, Meibergdreef 9, Amsterdam, The Netherlands; 2 Amsterdam Public Health Research Institute, Aging and Later Life, Amsterdam, The Netherlands; 3 Department of Musculoskeletal and Ageing Science, Institute of Life Course and Medical Sciences, University of Liverpool, Liverpool, UK; 4 Division of Geriatrics, Department of Internal Medicine, Istanbul University, Istanbul Medical School, Istanbul, Turkey; 5 Division of Geriatric and Palliative Medicine, McGovern Medical School, University of Texas Health Science Center at Houston, Houston, TX USA; 6 Department of Internal Medicine and Gerontology, Jagiellonian University Medical College, Kraków, Poland; 7 Pharmacy Department, IRCCS San Raffaele Hospital, Milan, Italy; 8 Geriatric Center, Medical Faculty Heidelberg, Heidelberg University, Heidelberg, Germany; 9 Division of Geriatrics, Department of Internal Medicine, Health Sciences University Tepecik Training and Research Hospital, İzmir, Turkey; 10 Division of Geriatrics, Department of Internal Medicine, Liv Hospital Vadistanbul, Istanbul, Turkey; 11 Geriatria, Accettazione geriatrica e Centro di ricerca per l’invecchiamento, IRCCS INRCA, Ancona, Italy; 12 Hellenic Society for the Study and Research of Ageing, Athens, Greece; 13 Division of Geriatrics, Department of Internal Medicine, Istanbul Medical Faculty, Istanbul University, Istanbul, Turkey; 14 Department of Geriatrics and Internal Medicine, 1st Faculty of Medicine Charles University, Prague, Czech Republic; 15 General Faculty Hospital in Prague, Faculty of Health and Social Sciences, University of South Bohemia, Ceske Budejovice, Czech Republic; 16 Medical Library, Amsterdam University Medical Center, University of Amsterdam, Amsterdam, The Netherlands; 17 Department of Cardiology, University Medical Center Utrecht/Transplantation Center UMC Utrecht, Utrecht, The Netherlands; 18 Amsterdam University Medical Center, Amsterdam, The Netherlands; 19 Program for the Care and Study of the Aging Heart, Department of Medicine, Weill Cornell Medicine, New York, NY USA; 20 Department of Clinical and Molecular Sciences, Università Politecnica delle Marche, Ancona, Italy; 21 Saint Camillus International University of Health Sciences, Rome, Italy; 22 Department of Geriatric and Intensive Care Medicine, Careggi Hospital, University of Florence, Florence, Italy; 23 Department of Anesthesiology, Pharmacology, and Therapeutics, Faculty of Medicine, University of British Columbia, Vancouver, Canada

**Keywords:** Adverse outcome, Benefit, Efficacy, Meta-analysis, Diuretics, Umbrella review

## Abstract

**Background:**

Healthcare providers should balance the potential risks and benefits of chronic diuretic use, particularly in older adults, as with age, diuretic benefits may decline and risks increase. A comprehensive synthesis and critical evaluation of the available evidence on chronic diuretic treatment effects is currently lacking.

**Methods:**

We conducted an umbrella review of systematic reviews and meta-analyses published since 2018 on health outcomes associated with diuretic use in randomized-controlled trials (RCTs). We conducted random-effects meta-analysis for pooled effect estimates and narratively summarized data that could not be pooled.

**Results:**

We included 741 effect estimations from 117 systematic reviews (SRs) on 1566 RCTs in individuals aged 62 ± 6 years. Of our 33 meta-analyses, 11 provided convincing, high-quality evidence: finerenone reduced the risk of cardiovascular (CV) mortality and end-stage kidney disease in individuals with chronic kidney disease (CKD) and/or type 2 diabetes (T2D). Torasemide reduced the risk of heart failure-related hospitalization (HFH) more than furosemide in individuals with HF. Thiazides reduced CV events in individuals with hypertension. Mineralocorticoid receptor antagonists (MRAs) reduced HFH, but also increased hyperkalemia risk in individuals with HF. MRAs also reduced the risk of atrial fibrillation in those with HF or CVD, and reduced HFH, major adverse cardiovascular events (MACEs), > 40% eGFR decrease, and composite kidney outcomes in individuals with CKD and/or T2D. Lower quality evidence suggests that in older (≥ 65 years), but not in younger adults, diuretics may reduce CV mortality, but also increase adverse event (AE) risk.

**Conclusions:**

Our umbrella review offers a comprehensive and up-to-date evaluation of the benefits and harms of diuretics. However, further research is needed to establish their efficacy and safety in populations commonly seen in clinical practice, especially older adults living with multimorbidity and frailty.

**Graphical abstract:**

**Supplementary Information:**

The online version contains supplementary material available at 10.1007/s41999-025-01229-5.

## Key summary points

**Aim:**

This umbrella review aimed to summarize the literature on the efficacy and safety of chronic diuretic treatment in adults.

**Findings:**

Certain diuretics or diuretic subclasses offer significant benefits for key clinical outcomes, such as reducing cardiovascular mortality and heart failure-related hospitalizations. However, chronic diuretic use carries potential risks, including an increased risk of hyperkalemia, with age-related differences in adverse event risks [older adults (≥ 65 years) facing higher risks, while younger populations do not show similar concerns].

**Message:**

Further research is needed to establish diuretic efficacy and safety in populations commonly seen in clinical practice, especially older adults living with multimorbidity and frailty.

**Supplementary Information:**

The online version contains supplementary material available at 10.1007/s41999-025-01229-5.

## Graphical abstract

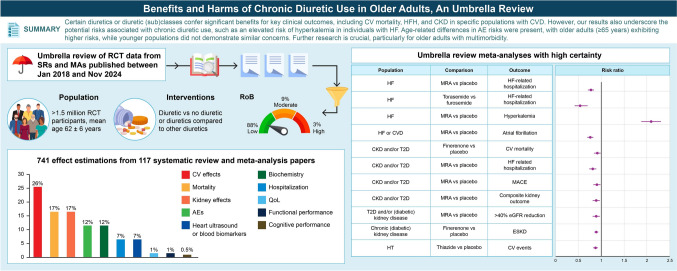

## Introduction

Cardiovascular disease (CVD) is a leading cause of morbidity and mortality globally, disproportionately affecting older individuals [1, 2]. It is a primary cause of both death and disability in aging populations, with a particularly high burden in those aged ≥ 75 due to age-related physiological changes [3]. Diuretics have long been a cornerstone in the management of heart failure (HF) [4] and hypertension (HT) [5] and are widely used worldwide to prevent CVD and mitigate end-organ damage [5, 6].

Diuretic use can cause significant harm, particularly in older adults or those with limited life expectancy [7–10]. These individuals often derive less benefit from medications compared to younger, healthier individuals as they face competing risks such as premature death before any therapeutic benefits can be realized. Prolonged diuretic use in the absence of fluid retention lacks substantial evidence for benefit and may be associated with potentially life-threatening adverse events (AEs). These include interference with the guideline-directed up-titration of evidence-based HF medications [11, 12], the development of cardiorenal syndrome, diuretic resistance [13], or neurohumoral hyperactivation, especially in frail older adults [14–16]. Additional instances of inappropriate diuretic prescribing arise when diuretics are prescribed as part of an inappropriate prescribing cascade [17–19], are continued beyond the necessary duration, or prescribed at excessive doses [20]. Observational studies have shown that inappropriate loop diuretic use is prevalent among older adults [21, 22]. For example, a study using the STOPP (Screening Tool of Older People's Prescriptions) criteria [23] found that 26% of potentially inappropriate medications identified in older adults receiving home-based medical services were related to diuretic prescriptions [22]. Inappropriate diuretic prescribing is linked to higher risks of hospitalization and mortality [14, 24].

When continued chronic diuretic use is deemed potentially inappropriate, treatment options, such as dose reduction, withdrawal (deprescribing), or switching to a safer alternative, should be considered. Deprescribing involves the careful, supervised withdrawal of medications when the risks outweigh the benefits, taking into account individual factors such as patient preferences, goals of care, and remaining life expectancy [25–28]. Recent reviews suggest that diuretic deprescribing is a safe and feasible option for carefully selected individuals, potentially preventing AEs, but evidence on the long-term outcomes of deprescribing diuretics is limited [15, 29]. Undertreatment should be avoided as congestion is a key contributor to symptoms, poor quality of life (QoL), and adverse outcomes, particularly in persons with HF [30]. Furthermore, diuretics were among the most frequently mentioned medications that older adults would like to have deprescribed, according to a recent survey of 1,340 individuals across 14 European countries [31]. One of the main reasons older adults are eager to deprescribe diuretics is that these medications, by promoting urinary incontinence, can be burdensome, limiting social interactions and outdoor activities [32]. Thus, clinicians must routinely evaluate the ongoing use of diuretics in older adults, and guide discussions with their patients regarding continued prescribing, not prescribing or deprescribing based on thorough knowledge of the available evidence regarding the risks and benefits of chronic diuretic use.

Numerous systematic reviews (SRs) and meta-analyses (MAs) have been published, addressing the effects of chronic diuretic use on health outcomes across various populations. Although necessary to ensure patient-centered, appropriate diuretic prescribing [30], a comprehensive synthesis and critical evaluation of this published evidence is currently lacking. To address this gap, we conducted an umbrella review of SRs and MAs published from January 1, 2018, assessing the breadth, credibility, and certainty of associations between diuretic use and health outcomes through RCTs. A deeper understanding of the benefits and harms of chronic diuretic therapy, especially in older adults, is essential for optimal diuretic prescribing.

## Methods

We conducted an umbrella review following guidance from the Cochrane Handbook, Chapter V: Overviews of Reviews [33] and followed the reporting guideline for overviews of reviews of healthcare interventions (PRIOR statement) [34]. The protocol for this study was prospectively registered on PROSPERO, the international prospective register of SRs (CRD42023423486).

### Search strategy

The search strategy was developed in consultation with an experienced medical librarian. We systematically searched MEDLINE, Embase, and the Cochrane Library (CDSR and CENTRAL) for publications from January 1, 2018 to November 11, 2024 to identify SRs and MAs of RCTs examining associations between chronic diuretic use and health outcomes in adults. The search strategy is presented in Supplementary Table 1. Database searches were supplemented by reference list checking of the included references.

### Inclusion and exclusion criteria

Two independent reviewers screened titles/abstracts and selected relevant records for eligibility. We considered SRs and MAs for inclusion, comparing the effects of chronic diuretic use with placebo or control, and the effects of diuretics (or subclasses) compared to the effects other diuretics (or subclasses) in adults (≥ 18 years). We considered all diuretics listed in the WHO (World Health Organization) ATC (Anatomical Therapeutic Chemical) classification code list, except vaptans. Language was restricted to English. We excluded MAs that investigated cost-effectiveness. Our PICOS (participants, intervention, control, outcomes, and study design) is presented in Supplementary Table 2.

### Data collection and data synthesis

Relevant aggregated data from the included SRs were extracted into a predefined data collection form by two independent reviewers. The two independent reviewers discussed their conflicts until a final consensus was reached. When consensus was not reached, a third senior independent reviewer made the final decision. We extracted quantitative meta-analytic data together with the corresponding 95% confidence intervals (95% CI). If SRs examined more than one health outcome, each outcome was recorded separately.

We categorized the collected data based on population or indication for diuretic use, based on diuretic subclass and health outcome. If, for a certain specific outcome, there were ≥ 3 effect estimates originating from ≥ 2 SRs, reporting on a similar diuretic subclass within a comparable population and comparison group, we performed MAs (see paragraph on statistical analysis). In summary-of-findings (SoF) tables, we summarized the extracted data (quantitatively for MAs, narratively for data for which pooling was not appropriate). Per outcome (sub)category, we analyzed the data. We included data on age-related subgroup and meta-regression analyses.

### Statistical analysis

The statistical analyses were performed with Comprehensive Meta-Analysis Version 4, (Borenstein, M., Hedges, L., Higgins, J., & Rothstein, H., Biostat, Englewood, New Jersey, United States of America). For each MA, we estimated summary effect sizes and 95% CIs using random-effects models [35, 36] and estimated between-study heterogeneity was assessed using the I^2^ metric. I^2^ ranges between 0 and 100% and is the ratio of between-study variance over the sum of the within-study and between-study variances. Values exceeding 50 or 75% are generally considered large or very large heterogeneity, respectively [37]. In addition, we estimated the 95% prediction interval, which accounts for the between-study heterogeneity and evaluates the uncertainty for the effect expected in a new study addressing the same association [38, 39].

To ensure high quality and rigor of our work, we accounted for publication overlap in SRs and MAs (reviews or analyses including the same primary publications). We tabulated the RCTs from the included MAs that were originally involved in each review and calculated the pairwise overlap; the corrected covered area (CCA) [40, 41]. We visualized and quantified overlap for each MA using the GROOVE (Graphical Representation of Overlap for OVErviews) tool [42]. We classified overlap as “slight” (< 5%), “moderate” (5 to < 10%), “high” (10 to < 15%), or “very high” (≥ 15%).

### Risk of bias and quality assessment

Two independent reviewers assessed the risk of bias (RoB) of the included SRs according to the standardized JBI (Joanna Briggs Institute) critical appraisal checklist for SRs and research syntheses (available from: https://joannabriggs.org/critical-appraisaltools). In this checklist, each of the 11 questions about the study’s methodology must be answered with one in four options: yes (Y), no (N), unclear (U), or not applicable (N/A). Disagreements in scoring were resolved by discussion between the two independent reviewers, and further discussed with a third independent reviewer until we reached consensus.

The overall RoB for each SR included in this umbrella review was calculated via merging the number of questions answered as “yes” in high, moderate or low RoB. If any question was answered with “not applicable”, it was not considered in the calculation of bias risk. Studies were classified as: low ≥ 70%, moderate between 50 and 70%, and high RoB with a score ≤ 49%. We performed sensitivity analyses, excluding data from SRs with high RoB.

### Grading the evidence

We evaluated the evidence from our MAs applying the GRADE (Grading of Recommendations, Assessment, Development and Evaluation) assessment criteria [43] to classify the certainty of the evidence as i: high (we are confident that the true effect is similar to the estimated effect); ii. moderate (we expect the true effect to be close to the estimated effect); iii. low (we expect the true effect to be fairly different from the estimated effect) or vi: very low (we expect the true effect to be markedly different from the estimated effect (GRADEpro version 3.6.1; McMaster University, ON, Canada). Findings began as high certainty evidence, we downgraded by one point to moderate certainty evidence if i: overall RoB was “moderate”, and two points to low certainty if “high” in at least 50% of MAs, or ii: if there was inconsistency in the net effect (e.g., neutral in some, but lower in others). For statistically significant differences in continuous effect size estimations [mean differences (MDs), standardized MDs (SMDs), or weighted MDs (WMDs)], we additionally rated clinical relevance based on reports from the international guidelines or reports from the literature [e.g., minimally clinically important differences (MCIDs)].

## Results

### Search and descriptive results

**Fig. 1:**
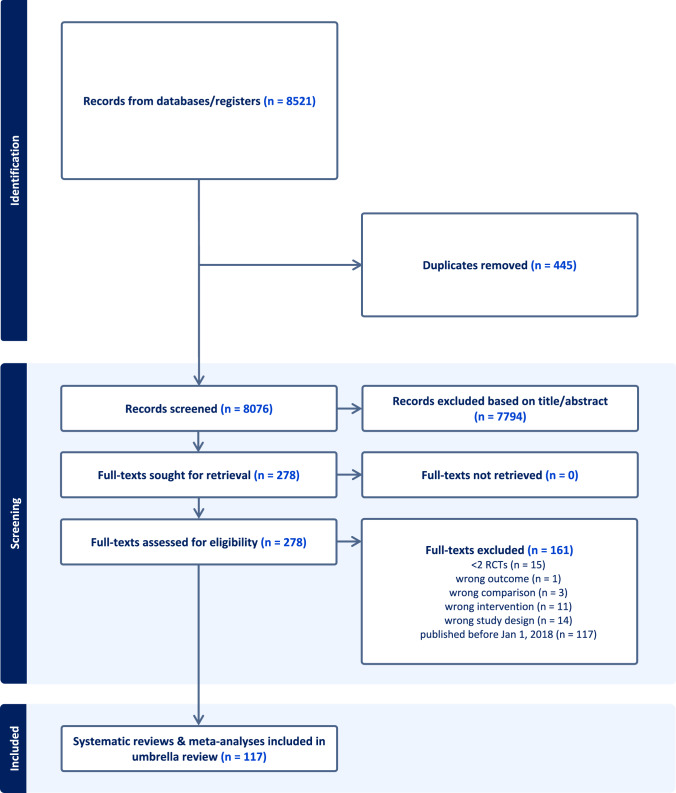
PRISMA flow diagram

Our search yielded 8076 unique results of which we deemed 278 suitable to cross-check for inclusion. After excluding a total of 161 records that did not match our inclusion criteria, we included 117 SR articles in our umbrella review (Fig. 1), reporting on 1566 RCTs among over 1.5 million participants treated with diuretics with a mean age of 62 ± 6 years. Characteristics of the included SRs are presented in Table 1. Overall RoB of the included SRs was low in 88%, moderate in 9%, and high in 3% (Supplementary Table 3).

**Table 1:** Included systematic review and meta-analysis articles

Review first author, publication year	Population, setting	Comparison	Number of individuals studied	Follow-up duration (mean/median or range)	Age of individuals (in years, mean/median or range)	Percentage females	Number of included RCTs on diuretics (K)
Abdelazeem et al., 2022 [77]	CKD and T2D	Finerenone vs placebo	13,847	1.6 years	64.7 ± 8.7	30	3
Abraham et al., 2020 [78]	CHF	Torasemide vs furosemide	–	15 months	–	–	9
Ahmed et al., 2023 [79]	HT (resistant)	MRA vs placebo	1414	16 weeks	58.89 ± 5.05	45	8
Albasri et al., 2021 [80]	HT	Thiazide vs placebo	24,311	2 to 4 years	–	–	5
Alexandre et al., 2019 [48]	not specified	MRA vs placebo	2843	12 months	64.2	–	11
Alexandrou et al., 2019 [81]	CVD	MRA vs placebo/active control	2767	–	–	–	31
Al-Sadawi et al., 2024 [82]	HFpEF	MRA vs placebo	1426	31 months	71	50	4
Asiimwe et al., 2021 [83]	not specified	Diuretic vs no diuretic	85,555	–	–	–	19
Bao et al., 2022 [84]	DKD	Finerenone vs placebo	13,510	–	–	–	4
Bazoukis et al., 2018a [85]	HFpEF and HFrEF	MRA vs placebo	13,354	9.4 months	66,3	34	7
Bazoukis et al., 2018b [86]	HT (resistant)	MRA vs placebo/active control	2736	4.83 months	58.5	36	21
Bidel et al., 2023 [49]	not specified	Thiazide vs placebo	–	4.2 years	66.2 ± 9.8 (women); 64.2 ± 9.4 (men)	42	–
Bonsu et al., 2018 [87]	HFpEF	MRA vs placebo	5238	3 to 40 months	67.7	59	12
Boulmpou et al., 2022 [88]	HFpEF	Spironolactone vs placebo	815	7 days to 2 years	–	–	4
Chen et al., 2024 [89]	CKD	Non-steroidal MRA or eplerenone vs placebo	15,817	–	–	–	11
Clark et al., 2024 [90]	HFpEF and HFrEF	MRA vs placebo	–	–	–	–	5
Desbiens et al., 2022 [50]	HT	Thiazide vs placebo/no treatment	68,107	4 weeks to 5.6 years	34 to 84 years	46	30
Dineva et al., 2019 [91]	HT	Chlorthalidone vs HCTZ	51,789	4 to 364 weeks	–	–	7
Dineva et al., 2020 [92]	HT	HCTZ vs placebo; chlorthalidone vs placebo; HCTZ vs chlorthalidone	–	4 to 364 weeks	–	–	33
Ding et al., 2023 [93]	CKD	MRA (network meta-analysis)	15,531	–	–	–	26
Du et al., 2024 [94]	CKD and T2D	Finerenone vs placebo	14,875	3 to 40.8 months	66.3	30	4
Dutta et al., 2022 [95]	DKD	Finerenone vs placebo/active control	–	42 days to 3.4 years	–	–	7
Eid et al., 2021 [96]	CHF	Furosemide vs other loop diuretics	2709	3 to 728 days	–	–	34
Elshahat et al., 2024 [97]	CHF	Eplerenone vs spironolactone	21,930	20 months	58.1	–	4
Faisal et al., 2022 [98]	HFpEF	Spironolactone vs placebo	–	12 or 40 months	–	–	2
Farmakis et al., 2022 [99]	HT	Thiazide vs placebo	7784	8 weeks to 5.8 years	56.2 ± 9.6	0	16
Fatima et al., 2023 [100]	not specified	MRA vs control	11,356	8 days to 38 months	66	–	10
Fernandes et al., 2018 [101]	HFpEF	MRA vs placebo	725	6 to 26 months	65	–	5
Ferre et al., 2021 [102]	Idiopathic hypercalciuria	Diuretic vs placebo/control	446	–	–	–	4
Frankenstein et al., 2020 [103]	HFrEF (NYHA class II to IV)	MRA vs placebo/standard medical care	12,213	4 to 60 months	46 to 70	–	14
Fu et al., 2021 [104]	CKD	Finerenone vs placebo	7048	1 to 30 months	–	–	4
Fukuta et al., 2019 [105]	HFpEF	MRA vs placebo	755	6 to 12 months	–	–	6
Geng et al., 2023 [51]	CHF	MRA vs placebo	9056	2.1 years	68	–	7
Ghosal and Sinha 2022b [106]	T2D	Finerenone vs placebo	13,026	–	–	–	2
Ghosal and Sinha 2023a [107]	CKD and T2D	Finerenone vs placebo	13,943	3 to 40.8 months	–	–	4
Gu et al., 2024 [108]	T2D	Finerenone vs placebo	13,847	–	59 to 66	65	3
Hall et al., 2020 [109]	HT	Thiazide vs placebo	76,608	35 weeks	58	45	95
Hansen et al., 2020 [110]	HFrEF	MRA vs placebo	–	1.6 to 4.1 years	–	–	3
Harrington et al., 2023 [111]	HFrEF	MRA vs placebo	–	–	–	–	3
Hasegawa et al., 2021 [112]	CKD	MRA vs placebo/standard care	1446	–	–	–	16
Ho et al., 2024 [113]	primary hyperaldosteronism	Eplerenone vs spironolactone	392	–	43.5 to 59.5	32 to 60	5
Hu et al., 2022 [114]	DKD	Eplerenone vs placebo/active control	838	6 to 48 weeks	–	–	8
Jiang et al., 2022 [115]	CKD and T2D	MRA vs placebo	14,450	–	–	–	8
Jyotsna et al., 2023 [116]	CKD (stages 1 to 4) and T2D	Finerenone vs placebo	39,995	–	–	–	7
Kapelios et al., 2019 [117]	HFpEF	MRA vs placebo/control	1164	6 to 18 months	–	–	9
Karakasis et al., 2024 [118]	CVD	MRA vs placebo	13,358	–	48.7 to 73	59	23
Kido et al., 2019 [119]	HFpEF and HFrEF	Torasemide vs furosemide	–	–	–	–	2
Kohjimoto et al., 2024 [120]	kidney stones (calcium oxalate)	Thiazide vs no thiazide	571	–	–	–	8
Li et al., 2018 [52]	HFpEF	Spironolactone vs placebo	4147	6 to 39.6 months	62 to 71	52 to 100	7
Li et al., 2020 [121]	kidney stones	Thiazide vs placebo/no treatment	–	–	–	–	8
Li et al., 2022 [122]	CKD and T2D	Finerenone vs placebo	–	–	–	–	2
Li et al., 2024 [123]	CHF	Spironolactone vs placebo	–	–	–	–	2
Liu et al., 2022 [124]	ESKD requiring dialysis	Spironolactone vs placebo/standard medical therapy	1258	2 weeks to 3 years	52.92 ± 6.90 to 70.45 ± 9.70	–	15
Lunney et al., 2020 [125]	CHF and CKD	MRA vs placebo	826	6 to 24 months	–	–	3
Ma et al., 2021 [126]	HT	ARB/HCTZ vs ARB alone	–	4 to 12 weeks	–	–	16
Macfarlane et al., 2019 [127]	HT (primary)	Indapamide vs bendroflumethiazide	21,315	18 months to 5 years	–	–	3
Martin et al., 2021 [128]	HFpEF	MRA vs placebo/no treatment	4459	24 weeks to 3.3 years	54.5 to 80	–	13
Martins et al., 2023 [129]	HT (primary)	Thiazide vs placebo	58,807	10 weeks	55	45	276
Miles et al., 2019 [130]	CHF	Torasemide vs furosemide	8127	5 to 12 months	63 to 75	22 to 58	10
Morita et al., 2022 [131]	HT and DKD	MRA vs placebo; MRA/SGLT2i vs SGLT2i alone	–	–	–	–	8
Musini et al., 2019 [44]	HT	Diuretic vs placebo/no treatment	–	3.8 years	–	–	8
Nguyen et al., 2023 [132]	CKD and DKD	Non-steroidal MRA vs placebo	6519	24 to 278 weeks	–	–	2
Noone et al., 2020 [133]	HT	Thiazide-like vs placebo	–	> 4 weeks	–	–	93
Oraii et al., 2024 [134]	CHF	MRA vs placebo	21,791	33.3 months	65.2	31	7
Pamporis et al., 2024 [135]	HFrEF	MRA vs control	15,685	4 to 540 weeks	–	–	32
Patoulias et al., 2021 [136]	CVD	MRA vs placebo	9866	–	–	–	6
Patoulias et al., 2022 [137]	CKD and T2D	Finerenone vs placebo	13,036	–	–	–	2
Peters et al., 2020 [138]	HT	Diuretic vs placebo/no treatment	–	> 1 year and > 5 years	–	–	4
Sakima et al., 2021 [53]	HT	MRA vs placebo/active control	877	4 weeks to 2 years	34 to 77	–	17
Sampaio Rodrigues et al., 2024 [139]	not specified	MRA vs placebo/standard medical therapy	11,419	6 months to 3.3 years	–	–	12
Seeley et al., 2020 [54]	HT	Diuretic vs placebo	–	–	51.2 ± 5.72	58	17
Shah et al., 2018 [60]	CHF	Torasemide vs furosemide	664	11 months	67,2	57	3
Shaman et al., 2020 [55]	HT	MRA vs placebo	4283	6 months	57	39	40
Sherif et al., 2019 [70]	CHF	Torasemide vs furosemide	–	–	55 to 82.3	–	14
Shi et al., 2023 [140]	T2D	Non-steroidal MRA vs standard treatment	471,038	6.0 months	57,7	43	–
Siddiqi et al., 2023 [141]	CHF	Furosemide vs torasemide	1996	240 days	58.2 to 75.1	–	13
Singh et al., 2023 [142]	CHF	Furosemide vs torasemide	4127	3 to 18 months	68	44	10
Sreenivasan et al., 2024 [143]	HFpEF	MRA vs placebo	–	15 months	71.7 ± 4.2	49	6
Sreenivasan et al., 2022 [144]	HFpEF	MRA vs placebo	–	15 months	62.4 to 80	49	6
Täger et al., 2019 [145]	CHF (systolic)	Diuretic vs placebo	2647	–	56 to 76	–	34
Teixeira et al., 2024 [146]	CHF	Torasemide vs furosemide	4115	–	–	–	12
Teles et al., 2023 [147]	HT	Thiazide(like) vs loop diuretic	–	24 days to 20 weeks	53.7 ± 7.8 to 67.5 ± 10.2	–	4
Thomopoulos et al., 2018 [47]	HT	Diuretic vs placebo	39,751	–	–	–	10
Tian et al., 2023 [148]	HT (resistant)	Spironolactone vs placebo	–	14.5 weeks	59 ± 3.68	39	7
Tsukamoto et al., 2022 [149]	CKD and DKD	MRA vs placebo	36,186	9.2 months to 4.2 years	–	–	3
Wang et al., 2023 [150]	CHF	MRA vs placebo	14,698	–	–	27	5
Wei et al., 2020 [151]	CVD	Thiazide vs placebo/standard medical treatment	–	> 6 months	–	–	13
Wright et al., 2018 [152]	HT (primary)	Thiazide vs placebo/no treatment	39,713	4.1 years	61	–	19
Wu et al., 2022 [153]	DKD	MRA vs placebo	–	12 to 54 weeks	45.6 ± 13.1 to 65.4 ± 8.9	–	12
Xiang et al., 2019 [154]	HFmrEF and HFpEF	Spironolactone vs placebo/standard medical therapy	4539	4 to 39 months	58 to 71.55	–	11
Xiang et al., 2022 [155]	HFmrEF and HFpEF	MRA vs placebo	–	6 to 39.6 months	66.3 to 76.4	–	5
Xie et al., 2018 [156]	HT	Diuretic vs placebo	–	–	–	–	2
Xu et al., 2018 [157]	not specified	MRA vs placebo/standard medical therapy	11,365	1 to 24 months	52 ± 10 to 64 ± 11	–	13
Xu et al., 2024 [158]	CKD and T2D	Non-steroidal MRA vs placebo	14,997	–	–	–	7
Yanai et al., 2021 [159]	CKD	MRA vs placebo	–	> 13 weeks	–	–	4
Yang et al., 2019 [160]	CHF (EF ≤ 45%)	MRA vs placebo; finerenone vs spironolactone; finerenone vs eplerenone; spironolactone vs eplerenone	13,597	1 to 24 months	–	–	13
Yang et al., 2022 [161]	CKD and T2D	Non-steroidal MRA vs placebo; eplerenone vs placebo; spironolactone vs placebo; non-steroidal MRA vs Nonselective MRA	15,025	–	–	–	12
Yang et al., 2023 [162]	CKD and T2D	Finerenone vs placebo	13,679	90 days to 3.4 years	62.4 to 66.8	20 to 30	4
Yang et al., 2024 [163]	DKD	Non-steroidal MRA vs placebo	–	–	–	–	5
Yang et al., 2023 [164]	CKD and DKD	MRA vs placebo	–	2 years	65.8	39	5
Yasmin et al., 2023 [165]	CKD	Finerenone vs placebo	15,462	–	–	–	7
Yi et al., 2024 [57]	HT	MRA or thiazide vs placebo	7758	–	–	–	15
Yuan et al., 2023 [56]	CKD	MRA vs control	22,792	2 to 42 months	34 to 74	0 to 81	53
Zafeiropoulos et al., 2024 [166]	HFmrEF and HFpEF	MRA vs placebo	–	33 months	71.4 ± 9.0	48	–
Zeng et al., 2019 [167]	ESRD	Spironolactone vs control	765	2 weeks to 3 years	–	–	7
Zhang et al., 2019 [45]	HT	Diuretic vs placebo	–	1.8 to 5.8 years	–	–	2
Zhang et al., 2022a [168]	CKD and T2D	Finerenone vs placebo	14,847	–	–	–	3
Zhang et al., 2022b [169]	CKD and T2D	Finerenone vs placebo	14,847	–	–	–	3
Zhang et al., 2022c [170]	CKD	Finerenone vs placebo	–	14 to 33 days	–	–	5
Zhao et al., 2019 [171]	CHF	Furosemide vs other loop diuretics	1956	–	–	–	12
Zheng et al., 2018 [172]	HFpEF	MRA vs placebo	4003	6 to 49.5 months	–	–	5
Zheng et al., 2022 [173]	DKD	Finerenone vs placebo	–	3.4 years	58.08 ± 13.08 to 66.75 ± 9.02	–	4
Zhong et al., 2021 [46]	CVD	Diuretic vs placebo	35,543	–	–	–	8
Zhu et al., 2020 [174]	DKD	Diuretic vs placebo	360	1 to 12 months	45 ± 5 to 60	–	5
Zhu et al., 2021 [175]	not specified	Spironolactone or eplerenone vs placebo	1128	3 to 36 months	–	–	9
Zhu et al., 2022 [176]	CKD and/or diabetes	Finerenone vs eplerenone/placebo	15,618	18 days to 53 months	–	–	7
Zonneveld et al., 2018 [177]	HT	Diuretic vs no diuretic	6216	–	–	–	3

*ARB* angiotensin receptor blocker, *CHF* chronic heart failure, *CKD* chronic kidney disease, *CVD* cardiovascular disease, *DKD* diabetic kidney disease, *EF* ejection fraction, *HCTZ* hydrochlorothiazide, *HFmrEF* heart failure with mid-range ejection fraction, *HFpEF* heart failure with preserved ejection fraction, *HFrEF* heart failure with reduced ejection fraction, *HT* hypertension, *MRA* mineralocorticoid antagonist, *NYHA* New York Heart Association, *RCT* randomized-controlled trial, *SGLT2i* sodium-glucose cotransporter-2 inhibitor, *T2D* type 2 diabetes mellitus

From the 117 SR papers, we extracted 741 effect estimations, 558 (75%) comparing diuretics and no diuretics (majority: placebo; Table 2 and Supplementary Table 4), and 183 (25%) comparing different diuretics (or subclasses; Table 3 and Supplementary Table 5). Of the 741 effect estimations, 192 (26%) were on cardiovascular (CV) effects, 125 (17%) on mortality, 123 (17%) on kidney effects, 89 (12%) on various AEs, 86 (12%) on biochemical effects, 54 (7%) on hospitalization, 35 (5%) on heart ultrasonography measures, 15 (2%) on heart biomarkers, 9 (1%) on QoL, 8 (1%) on functional performance, and 4 (0.5%) on cognitive performance. Of the 319 included placebo-controlled effect estimations, 285 (89%) were on different mineralocorticoid receptor antagonists (MRAs) or subclasses, 19 (6%) on thiazide(like) diuretics, 12 (4%) on diuretics (unspecified), and 3 (0.9%) on loop diuretics. Two SRs exclusively focused on diuretic effects in older (≥ 60 years) individuals [44, 45]; 12 SRs reported on potential age-related differences (either as main objective or based on predefined subgroup/meta-regression analyses) [45–56].

**Table 2:** Summary of findings table, meta-analyses comparing diuretic therapy, and placebo

Review first author, year of publication	Outcome category	Specific outcome	Diuretic indication, population	Diuretic intervention	Comparison	Effect (metric, 95% CI)	Meta-analysis (number of pooled effect estimates, metric, 95% CI, *I*^2^)	GRADE (reasons for downgrading)	Overall overlap
Frankenstein et al., 2020 [103]	Biochemistry	Hyperkalemia	CHF	Canrenone	Placebo or standard medical care	HR 2.35 (1.13 to 4.87)	**Hyperkalemia** Patients with HF, MRA vs placebo: *n* = 4, RR 2.09 (1.87 to 2.33; *p* < 0.001, *I*^2^ = 0%)	High	Moderate
Geng et al., 2023 [51]				MRA	Placebo	RR 2.06 (1.78 to 2.39)			
Frankenstein et al., 2020 [103]				Eplerenone	Placebo or standard medical care	HR 2.15 (0.91 to 5.07)			
Martin et al., 2021 [128]			HFpEF	MRA	Placebo	RR 2.11 (1.77 to 2.52)			
Tsukamoto et al., 2022 [149]	Biochemistry	Hyperkalemia	CKD and T2D	MRA	Placebo	RR 2.06 (1.79 to 2.37)	**Hyperkalemia** Patients with kidney disease, MRA vs placebo: *n* = 16, RR 2.31 (2.07 to 2.58; *p* < 0.001, *I*^2^ = 69%)	High	Low
Lunney et al., 2020 [125]			CKD			RR 2.91 (2.03 to 4.17)			
Jiang et al., 2022 [115]			CKD and T2D			RR 2.07 (1.86 to 2.30)			
Hu et al., 2022 [114]			DKD	Eplerenone		RR 1.55 (0.88 to 2.72)			
Zheng et al., 2022 [173]				Finerenone		RR 2.03 (1.83 to 2.26)			
Yang et al., 2024 [163]				MRA, non-steroidal		RR 1.01 (0.40 to 3.02)			
Hasegawa et al., 2021 [112]			ESKD requiring dialysis	MRA		RR 1.41 (0.72 to 2.78)			
Alexandrou et al., 2019 [81]			proteinuric kidney disease			RR 4.44 (1.99 to 9.93)			
Wu et al., 2022 [153]			proteinuric kidney disease in diabetes	Eplerenone		RR 1.6 (0.57 to 6)			
				Esaxerenone		RR 4.1 (1.8 to 11)			
				Spironolactone		RR 8.4 (3.2 to 36)			
Ghosal et al., 2022 [106]			T2D	Finerenone		RR 2.22 (1.93 to 2.54)			
Chen et al., 2024 [89]			CKD	MRA, non-steroidal		RR 2.05 (1.85 to 2.28)			
Jyotsna et al., 2023 [116]			CKD and T2D	Finerenone		RR 2.20 (1.90 to 2.55)			
		Hyperkalemia, serious				RR 4.25 (3.11 to 5.83)			
Zhang et al., 2022 [169]						RR 4.08 (2.39 to 6.96)			
Al-Sadawi et al., 2024 [82]	Biochemistry	Hyperkalemia	HFpEF	MRA	Placebo	OR 3.90 (1.38 to 11.01)	**Hyperkalemia** Patients with HF, MRA vs placebo: *n* = 3, OR 1.82 (1.30 to 2.55; *p* = 0.001, *I*^2^ = 62%)	Moderate (inconsistency)	Low
Harrington et al., 2023 [111]		Hyperkalemia, serious	HF			OR 1.46 (1.16 to 1.82)			
		Hyperkalemia				OR 1.97 (1.51 to 2.59)			
Yang et al., 2019 [160]	Biochemistry	Hyperkalemia	CKD and T2D	Canrenone	Placebo	OR 3.3 (1.2 to 10)	**Hyperkalemia** Patients with CKD with and without T2D, MRA vs placebo: *n* = 7, OR 2.26 (1.96 to 2.61; *p* < 0.001, *I*^2^ = 17%)	Moderate (inconsistency)	Moderate
Yasmin et al., 2023 [165]		Hyperkalemia, mild	CKD	Finerenone		OR 1.76 (0.68 to 4.52)			
		Hyperkalemia, moderate				OR 2.11 (1.77 to 2.52)			
Zhu et al., 2020 [174]		Hyperkalemia, new-onset	DKD	Diuretics (nonspecified)		OR 1.8 (0.69 to 4.5)			
				MRA		OR 3.34 (1.1 to 7.13)			
Xu et al., 2024 [158]		Hyperkalemia	CKD and T2D	MRA, non-steroidal		OR 2.27 (1.90 to 2.71)			
Yang et al., 2019 [160]				Spironolactone		OR 3.6 (2.3 to 7.4)			
				Eplerenone		OR 1.8 (1.2 to 3)			
Bazoukis et al., 2018 [86]	Cardiovascular	DBP	HF	Eplerenone	Placebo	MD − 1 (− 4.7 to − 2.0)	**DBP** Patients with HF, MRA vs placebo: *n* = 4, SMD − 0.20 (− 0.39 to 0.003 mmHg; *p* = 0.054, *I*^2^ = 51%)	Moderate (inconsistency)	Moderate
Fukuta et al., 2019 [105]			HFpEF	MRA		WMD − 3.58 (− 5.65 to − 1.52)			
Bazoukis et al., 2018 [86]			HF			MD − 0.34 (− 3.37 to 2.68)			
				Spironolactone		MD − 2.99 (− 7.3 to − 2.5)			
Bazoukis et al., 2018 [86]	Cardiovascular	SBP	HF	Eplerenone	Placebo	MD − 0.04 (− 4.4 to 4.3)	**SBP** Patients with HF, MRA vs placebo: *n* = 4, SMD − 0.33 (− 0.68 to 0.024 mmHg; *p* < 0.001, *I*^2^ = 84%)	Low (inconsistency and imprecision)	Moderate
Fukuta et al., 2019 [105]			HFpEF	MRA		WMD − 8.39 (− 11.38 to − 5.41)			
Li et al., 2024 [123]			HF	Spironolactone		WMD − 2.37 (− 3.81 to − 0.94)			
Bazoukis et al., 2018 [86]						MD − 4.77 (− 22.3 to − 9.9)			
Wright et al., 2018 [152]	Cardiovascular	SBP	HT, first-line therapy	Thiazide, high-dose	Placebo or no anti-hypertensive treatment	MD − 13.66 (− 14.4 to − 12.91)	**SBP** Patients with HT, thiazide vs placebo: *n* = 3, SMD − 4.23 (− 6.40 to − 2.10 mmHg; *p* < 0.001, *I*^2^ = 98%)	High	Moderate
				Thiazide, low-dose		MD − 12.56 (− 13.22 to − 11.91)			
Martins et al., 2023 [129]			HT, primary	Thiazide	Placebo	MD − 10.37 (− 11.64 to − 9.10)			
Jiang et al., 2022 [115]	Cardiovascular	SBP	CKD and T2D	MRA	Placebo	WMD − 4.48 (− 5.95 to − 3.72)	**SBP** Patients with CKD, MRA vs placebo: *n* = 6, SMD − 0.51 (− 0.87 to − 0.14 mmHg; *p* = 0.006, *I*^2^ = 90%)	Moderate (imprecision)	Low
Wu et al., 2022 [153]			Proteinuric kidney disease in Diabetes	Spironolactone		MD − 18 (− 42 to 6.4)			
Xu et al., 2024 [158]			CKD and T2D	MRA, non-steroidal		WMD − 4.34 (− 5.28 to − 3.41)			
Hu et al., 2022 [114]			DKD	Eplerenone		MD − 2.49 (− 4.48 to − 0.5)			
Shaman et al., 2020 [55]			ESKD requiring dialysis	MRA		MD − 8.5 (− 16.4 to − 0.5)			
Wu et al., 2022 [153]			Proteinuric kidney disease in diabetes	Finerenone		MD − 5.3 (− 35 to 25)			
Karakasis et al., 2024 [118]	Cardiovascular	Atrial fibrillation	CVD	Spironolactone	Placebo	RR 0.76 (0.65 to 0.89)	**Atrial fibrillation** Patients with HF or CVD, MRA vs placebo: *n* = 6, RR 0.76 (0.71 to 0.82; *p* < 0.001, *I*^2^ = 0%)	High	Moderate
		Atrial fibrillation, recurrent		MRA		RR 0.75 (0.63 to 0.89)			
Oraii et al., 2024 [134]			HF			RR 0.74 (0.63 to 0.87)			
Karakasis et al., 2024 [118]		Atrial fibrillation, new-onset	CVD			RR 0.79 (0.64 to 0.97)			
Oraii et al., 2024 [134]			HF			RR 0.81 (0.64 to 1.03)			
		Atrial fibrillation				RR 0.76 (0.67 to 0.87)			
Sampaio Rodrigues et al., 2024 [139]	Cardiovascular	Atrial fibrillation, new-onset	not specified	MRA	Placebo or standard medical care	OR 0.77 (0.55 to 1.06)	**Atrial fibrillation** Patients regardless of diuretic indication, MRA vs placebo: *n* = 8, OR 0.58 (0.47 to 0.72; *p* < 0.001, *I*^2^ = 48%)	Moderate (inconsistency)	Moderate
Alexandre et al., 2019 [48]				Canrenone	Placebo	OR 0.23 (0.12 to 0.44)			
				Eplerenone		OR 0.58 (0.35 to 0.96)			
				MRA		OR 0.52 (0.37 to 0.73)			
Sampaio Rodrigues et al., 2024 [139]		Atrial fibrillation, new-onset or recurrent		Eplerenone	Placebo or standard medical care	OR 1.08 (0.34 to 3.51)			
				MRA		OR 0.68 (0.51 to 0.92)			
				Spironolactone		OR 0.63 (0.40 to 0.98)			
		Atrial fibrillation, recurrent		MRA		OR 0.50 (0.30 to 0.83)			
Clark et al., 2024 [90]	Cardio vascular	Composite	HFpEF	MRA	Placebo	RR 1.16 (0.86 to 1.57)	**Composite CV outcome** Patients with HF, MRA vs placebo: *n* = 4, RR 0.90 (0.76 to 1.07; *p* = 0.231, *I*^2^ = 58%)	Moderate (inconsistency)	Moderate
			HFrEF			RR 0.96 (0.81 to 1.14)			
Wang et al., 2022 [150]		Composite (men and women)	HF			RR 0.96 (0.77 to 1.19)			
		Composite (men)				RR 0.79 (0.69 to 0.91)			
		Composite (women)				RR 0.77 (0.52 to 1.16)			
Musini et al., 2019 [44]	Cardio vascular	CV events	HT, age > 60 years	Thiazide	Placebo	RR 0.67 (0.61 to 0.74)	**CV events** Patients with HT, thiazides vs placebo: *n* = 3, RR 0.85 (0.80 to 0.90; *p* < 0.001, *I*^2^ = 0%)	High	Low
Wright et al., 2018 [152]			HT, first-line therapy	Thiazide, high-dose	Placebo or no anti-hypertensive treatment	RR 0.72 (0.63 to 0.82)			
				Thiazide, low-dose		RR 0.7 (0.64 to 0.76)			
Wei et al., 2020 [151]			Not specified	Diuretic (nonspecified)	placebo	RR 0.73 (0.62 to 0.85)			
Zhang et al., 2022 [169]	Cardiovascular	MACE	CKD	Finerenone	Placebo	RR 0.89 (0.75 to 1.05)	**MACE** Patients with CKD and/or T2D MRA vs placebo: *n* = 6, RR 0.88 (0.83 to 0.93; *p* < 0.001, *I*^2^ = 0%)	High	Low
Zhang et al., 2022 [168]			CKD and T2D	Finerenone		RR 0.88 (0.8 to 0.97)			
Nguyen et al., 2023 [132]				MRA, non-steroidal		RR 0.92 (0.76 to 1.11)			
Li et al., 2022 [122]			T2D	Finerenone		HR 0.87 (0.72 to 1.04)			
Gu et al., 2024 [108]			T2D, established ASCVD			HR 0.92 (0.79 to 1.06)			
			T2D, without established ASCVD			HR 0.85 (0.77 to 0.95)			
Li et al., 2022 [122]	Cardio vascular	MI	T2D	Finerenone	Placebo	HR 0.9 (0.62 to 1.29)	**MI** Patients with CKD and/or T2D, finerenone vs placebo: *n* = 3, RR 0.90 (0.81 to 0.99; *p* = 0.044, *I*^2^ = 0%)	High	Low
Abdelazeem et al., 2022 [77]		MI, non-fatal	CKD and T2D			RR 0.91 (0.74 to 1.12)			
Jyotsna et al., 2023 [116]						RR 0.89 (0.78 to 1.02)			
Zhang et al., 2022 [168]	Cardiovascular	Stroke, non-fatal	CKD and T2D	Finerenone	Placebo	RR 1.00 (0.82 to 1.21)	**Stroke** Patients with CKD and/or T2D, MRA vs placebo: *n* = 5, RR 1.00 (0.92 to 1.10; *p* = 0.944, *I*^2^ = 0%)	High	Low
Abdelazeem et al., 2022 [77]		Stroke				RR 0.99 (0.80 to 1.22)			
Nguyen et al., 2023 [132]				MRA, non-steroidal		RR 1.00 (0.71 to 1.4)			
Li et al., 2022 [122]		Stroke, non-fatal	T2D	Finerenone		HR 1.00 (0.7 to 1.44)			
Jyotsna et al., 2023 [116]			CKD and T2D			RR 1.01 (0.89 to 1.14)			
Xiang et al., 2022 [155]	Hospitalization	HF-related	HFmrEF and HFpEF	MRA	Placebo	HR 0.83 (0.69 to 0.99)	**HF-related hospitalization** Patients with HF, MRA vs placebo: *n* = 6, RR 0.79 (0.75 to 0.83; *p* < 0.001, *I*^2^ = 0%)	High	High
Zafeiropoulos et al., 2024 [166]						HR 0.87 (0.68 to 1.12)			
Martin et al., 2021 [128]			HFpEF			HR 0.82 (0.69 to 0.98)			
Oraii et al., 2024 [134]			HF			HR 0.78 (0.73 to 0.84)			
Xiang et al., 2022 [155]			HFpEF and HFmrEF			HR 0.83 (0.69 to 0.99)			
Frankenstein et al., 2020 [103]			CHF	Canrenone	Placebo or standard medical care	HR 0.35 (0.12 to 0.99)			
				Eplerenone		HR 0.75 (0.66 to 0.84)			
Chen et al., 2024 [89]	Hospitalization	HF-related	CKD	MRA, non-steroidal	Placebo	RR 0.79 (0.67 to 0.92)	**HF-related hospitalization** Patients with CKD and/or T2D, MRA vs placebo: *n* = 8, RR 0.78 (0.73 to 0.82; *p* < 0.001, *I*^2^ = 0%)	High	Low
Zhang et al., 2022 [168]			CKD and T2D	Finerenone		RR 0.79 (0.67 to 0.92)			
Abdelazeem et al., 2022 [77]						RR 0.79 (0.66 to 0.94)			
Li et al., 2022 [122]			T2D			HR 0.78 (0.59 to 1.03)			
Gu et al., 2024 [108]						HR 0.78 (0.66 to 0.92)			
Jyotsna et al., 2023 [116]			CKD and T2D			RR 0.77 (0.70 to 0.84)			
Yang et al., 2023 [164]				MRA	Control	RR 0.79 (0.67 to 0.92)			
Tsukamoto et al., 2022 [149]					Placebo	RR 0.71 (0.57 to 0.9)			
Nguyen et al., 2023 [132]				MRA, non-steroidal		RR 0.78 (0.66 to 0.92)			
Fu et al., 2021 [104]	Kidney	eGFR	CKD	finerenone	Placebo	MD − 0.9 (− 3.84 to 2.04)	**eGFR** Patients with CKD and/or T2D, MRA vs placebo: *n* = 11, SMD − 0.40 (− 0.69 to − 0.11 ml/min per 1.73 m^2^; *p* = 0.007, *I*^2^ = 91%)	High	Low
Chen et al., 2024 [89]				MRA, non-steroidal		WMD − 2.83 (− 3.95 to − 1.72)			
Xu et al., 2024 [158]			CKD and T2D			WMD − 2.44 (− 4.06 to − 0.83)			
Jiang et al., 2022 [115]						WMD − 2.69 (− 4.47 to − 0.91)			
Hu et al., 2022 [114]			DKD	Eplerenone		MD 1.8 (− 0.83 to 4.43)			
Hu et al., 2022 [114]					Placebo or active control	MD 1.74 (− 0.87 to 4.35)			
Alexandrou et al., 2019 [81]			Proteinuric kidney disease	MRA	Placebo	MD − 2.82 (− 3.98 to − 1.66)			
Wu et al., 2022 [153]			Proteinuric kidney disease in diabetes	Esaxerenone		MD − 3.3 (− 11 to 4.5)			
				Finerenone		MD − 2.6 (− 9.7 to 4.7)			
				Spironolactone		MD − 5.9 (− 12 to 1.3)			
Ghosal et al., 2023			T2D	Finerenone		SMD − 0.32 (− 0.37 to − 0.27)			
Yasmin et al., 2023 [165]			CKD			MD − 0.02 (− 0.33 to 0.28)			
Fu et al., 2021 [104]	Kidney	UACR	CKD	Finerenone	Placebo	MD − 0.3 (− 0.5 to − 0.11)	**UACR** Patients with CKD and/or T2D, MRA vs placebo: *n* = 10, SMD − 1.31 (− 1.84 to − 0.77 mg/g; *p* = 0.007, *I*^2^ = 97%)	High	Low
Hu et al., 2022 [114]			DKD	Eplerenone		MD − 48.29 (− 64.45 to − 32.14)			
Zheng et al., 2022 [173]				Finerenone		MD − 0.3 (− 0.33 to − 0.27)			
Wu et al., 2022 [153]			Proteinuric kidney disease in diabetes	Apararenone		MD − 0.63 (− 0.9 to − 0.35)			
				Esaxerenone		MD − 0.54 (− 0.72 to − 0.3)			
				Finerenone		MD − 0.21 (− 0.5 to 0.071)			
Ghosal et al., 2023			T2D	Finerenone		SMD − 0.49 (− 0.53 to − 0.46)			
Chen et al., 2024 [89]			CKD	MRA, non-steroidal		WMD − 0.41 (− 0.49 to − 0.32)			
Xu et al., 2024 [158]			CKD and T2D			WMD − 0.39 (− 0.48 to − 0.31)			
Jiang et al., 2022 [115]				MRA		WMD − 0.40 (− 0.48 to − 0.32)			
Yang et al., 2024 [163]	Kidney	AKI	DKD	MRA, non-steroidal	Placebo	RR 1.08 (0.90 to 1.28)	**AKI** Patients with T2D and/or (diabetic) kidney disease, MRA vs placebo: *n* = 3, RR 0.97 (0.89 to 1.05; *p* = 0.42, *I*^2^ = 0%)	Moderate (inconsistency)	Low
Jyotsna et al., 2023 [116]			CKD and T2D	Finerenone		RR 0.94 (0.84 to 1.05)			
Ghosal et al., 2022 [106]		AKI or eGFR reduction ≥ 40%	T2D			RR 0.93 (0.78 to 1.11)			
Ding et al., 2023 [93]	Kidney	Composite	CKD	Finerenone	Placebo	HR 0.84 (0.77 to 0.92)	**Composite kidney outcome** Patients with CKD and/or T2D, MRA vs placebo: *n* = 8, RR 0.85 (0.82 to 0.88; *p* < 0.001, *I*^2^ = 0%)	High	Low
Tsukamoto et al., 2022 [149]			CKD and T2D	MRA		RR 0.86 (0.77 to 0.95)			
Li et al., 2022 [122]			T2D	Finerenone		HR 0.84 (0.62 to 1.17)			
Ghosal et al., 2023						HR 0.84 (0.77 to 0.92)			
Gu et al., 2024 [108]						HR 0.84 (0.77 to 0.92)			
Nguyen et al., 2023 [132]			CKD and T2D	MRA, non-steroidal		RR 0.84 (0.77 to 0.92)			
Zhang et al., 2022 [169]			CKD	finerenone		RR 0.86 (0.79 to 0.93)			
Chen et al., 2024 [89]				MRA, non-steroidal		RR 0.86 (0.79 to 0.93)			
Chen et al., 2024 [89]	Kidney	eGFR, > 40% reduction	CKD	MRA, non-steroidal	Placebo	RR 0.85 (0.78 to 0.92)	**> 40% eGFR reduction** Patients with T2D and/or (diabetic) kidney disease, MRA vs placebo: *n* = 4, RR 0.85 (0.82 to 0.88; *p* < 0.001, *I*^2^ = 0%)	High	Low
Bao et al., 2022 [84]			CKD and T2D	Finerenone		RR 0.85 (0.78 to 0.93)			
Zheng et al., 2022 [173]			DKD			RR 0.85 (0.78 to 0.93)			
Jyotsna et al., 2023 [116]			CKD and T2D			RR 0.85 (0.80 to 0.90)			
Bao et al., 2022 [84]	Kidney	ESKD	CKD and T2D	Finerenone	Placebo	RR 0.8 (0.65 to 0.99)	**ESKD** Patients with chronic (diabetic) kidney disease, finerenone vs placebo: *n* = 3, RR 0.80 (0.72 to 0.89; *p* < 0.001, *I*^2^ = 0%)	High	Low
Dutta et al., 2022 [95]			DKD			RR 0.79 (0.62 to 1.01)			
Jyotsna et al., 2023 [116]			CKD and T2D			RR 0.80 (0.69 to 0.93)			
Xu et al., 2024 [158]	Kidney	eGFR, > 30% reduction	CKD and T2D	MRA, non-steroidal	Placebo	OR 1.34 (0.83 to 2.18)	**eGFR reduction of kidney failure** Patients with CKD with and without T2D, MRA vs placebo: *n* = 9, OR 0.84 (0.74 to 0.96; *p* = 0.008, *I*^2^ = 68%)	Moderate (inconsistency)	Low
Yasmin et al., 2023 [165]		eGFR, > 40% reduction	CKD	Finerenone		OR 0.82 (0.74 to 0.91)			
Dutta et al., 2022 [95]			DKD			OR 0.83 (0.75 to 0.92)			
Yasmin et al., 2023 [165]		eGFR, > 57% reduction	CKD			OR 0.70 (0.59 to 0.82)			
Yang et al., 2022 [161]			CKD and T2D	MRA, nonselective		OR 1.6 (0.54 to 4.92)			
Dutta et al., 2022 [95]			DKD	Finerenone		OR 0.7 (0.6 to 0.82)			
Yang et al., 2019 [160]		eGFR, reduction	CKD and T2D	Spironolactone		OR 3.3 (1.5 to 9.4)			
Yang et al., 2022 [161]		Kidney failure		MRA, nonselective		OR 1.22 (0.05 to 32.9)			
				MRA, non-steroidal		OR 0.85 (0.71 to 1)			
Xiang et al., 2022 [155]	Mortality	All cause	HFmrEF and HFpEF	MRA	Placebo	OR 0.92 (0.77 to 1.08)	**All-cause mortality** Patients with HF, MRA vs placebo: *n* = 4, OR 0.88 (0.79 to 0.98; *p* = 0.021, *I*^2^ = 9%)	Moderate (inconsistency)	High
Al-Sadawi et al., 2024 [82]			HFpEF			OR 0.63 (0.43 to 0.92)			
Sreenivasan et al., 2022 [144]						OR 0.9 (0.75 to 1.08)			
Sreenivasan et al., 2024 [143]						OR 0.90 (0.75 to 1.08)			
Zhang et al., 2022 [169]	Mortality	All cause	CKD	Finerenone	Placebo	RR 0.9 (0.8 to 1)	**All-cause mortality** Patients with CKD (with or without T2D), MRA vs placebo: *n* = 8, RR 0.89 (0.82 to 0.96; *p* = 0.004, *I*^2^ = 49%)	Moderate (inconsistency)	Low
Lunney et al., 2020 [125]				MRA		RR 0.61 (0.06 to 6.59)			
Bao et al., 2022 [84]			CKD and T2D	Finerenone		RR 0.9 (0.8 to 1.00)			
Tsukamoto et al., 2022 [149]				MRA		RR 0.9 (0.77 to 1.05)			
Nguyen et al., 2023 [132]				MRA, non-steroidal		RR 0.89 (0.8 to 1)			
Dutta et al., 2022 [95]			DKD	Finerenone		RR 0.89 (0.79 to 1)			
Yang et al., 2024 [163]				MRA, non-steroidal		RR 1.12 (0.85 to 1.45)			
Hasegawa et al., 2021 [112]			ESKD requiring dialysis	MRA		RR 0.45 (0.3 to 0.67)			
Hansen et al., 2020 [110]	Mortality	All cause	HF	MRA	Placebo	HR 0.77 (0.68 to 0.88)	**All-cause mortality** Patients with HF, MRA vs placebo: *n* = 9, RR 0.86 (0.81 to 0.90; *p* < 0.001, *I*^2^ = 0%)	Moderate (inconsistency)	Moderate
Zafeiropoulos et al., 2024 [166]			HFmrEF and HFpEF			HR 0.83 (0.68 to 1.02)			
Bonsu et al., 2018 [87]			HFpEF	Eplerenone		RR 1.01 (0.26 to 3.67)			
Martin et al., 2021 [128]				MRA		RR 0.91 (0.78 to 1.06)			
Geng et al., 2023 [51]			CHF			RR 0.82 (0.74 to 0.90)			
Täger et al., 2019 [145]			HFrEF	Spironolactone		RR 1.00 (0.01 to 73.7)			
Bonsu et al., 2018 [87]			HFpEF			RR 0.92 (0.79 to 1.08)			
Faisal et al., 2022 [98]						RR 0.92 (0.79 to 1.08)			
Bonsu et al., 2018 [87]						RR 0.97 (0.69 to 1.35)			
Xiang et al., 2022 [155]			HFpEF and HFmrEF	MRA		HR 0.92 (0.77 to 1.08)			
Zhang et al., 2022 [169]	Mortality	CV	CKD	Finerenone	Placebo	RR 0.88 (0.76 to 1.02)	**CV mortality** Patients with CKD (with or without T2D), finerenone vs placebo: *n* = 5, RR 0.86 (0.82 to 0.91; *p* < 0.001, *I*^2^ = 0%)	High	Low
Li et al., 2022 [122]			T2D			HR 0.88 (0.69 to 1.13)			
Jyotsna et al., 2023 [116]			CKD and T2D			RR 0.86 (0.80 to 0.93)			
Yasmin et al., 2023 [165]			CKD			HR 0.84 (0.74 to 0.95)			
Abdelazeem et al., 2022 [77]			CKD and T2D			RR 0.88 (0.76 to 1.02)			
Geng et al., 2023 [51]	Mortality	CV	CHF	MRA	Placebo	RR 0.80 (0.71 to 0.90)	**CV mortality** Patients with HF, MRA vs placebo: *n* = 7, RR 0.83 (0.79 to 0.88; *p* < 0.001, *I*^2^ = 0%)	Moderate (inconsistency)	Moderate
Oraii et al., 2024 [134]			HF			HR 0.82 (0.76 to 0.88)			
Täger et al., 2019 [145]			HFrEF	Spironolactone		RR 1.06 (0.01 to 90.95)			
Zafeiropoulos et al., 2024 [166]			HFmrEF and HFpEF	MRA		HR 0.74 (0.57 to 0.97)			
Xiang et al., [155]						HR 0.91 (0.73 to 1.12)			
Martin et al., 2021 [128]			HFpEF			RR 0.9 (0.74 to 1.11)			
Faisal et al., [98]				Spironolactone		RR 0.91 (0.74 to 1.11)			
Xiang et al., [155]			HFpEF and HFmrEF	MRA		HR 0.91 (0.73 to 1.12)			
Ghosal et al., 2022 [106]	Various AEs	Any AE	T2D	Finerenone	Placebo	RR 1.00 (0.98 to 1.01)	**Adverse events** Patients with CKD (with or without T2D), MRA vs placebo: *n* = 6, RR 1.00 (0.99 to 1.01; *p* = 1.00, *I*^2^ = 0%)	High	Low
Jyotsna et al., 2023 [116]			CKD and T2D			RR 1.00 (0.99 to 1.01)			
Fu et al., 2021 [104]			CKD			RR 1.00 (0.98 to 1.02)			
Zheng et al., 2022 [173]			DKD			RR 1.00 (0.98 to 1.01)			
Chen et al., 2024 [89]			CKD	MRA, non-steroidal		RR 1.00 (0.99, 1.01)			
Bao et al., 2022 [84]			CKD and T2D	Finerenone		RR 1.00 (0.98 to 1.01)			
Wright et al., 2018 [152]	VARIOUS AES	DISCONTINUATION	HT, first-line therapy	Thiazide	Placebo	RR 3.22 (2.9 to 3.57)	**Discontinuation** Patients with HT, thiazides vs placebo: *n* = 3, RR 3.25 (2.36 to 4.46; *p* < 0.001, *I*^2^ = 94%)	High	Low
Liu et al., 2022 [124]				Thiazide, high-dose	Placebo or no anti-hypertensive treatment	RR 4.48 (3.83 to 5.24)			
Wright et al., 2018 [152]				Thiazide, low-dose		RR 2.38 (2.06 to 2.75)			
Geng et al., 2023 [51]	Various AEs	Gynecomastia	CHF	Eplerenone	Placebo	RR 0.72 (0.32 to 1.61)	**Gynecomastia** Patients with HF, eplerenone or spironolactone vs placebo: *n* = 4, RR 2.50 (0.67 to 9.29; *p* = 0.17, *I*^2^ = 92%)	Very low (inconsistency and imprecision)	Low
Frankenstein et al., 2020 [103]					Placebo or standard medical care	HR 0.77 (0.31 to 1.88)			
Geng et al., 2023 [51]				Spironolactone	placebo	RR 7.48 (4.42 to 12.68)			
Frankenstein et al., 2020 [103]					Placebo or standard medical care	HR 8.44 (3.9 to 18.28)			
Yang et al., 2019 [160]	Various AEs	Any AE	CKD and T2D	Canrenone	Placebo	OR 3.7 (1.1 to 13)	**Adverse events** Patients with CKD and T2D, MRA vs placebo: *n* = 3, OR 1.55 (0.81 to 2.96; *p* = 0.19, *I*^2^ = 75%)	Low (imprecision and inconsistency)	Low
Xu et al., 2024 [158]				MRA, non-steroidal		OR 1.00 (0.92 to 1.10)			
Yang et al., 2019 [160]				Spironolactone		OR 1.8 (1.00 to 3.6)			

*AE* adverse event, *AKI* acute kidney injury, *BP* blood pressure, *CI* confidence interval, *CKD* chronic kidney disease, *CV* cardiovascular, *CVD* cardiovascular disease, *DBP* diastolic blood pressure, *DKD* diabetic kidney disease, *ESKD* end-stage kidney disease, *GRADE* Grading of Recommendations, Assessment, Development and Evaluation, *HF* heart failure, *HFmrEF* heart failure with mid-range ejection fraction, *HFpEF* heart failure with preserved ejection fraction, *HFrEF* heart failure with reduced ejection fraction, *HR* hazard ratio, *HT* hypertension, *MACE* major adverse cardiovascular event, *MD* mean difference, *MI* myocardial infarction, *MRA* mineralocorticoid antagonist, *OR* odds ratio, *RR* risk ratio, *SBP* systolic blood pressure, *SMD* standardized mean difference, *T2D* type 2 diabetes mellitus, *UACR* urinary albumin-to-creatinine ratio, *WMD* weighted mean difference

**Table 3:** Summary of findings table, meta-analyses comparing diuretics with no diuretics

Review first author, year of publication	Outcome category	Specific outcome	Diuretic indication, population	Diuretic A	Diuretic B	Effect (metric, 95% CI)	Meta-analysis pooled data (number of effect estimates, metric, 95% CI, I^2^)	GRADE (reasons for downgrading)	Overall overlap
Singh et al., 2023 [142]	Hospitalization	HF-related	HF	Torasemide	Furosemide	RR 0.61 (0.45 to 0.83)	RR 0.53 (0.41 to 0.69; *p* < 0.001; *I*^2^ = 46%)	High	High
Täger et al., 2019 [145]			HFrEF			RR 0.4 (0.28 to 0.58)			
Teixeira et al., 2024 [146]			HF			RR 0.6 (0.43 to 0.83)			
Shah et al., 2018 [60]	Hospitalization	HF-related	HF	Torasemide	Furosemide	OR 3.03 (2.00 to 4.55)	OR 1.18 (0.68 to 2.04; *p* = 0.552; *I*^2^ = 91%)	Low (imprecision and inconsistency)	Moderate
Abraham et al., 2020 [78]						OR 0.72 (0.51 to 1.03)			
Kido et al., 2019 [119]						OR 0.79 (0.57 to 1.09)			
Miles et al., 2019 [130]						OR 2.04 (1.16 to 3.6)			
Siddiqi et al., 2023 [141]						OR 0.73 (0.54 to 0.99)			
Zhao et al., 2020	Mortality	All cause	HF	Torasemide	Furosemide	OR 0.91 (0.56 to 1.47)	OR 0.96 (0.82 to 1.13; *p* = 0.651; *I*^2^ = 0%)	Moderate (imprecision)	Moderate
Sherif et al., 2019 [70]						OR 0.88 (0.57 to 1.38)			
Abraham et al., 2020 [78]						OR 0.9 (0.58 to 1.41)			
Kido et al., 2019 [119]						OR 1.00 (0.58 to 1.72)			
Miles et al., 2019 [130]						OR 1.12 (0.7 to 1.8)			
Siddiqi et al., 2023 [141]						OR 0.98 (0.75 to 1.29)			

*CI* confidence interval, *GRADE* Grading of Recommendations, Assessment, Development and Evaluation, *HF* heart failure, *HFrEF* heart failure with reduced ejection fraction, *OR* odds ratio, *RR* risk ratio

We performed 33 MAs [30 comparing diuretics (or diuretic (sub) classes] to placebo (Table 2) and 3 comparing torasemide to furosemide (Table 3)). Overall overlap of original RCTs in our MAs was low in 64, moderate in 30, and high in 6% (Tables 2, 3). Ten results for MAs comparing diuretics with placebo, and for one MA comparing torasemide and furosemide had the combination of large statistical significance (*p* < 0.001), high certainty evidence (GRADE classification “high”) and low heterogeneity (*I*^2^ ≤ 50%). For two MAs, we performed sensitivity analyses based on overall RoB classification.

### Mortality

Our meta-analyses (Table 2) show that in persons with HF, MRAs compared to placebo reduced the risk of all-cause mortality [risk ratio (RR) 0.86; 0.81 to 0.90 and odds ratio (OR) 0.88; 0.79 to 0.98, respectively; both moderate certainty]. Similarly, CV mortality risk was lower with MRAs compared to placebo (RR 0.83; 0.79 to 0.88; moderate certainty). The odds of all-cause mortality were comparable between torasemide and furosemide (OR 0.96; 0.82 to 1.13; moderate certainty). In individuals with chronic kidney disease (CKD) and/or type 2 diabetes (T2D), all-cause mortality risk was lower with MRA compared to placebo (RR 0.89; 0.82 to 0.96; moderate certainty); CV mortality risk was lower with finerenone compared to placebo (RR 0.86; 0.82 to 0.91; high certainty).

Our non-pooled data (Supplementary Tables 4 and 5) suggest that diuretics may reduce CV mortality. One review [47] reported CV mortality in older (≥ 65 years old), but not in younger adults (< 65 years old). A Cochrane review showed that thiazides as first-line primary prevention in healthy adults ≥ 60 years old with moderate-to-severe HT may reduce all-cause, CV, cerebrovascular and coronary mortality [44]. A subgroup analysis of one SR showed that in adults with HT, MRA use was associated with a reduced risk of all-cause mortality in older (≥ 65 years), but not in younger individuals [57]. Diuretics or MRAs may lack beneficial effects on all-cause mortality in individuals with CVD. Diuretics or MRAs may have beneficial effect on vascular or HF-related mortality in individuals with a history of CVD. MRAs may reduce CV mortality in adults with end-stage kidney disease (ESKD) or a history of myocardial infarction (MI), but not with HFpEF [HF with preserved ejection fraction (EF)] or CKD and/or T2D. In individuals with HT, MRAs may have beneficial effects on all-cause mortality. MRAs may postpone death in individuals with HF. Spironolactone may have a favorable effect on all-cause mortality in individuals with ESKD requiring dialysis, but in persons with HFpEF, effects were inconsistent. Compared to eplerenone and spironolactone, finerenone may have a beneficial effect on CV mortality.

### Cardiovascular effects

Our meta-analyses (Table 2) show that MRAs (irrespective of indication/population) compared to placebo reduce the odds of new-onset or recurrent atrial fibrillation (OR 0.58; 0.47 to 0.72; moderate certainty). In adults with HF, the risk of developing a composite CV end-point was comparable between MRAs and placebo (moderate certainty). In individuals with HF, the risk of new-onset or recurrent atrial fibrillation was lower with MRAs compared to placebo (RR 0.76; 0.71 to 0.82; high certainty). The effects on systolic and diastolic blood pressure (SBP and DBP, respectively) were comparable for MRAs and placebo (low and moderate certainty, respectively). In adults with HT, CV event risk and SBP were lower with thiazides compared to placebo (RR 0.85; 0.80 to 0.90; high certainty, and SMD − 4.23; − 6.40 to − 2.10; high certainty, respectively). The difference in SBP was clinically relevant [58, 59]. In persons with CKD with and without T2D, major adverse CV event (MACE) risk and SBP were lower with MRAs compared to placebo (RR 0.88; 0.83 to 0.93; high certainty, and SMD − 0.51; − 0.87 to − 0.14; moderate certainty, respectively), but stroke risk was comparable between MRAs and placebo (high certainty). The difference in SBP was not clinically relevant [58, 59]. The risk of MI was lower with finerenone compared to placebo (RR 0.90; 0.81 to 0.99; high certainty).

Our non-pooled data (Supplementary Tables 4 and 5) suggest that diuretics may reduce the risk of composite CV outcomes, CV events, CVD, and stroke. Results for potential age-related differences in stroke risk reduction showed inconsistent results: in one review, the risk was reduced in older, but not in younger (cut-off 65 years) adults [47], whereas in another review, the risk was reduced both in individuals older and younger than 60 years [46]. In persons with ESKD requiring dialysis, there were no age-related differences in BP effects of MRAs [55]. MRAs may have favorable effects on BP regulation, and may reduce the risk of composite CV outcomes, and CVD and CV events. MRAs may have favorable effects on atrial fibrillation, irrespective of age [48]). MRAs may reduce edema in individuals with CKD, but not with HT. Finerenone may reduce the risk of new-onset HT. Thiazide(like) diuretics may have favorable effects on BP regulation in persons with CKD and with uncontrolled HT, and may reduce the risk of MACE, MI, and revascularization and stroke risk. Meta-regression analysis in an SR of RCTs in individuals with HF showed no age-related differences in effects of MRAs on MACE [51]. An individual participant-level data MA showed that there may not be age-dependent effects of thiazides for the prevention of CVD [49]. A subgroup analysis of an SR among individuals with HT living in Sub-Saharan Africa showed that the BP effects of diuretics were independent of age [54]. A Cochrane review showed that thiazides as first-line primary prevention in healthy adults ≥ 60 years old with moderate-to-severe HT may reduce all-cause, CV, and cerebrovascular and coronary morbidity [44]. Data regarding the effects of finerenone on HF-related outcomes in persons with CKD were inconsistent. Low-dose but not high-dose thiazides may reduce the risk of coronary events in persons with HT. Compared to furosemide, torasemide may have favorable effects on HF.

### Hospitalization

Our meta-analyses (Tables 2, 3) show that in persons with HF and in persons with CKD and/or T2D, HF-related hospitalization (HFH) risk was lower with MRAs compared to placebo (RR 0.79; 0.75 to 0.83; high certainty, and RR 0.78; 0.73 to 0.82; high certainty, respectively). The risk, but not the odds of HFH were lower with torasemide compared to furosemide (RR 0.53; 0.41 to 0.69; high certainty, and OR 1.18; 0.68 to 2.04; low certainty). Excluding the data from a review with high RoB [60] did not impact the results (data not shown).

Our non-pooled data (Supplementary Tables 4 and 5) suggest that MRAs may have a beneficial effect on HFH. Results for all-cause and for CVD-related hospitalization were inconsistent. In individuals with T2D or CKD, but not with HFpEF, MRAs may increase the risk of hyperkalemia-related hospitalization. Compared to furosemide, torasemide may have beneficial effects on all-cause and CV-related hospitalization, and on hospital admission duration.

### Kidney effects

Our meta-analyses (Table 2) show that in individuals with CKD and/or T2D and compared to placebo, MRAs reduce the risk of developing a composite kidney outcome (RR 0.85; 0.82 to 0.88; high certainty), reduce the odds of estimated glomerular filtration rate (eGFR) worsening or kidney failure (OR 0.84; 0.74 to 0.96; moderate certainty), reduce the risk of a > 40% eGFR worsening (RR 0.85; 0.82 to 0.88; high certainty), reduce urinary albumin-to-creatinine ratio (UACR) (SMD − 1.31; − 1.84 to − 0.77; high certainty), and reduce eGFR (SMD − 0.40; − 0.69 to − 0.11; high certainty). The reductions in eGFR and UACR are not clinically relevant [61]. Acute kidney injury (AKI) risk was comparable with MRAs and placebo (moderate certainty). In individuals with chronic (diabetic) kidney disease, the risk of developing ESKD was lower with finerenone compared to placebo (RR 0.80; 0.72 to 0.89; high certainty).

Our non-pooled data (Supplementary Table 4) suggest that in persons with kidney disease, MRAs may lower UACR, 24 h albuminuria, and proteinuria. MRAs may have a beneficial effect on kidney function (eGFR, CKD progression, and composite kidney outcome) in individuals with CKD and/or T2D, but effects on ESKD and kidney failure are inconsistent. Thiazides may have a beneficial effect on kidney stone-related morbidity in individuals with idiopathic hypercalciuria or a history of kidney stones. Thiazide(like) diuretics may reduce GFR in persons with advanced CKD. In patients with diabetic kidney disease (DKD), eplerenone may be more effective in reducing microalbuminuria.

### Effects on biochemistry

Our meta-analyses (Table 2) show that in persons with HF, MRAs compared to placebo increase the risk of hyperkalemia (RR 2.09; 1.87 to 2.33; high certainty, and OR 1.82; 1.30 to 2.55; moderate certainty). Sensitivity analysis showed that 2/3 of the estimated ORs were from a review with high RoB. In persons with CKD (with and without T2D), MRAs compared to placebo increase the risk of hyperkalemia (RR 2.31; 2.07 to 2.58; high certainty, and OR 2.26; 1.96 to 2.61; moderate certainty, respectively).

Our non-pooled data (Supplementary Table 4) suggest that thiazides may lower serum potassium in individuals with HT. MRAs may increase serum potassium in adults with CV disease [CKD, T2D, DKD, and HFmrEF (HF with mid-range EF) or HFpEF], but not with ESKD, and lower the risk of hypokalemia in persons with HF. The risk of hyperkalemia may be increased by MRAs irrespective of indication. Spironolactone may increase serum calcium. Nonsteroidal MRAs may increase hyponatremia risk in persons with CKD and/or T2D. Thiazides may increase (fasting) serum glucose. New-onset diabetes risk was comparable with diuretics and placebo, irrespective of age (younger vs older than 65 years) [45].

### Effects on blood heart biomarkers

Our non-pooled data (Supplementary Tables 4 and 5) suggest that in patients with HFmrEF or HFpEF, spironolactone may reduce serum brain natriuretic peptide (BNP) and fibrosis marker PICP (procollagen type I C-terminal propeptide), and in patients with HFpEF, may reduce fibrosis marker PIIINR (amino-terminal peptide of procollagen type-III). Compared to furosemide, torasemide may have beneficial effects on BNP and BNP/NT-proBNP (N-terminal pro b-type natriuretic peptide) ratio.

### Effects on heart ultrasonography variables

Our non-pooled data (Supplementary Tables 4 and 5) suggest that MRAs may have favorable effects on augmentation index, flow-mediated dilation (FMD), and pulse wave velocity (PWV), probably irrespective of age [53]. In persons with ESKD requiring dialysis, MRAs may reduce left ventricle mass (LVM). Spironolactone may reduce left-ventricular mass index (LVMI), but effects on left-ventricular ejection fraction (LVEF) are inconsistent. MRAs may have a favorable effect on LVEF in persons after MI, and a favorable effect on E', E/e', left atrial volume index (LAVI), and left-ventricular end-diastolic diameter (LVEDD) in individuals with HFpEF. Spironolactone may have a favorable effect on LVEDD in individuals with HFmrEF or HFpEF, and on E/e’ in persons with HFpEF, irrespective of age (70 year cut-off) [52]. Effects on left-ventricular end-diastolic volume (LVEDV) may be favorable with torasemide compared to furosemide.

### Effects on functional performance

Our non-pooled data (Supplementary Table 4) suggest that the 6 min walking distance (6MWD) may be increased by spironolactone in individuals with HFmrEF or HFpEF; in persons with HFpEF, 6MWD may be comparable or reduced by spironolactone/MRAs.

### Effects on cognitive performance

Our non-pooled data (Supplementary Table 4) suggest that diuretics may decrease the risk of a new dementia diagnosis in studies with a follow-up duration of ≥ 1, but not ≥ 5 years.

### Adverse events and discontinuation

Our meta-analyses (Table 2) show that in persons with CKD with or without T2D, the risk and odds of AEs are comparable with MRAs and placebo (high and low certainty, respectively). In individuals with HF, the risk of developing gynecomastia was comparable for eplerenone or spironolactone compared to placebo (very low certainty). In adults with HT, the risk of discontinuation was higher with thiazides compared to placebo (RR 3.25; 2.36 to 4.46; high certainty).

Our non-pooled data (Supplementary Tables 4 and 5) suggest that diuretics may increase the risk of AEs. In older (≥ 65 years), but not in younger adults, the risk of AEs may be increased [47]. In persons with uncontrolled HT, addition of hydrochlorothiazide to ARB (angiotensin receptor blocker) therapy may increase the risk of drug-related AEs; the risk of any AEs was increased with high-dose, but not with low-dose hydrochlorothiazide. MRAs may increase the risk of hyperkalemia-related AEs in persons with HT. Finerenone may increase the risk of drug-related AEs, but reduce the risk of HF-related AEs and serious AEs in persons with CKD and/or T2D. Steroidal MRAs (especially spironolactone), but not non-steroidal MRAs (finerenone) may increase the risk of gynecomastia and breast pain. In patients with calcium oxalate stones, AE risk may be increased by thiazides. MRAs or spironolactone may increase the risk of discontinuation in individuals with CKD and/or T2D, ESKD or HFrEF. Nonsteroidal MRAs may reduce the risk of severe hypoglycemia in individuals with T2D. Finerenone may increase the risk of urinary tract malignancy, but not other neoplasms in adults with CKD and/or T2D. MRAs may increase the risk of hypotension in individuals with CKD, but not with HF, HT, or ESKD requiring dialysis. Thiazide(like) diuretics may have a neutral effect on fracture risk, irrespective of age [50]. Finerenone may have a more favorable AE profile than spironolactone (lower risk of AEs and hyperkalemia). The risk of gynecomastia and of hyperkalemia may be larger with spironolactone compared to eplerenone.

### Quality of life

Our non-pooled data (Supplementary Table 4) suggest that MRAs or spironolactone may have a neutral effect on QoL.

## Discussion

The findings of this umbrella review suggest both potential benefits and harms of chronic diuretic use in adults. We found beneficial effects for finerenone, which likely reduces the risk of CV mortality and ESKD in individuals with CKD and/or T2D. Thiazides probably reduce CV events in individuals with HT, and MRAs likely reduce HF-related hospitalization in individuals with HF and atrial fibrillation risk in individuals with CVD. Additionally, MRAs may reduce HFH, MACE, > 40% eGFR decrease, and composite kidney outcomes in individuals with CKD and/or T2D. Regarding potential harms of diuretic use, we found that MRAs increase the risk of hyperkalemia in individuals with HF.

Mean age in the SRs we analyzed was only 62 years, which is relatively young compared to most adults with CVD (> 70% of adults has developed CVD by age 70 [3], and 80% of HF patients are older than 65) [62, 63]. This younger mean age aligns with typical demographics in RCTs in the CV field, where participant ages generally range from 61 to 64 years. Yet, this is younger than the average age of patients in real-world clinical settings as indicated by epidemiological studies (e.g., the mean age for patients with acute coronary syndrome is generally between 66 and 70 years [64]). The potential clinical relevance of these age-related differences is underscored by our findings, which suggest that diuretics and MRAs may have differential effects across age groups. For instance, we observed potential benefits (e.g., reduced CV mortality) in older, but not in younger adults. Furthermore, in adults with HT, MRA use was associated with a reduced risk of all-cause mortality in older, but not younger individuals. This may be attributed to age-related decreases in plasma aldosterone levels [56, 65]. Additionally, we found that the risk of AEs associated with diuretic use was higher in older adults (≥ 65 years), but not in younger adults. These findings suggest that age-related differences in benefits and risks of diuretics must be considered. Although the population we analyzed is relatively young compared to the real-world patient population, our results remain highly relevant for older patients. It is important to note that individuals with CVD, as studied in our umbrella review, are biologically older than their peers of the same chronological age without CVD [66, 67]. Not only is the biological age of individuals with CVD (as studied in our umbrella review) typically higher, but their clinical characteristics also align with those of older individuals: patients with CVD generally experience a high prevalence of multiple geriatric conditions such as multimorbidity (≥ 2 chronic conditions), polypharmacy (≥ 5 medications), cognitive impairment, vision and hearing impairments, urinary incontinence, functional decline, frailty, and sarcopenia [68, 69]. Thus, despite the younger chronological age of our study population, our findings are applicable to older, more biologically frail patients with CVD. Another notable mismatch is the paucity of data on loop diuretics despite their widespread use in HF [> 80% HF patients are on loop diuretics [70], yet only 3 of 319 (0.9%) of the placebo-controlled effect estimations we included focused on loop diuretics]. Furthermore, the vast majority (97%) of the diuretic efficacy outcome estimates in our analyses were on objective, quantifiable outcomes such as mortality, hospitalization, or MACE. Although typical priorities for older adults include physical and cognitive function, symptom control, reduced therapy burden, HR-QoL, maintenance of independence, and overall well-being [6], less than 3% of the efficacy outcomes we included addressed these key factors that are highly relevant to older individuals. These discrepancies reflect broader issues in CV research, where older adults, particularly those with multimorbidity, are often underrepresented in clinical trials [71, 72]. Unfortunately, we were not able to perform subgroup or meta-regression analyses based on age and multimorbidity/frailty status.

A key strength of our review lies in its broad scope regarding settings, health outcomes, and diuretic therapy we deliberately adopted to fit the needs encountered in clinical practice. Additional strengths of our review include its rigorous methodology, including the use of the GRADE framework, RoB assessment, and performing sensitivity and overlap analyses. Furthermore, we evaluated the effects of diuretics on a range of biomarkers, such as NT-proBNP. Although biomarkers cannot replace clinical outcomes, some may serve as useful indicators of safety [46, 47]. Also, alongside statistical significance, we consider clinical relevance by incorporating the concept of minimal clinically important difference into our conclusions [48]. Our results may serve as a source of reference for clinicians, and will be used by our group to inform an evidence-based clinical practice guideline on diuretic deprescribing, aiming to support healthcare providers in discussions about the long-term use of diuretics and in making individualized treatment decisions in routine clinical practice.

However, our methodology does have limitations. First, the broad PICOS approach may reduce the generalizability of our findings. By focusing solely on published MA data, we excluded evidence from individual cohort studies or RCTs that have not been previously aggregated in MAs. Additionally, while we assessed the quality of the MAs, we did not evaluate the quality of all the primary studies included in these MAs, as this was beyond the scope of this review and would have been impractical given the large volume of studies involved. Finally, unlike traditional meta-analyses, umbrella reviews do not permit subgroup or sensitivity analyses due to several methodological and reporting limitations [73, 74]. As a result, we were unable to quantify potential differential effects of chronic diuretic use in specific subgroups such as the oldest old compared to younger counterparts or those residing in long-term care facilities (e.g., nursing homes) versus non-institutionalized individuals.

Further research is needed to establish causal relationships between chronic diuretic use and health outcomes, especially in older adults. Future studies should focus on real-world populations with multimorbidity and frailty [75], considering patient-centered outcomes like QoL, physical function, and independence [1, 63, 71]. Innovative methodologies on trials with large, diverse populations could help refine treatment decisions and quantify benefits and harms, while considering patient preferences [76]. This additional evidence will be critical for guiding clinical decisions on the initiation, continuation, or cessation of diuretic therapy, weighing the benefits, harms, and burdens in light of individual health priorities.

## Conclusions

This umbrella review provides a comprehensive synthesis of the associations between diuretics and various health outcomes. Our findings suggest that certain diuretics, or diuretic (sub)classes, confer significant benefits for key clinical outcomes, including CV mortality, HFH, and CKD in specific populations with CVD. However, our results also underscore the potential risks associated with chronic diuretic use, such as an elevated risk of hyperkalemia in individuals with HF.

Age-related differences in AE risks were present, with older adults (≥ 65 years) exhibiting higher risks, while younger populations did not demonstrate similar concerns. Additionally, our analysis highlights a critical gap between the extensive body of existing data and the clinical relevance of this evidence for older individuals with CVD as encountered in clinical practice. This gap reflects broader challenges in CV research, where older adults—especially those with multimorbidity—are frequently underrepresented in clinical trials. Although older adults typically prioritize outcomes related to physical and cognitive function, symptom management, reduced treatment burden, health-related QoL, independence, and overall well-being, these outcomes represented less than 3% of all efficacy outcomes we reviewed.

To draw more definitive conclusions regarding the effects of chronic diuretic use and to refine clinical diuretic prescribing decision-making, further research is crucial, particularly for older adults with multimorbidity.

## Supplementary Information

Below is the link to the electronic supplementary material.

Supplementary file1 (DOCX 33 KB)

Supplementary file2 (DOCX 25 KB)

Supplementary file3 (DOCX 56 KB)

Supplementary file4 (DOCX 135 KB)

Supplementary file5 (DOCX 70 KB)

## Data Availability

Data are available upon request.

## References

[1] Filbey L, Zhu JW, D’Angelo F, Thabane L, Khan MS, Lewis E et al (2023) Improving representativeness in trials: a call to action from the Global Cardiovascular Clinical Trialists Forum. Eur Heart J 44(11):921–930. 10.1093/eurheartj/ehac810 36702610 10.1093/eurheartj/ehac810 PMC10226751

[2] Coll PP, Roche V, Olsen JS, Voit JH, Bowen E, Kumar M (2020) The prevention of cardiovascular disease in older adults. J Am Geriatr Soc 68(5):1098–1106. 10.1111/jgs.16353 32031247 10.1111/jgs.16353

[3] Forman DE, Maurer MS, Boyd C, Brindis R, Salive ME, Home FM et al (2018) Multimorbidity in older adults with cardiovascular disease. J Am Coll Cardiol 71(19):2149–2161. 10.1016/j.jacc.2018.03.022 29747836 10.1016/j.jacc.2018.03.022 PMC6028235

[4] Ellison DH, Felker GM (2018) Diuretic treatment in heart failure. New Engl J Med 378(7):684–685 29443667 10.1056/NEJMc1716477

[5] Reinhart M, Puil L, Salzwedel DM, Wright JM (2023) First-line diuretics versus other classes of anti-hypertensive drugs for hypertension. Cochrane Database Syst Rev 7:ARTN CD008161. 10.1002/14651858.CD008161.pub3 10.1002/14651858.CD008161.pub3 PMC10339786 37439548

[6] Savarese G, Becher PM, Lund LH, Seferovic P, Rosano GMC, Coats AJS (2022) Global burden of heart failure: a comprehensive and updated review of epidemiology. Cardiovasc Res 118(17):3272–3287. 10.1093/cvr/cvac013 10.1093/cvr/cvac013 35150240

[7] Bourgeois FT, Olson KL, Tse T, Ioannidis JPA, Mandl KD (2016) Prevalence and characteristics of interventional trials conducted exclusively in elderly persons: a cross-sectional analysis of registered clinical trials. PLoS ONE 11(5):ARTN e0155948. 10.1371/journal.pone.0155948 10.1371/journal.pone.0155948 PMC4873036 27196289

[8] Simonavicius J, Knackstedt C, Brunner-La Rocca HP (2019) Loop diuretics in chronic heart failure: how to manage congestion? Heart Fail Rev 24(1):17–30. 10.1007/s10741-018-9735-7 30194516 10.1007/s10741-018-9735-7

[9] Ravioli S, Bahmad S, Funk GC, Schwarz C, Exadaktylos A, Lindner G (2021) Risk of electrolyte disorders, syncope, and falls in patients taking thiazide diuretics: results of a cross-sectional study. Am J Med 134(9):1148–1154. 10.1016/j.amjmed.2021.04.007 33974908 10.1016/j.amjmed.2021.04.007

[10] Barber J, McKeever TM, McDowell SE, Clayton JA, Ferner RE, Gordon RD et al (2015) A systematic review and meta-analysis of thiazide-induced hyponatraemia: time to reconsider electrolyte monitoring regimens after thiazide initiation? Br J Clin Pharmacol 79(4):566–577. 10.1111/bcp.12499 25139696 10.1111/bcp.12499 PMC4386942

[11] Ter Maaten JM, Martens P, Damman K, Dickstein K, Ponikowski P, Lang CC et al (2020) Higher doses of loop diuretics limit uptitration of angiotensin-converting enzyme inhibitors in patients with heart failure and reduced ejection fraction. Clin Res Cardiol 109(8):1048–1059. 10.1007/s00392-020-01598-w 32002631 10.1007/s00392-020-01598-w PMC7375987

[12] Mullens W, Verbrugge FH, Nijst P, Tang WHW (2017) Renal sodium avidity in heart failure: from pathophysiology to treatment strategies. Eur Heart J 38(24):1872–1882. 10.1093/eurheartj/ehx035 28329085 10.1093/eurheartj/ehx035

[13] Tomasoni D, Vishram-Nielsen JKK, Pagnesi M, Adamo M, Lombardi CM, Gustafsson F et al (2022) Advanced heart failure: guideline-directed medical therapy, diuretics, inotropes, and palliative care. ESC Heart Fail 9(3):1507–1523. 10.1002/ehf2.13859 35352499 10.1002/ehf2.13859 PMC9065830

[14] Kapelios CJ, Laroche C, Crespo-Leiro MG, Anker SD, Coats AJS, Díaz-Molina B et al (2020) Association between loop diuretic dose changes and outcomes in chronic heart failure: observations from the ESC-EORP Heart Failure Long-Term Registry. Eur J Heart Fail 22(8):1424–1437. 10.1002/ejhf.1796 32237110 10.1002/ejhf.1796

[15] Okoye C, Mazzarone T, Cargiolli C, Guarino D (2023) Discontinuation of loop diuretics in older patients with chronic stable heart failure: a narrative review. Drug Aging 40(11):981–990. 10.1007/s40266-023-01061-1 10.1007/s40266-023-01061-1 PMC10600299 37620655

[16] Sztramko R, Chau V, Wong R (2011) Adverse drug events and associated factors in heart failure therapy among the very elderly. Can Geriatr J 14(4):79–92. 10.5770/cgj.v14i4.19 23251319 10.5770/cgj.v14i4.19 PMC3516232

[17] Kurczewska-Michalak M, Lewek P, Jankowska-Polanska B, Giardini A, Granata N, Maffoni M et al (2021) Polypharmacy management in the older adults: a scoping review of available interventions. Front Pharmacol 12:ARTN 734045. 10.3389/fphar.2021.734045 10.3389/fphar.2021.734045 PMC8661120 34899294

[18] Page RL, Linnebur SA, Bryant LL, Ruscin JM (2010) Inappropriate prescribing in the hospitalized elderly patient: defining the problem, evaluation tools, and possible solutions. Clin Interv Aging 5:75–87 20396637 10.2147/cia.s9564 PMC2854054

[19] Stewart D, Mair A, Wilson M, Kardas P, Lewek P, Alonso A et al (2017) Guidance to manage inappropriate polypharmacy in older people: systematic review and future developments. Expert Opin Drug Saf 16(2):203–213. 10.1080/14740338.2017.1265503 27885844 10.1080/14740338.2017.1265503

[20] Fosnight S, Soric MM, Smearman J, Graves E, Vazquez M, Herrington Z et al (2024) Investigation into potentially inappropriate prescribing patterns of loop diuretics in a nationally representative outpatient population. Am J Ther 31(4):e347–e355. 10.1097/Mjt.0000000000001644 37820082 10.1097/MJT.0000000000001644

[21] Damoiseaux-Volman BA, Raven K, Sent D, Medlock S, Romijn JA, Abu-Hanna A et al (2022) Potentially inappropriate medications and their effect on falls during hospital admission. Age Ageing 51(1):ARTN afab205. 10.1093/ageing/afab205 10.1093/ageing/afab205 PMC8753037 34673915

[22] Huang CH, Umegaki H, Watanabe Y, Kamitani H, Asai A, Kanda S et al (2019) Potentially inappropriate medications according to STOPP-J criteria and risks of hospitalization and mortality in elderly patients receiving home-based medical services. PLoS ONE 14(2):ARTN e0211947. 10.1371/journal.pone.0211947 10.1371/journal.pone.0211947 PMC6368320 30735544

[23] O’Mahony D, O’Sullivan D, Byrne S, O’Connor MN, Ryan C, Gallagher P (2018) STOPP/START criteria for potentially inappropriate prescribing in older people: version 2 (vol 44, pg 213, 2015). Age Ageing 47(3):489. 10.1093/ageing/afx178 10.1093/ageing/afx178 PMC5920325 29182733

[24] Wehling M (2013) Morbus diureticus in the elderly: epidemic overuse of a widely applied group of drugs. J Am Med Dir Assoc 14(6):437–442. 10.1016/j.jamda.2013.02.002 23510827 10.1016/j.jamda.2013.02.002

[25] Carmona C, Crutwell J, Burnham M, Polak L, Comm G (2021) Shared decision-making: summary of NICE guidance. BMJ-Br Med J. 373:ARTN n1430. 10.1136/bmj.n1430 10.1136/bmj.n1430 34140279

[26] Ndai A, Al Bahou J, Morris E, Wang HM, Marcum Z, Hung A et al (2024) Mapping potentially inappropriate medications in older adults using the Anatomical Therapeutic Chemical (ATC) classification system. J Am Geriatr Soc 72(1):126–138. 10.1111/jgs.18681 38124261 10.1111/jgs.18681

[27] Scott IA, Hilmer SN, Reeve E, Potter K, Le Couteur D, Rigby D et al (2015) Reducing inappropriate polypharmacy the process of deprescribing. JAMA Intern Med 175(5):827–834. 10.1001/jamainternmed.2015.0324 25798731 10.1001/jamainternmed.2015.0324

[28] van Poelgeest E, Seppala L, Bahat G, Ilhan B, Mair A, van Marum R et al (2023) Optimizing pharmacotherapy and deprescribing strategies in older adults living with multimorbidity and polypharmacy: EuGMS SIG on pharmacology position paper. Eur Geriatr Med 14(6):1195–1209. 10.1007/s41999-023-00872-0 37812379 10.1007/s41999-023-00872-0 PMC10754739

[29] van Poelgeest E, Paoletti L, Özkök S, Pinar E, Bahat G, Vuong V et al (2024) The effects of diuretic deprescribing in adult patients: a systematic review to inform an evidence-based diuretic deprescribing guideline. Br J Clin Pharmacol. 10.1111/bcp.16189 39117602 10.1111/bcp.16189 PMC11671325

[30] Cuthbert JJ, Clark AL (2024) Diuretic treatment in patients with heart failure: current evidence and future directions—Part I: loop diuretics. Curr Heart Fail Rep 21(2):101–114. 10.1007/s11897-024-00643-3 38240883 10.1007/s11897-024-00643-3 PMC10924023

[31] Vidonscky Luthold R, Jungo KT, Weir KR, Adler L, Asenova R, Ares-Blanco S et al (2025) Older adults’ attitudes toward deprescribing in 14 countries. JAMA Netw Open 8(2):e2457498. 10.1001/jamanetworkopen.2024.57498 39928337 10.1001/jamanetworkopen.2024.57498 PMC11811803

[32] Wilcox CS, Pourafshar N, Han KRA, Shah SZN, Sussman RD, Testani J et al (2024) Bladder symptoms provoked by short, rapid-acting loop diuretics: a frequent but often overlooked problem. Am J Hypertens 38(2):100–103. 10.1093/ajh/hpae139 10.1093/ajh/hpae139 39485998

[33] Cumpston M, Li T, Page MJ, Chandler J, Welch VA, Higgins JP et al (2019) Updated guidance for trusted systematic reviews: a new edition of the Cochrane Handbook for Systematic Reviews of Interventions. Cochrane Database Syst Rev 10(10):ED000142. 10.1002/14651858.ED000142 31643080 10.1002/14651858.ED000142 PMC10284251

[34] Gates M, Gates A, Pieper D, Fernandes RM, Tricco AC, Moher D et al (2022) Reporting guideline for overviews of reviews of healthcare interventions: development of the PRIOR statement. BMJ-Br Med J. 378:ARTN e070849. 10.1136/bmj-2022-070849 10.1136/bmj-2022-070849 PMC9361065 35944924

[35] DerSimonian R, Laird N (2015) Meta-analysis in clinical trials revisited. Contemp Clin Trials 45:139–145. 10.1016/j.cct.2015.09.002 26343745 10.1016/j.cct.2015.09.002 PMC4639420

[36] Lau J, Ioannidis JPA, Schmid CH (1997) Quantitative synthesis in systematic reviews. Ann Intern Med 127(9):820–826. 10.7326/0003-4819-127-9-199711010-00008 9382404 10.7326/0003-4819-127-9-199711010-00008

[37] Higgins JPT, Thompson SG (2002) Quantifying heterogeneity in a meta-analysis. Stat Med 21(11):1539–1558. 10.1002/sim.1186 12111919 10.1002/sim.1186

[38] Higgins JPT (2008) Commentary: Heterogeneity in meta-analysis should be expected and appropriately quantified. Int J Epidemiol 37(5):1158–1160. 10.1093/ije/dyn204 18832388 10.1093/ije/dyn204

[39] Higgins JPT, Thompson SG, Spiegelhalter DJ (2009) A re-evaluation of random-effects meta-analysis. J Roy Stat Soc Ser A-Stat Soc 172:137–159. 10.1111/j.1467-985X.2008.00552.x 10.1111/j.1467-985X.2008.00552.x PMC2667312 19381330

[40] Hennessy EA, Johnson BT (2020) Examining overlap of included studies in meta-reviews: guidance for using the corrected covered area index. Res Synth Methods 11(1):134–145. 10.1002/jrsm.1390 31823513 10.1002/jrsm.1390 PMC8555740

[41] Kirvalidze M, Abbadi A, Dahlberg L, Sacco LB, Calderón-Larrañaga A, Morin L (2023) Estimating pairwise overlap in umbrella reviews: considerations for using the corrected covered area (CCA) index methodology. Res Synth Methods 14(5):764–767. 10.1002/jrsm.1658 37501239 10.1002/jrsm.1658

[42] Pérez-Bracchiglione J, Meza N, Bangdiwala SI, de Guzmán EN, Urrútia G, Bonfill X et al (2022) Graphical representation of overlap for OVErviews: GROOVE tool. Res Synth Methods 13(3):381–388. 10.1002/jrsm.1557 35278030 10.1002/jrsm.1557

[43] Guyatt GH, Oxman AD, Vist GE, Kunz R, Falck-Ytter Y, Alonso-Coello P et al (2008) GRADE: an emerging consensus on rating quality of evidence and strength of recommendations. Br Med J 336(7650):924–926. 10.1136/bmj.39489.470347.ad 18436948 10.1136/bmj.39489.470347.AD PMC2335261

[44] Musini VM, Tejani AM, Bassett K, Puil L, Wright JM (2019) Pharmacotherapy for hypertension in adults 60 years or older. Cochrane Database Syst Rev 6:CD000028. 10.1002/14651858.cd000028.pub3 31167038 10.1002/14651858.CD000028.pub3 PMC6550717

[45] Zhang J, Tong A, Dai Y, Niu J, Yu F, Xu F (2019) Comparative risk of new-onset diabetes mellitus for anti-hypertensive drugs in elderly: a Bayesian network meta-analysis. J Clin Hypertens (Greenwich) 21(8):1082–1090. 10.1111/jch.13598 31241860 10.1111/jch.13598 PMC8030293

[46] Zhong XL, Dong Y, Xu W, Huang YY, Wang HF, Zhang TS et al (2021) Role of blood pressure management in stroke prevention: a systematic review and network meta-analysis of 93 randomized controlled trials. J Stroke 23(1):1–11. 10.5853/jos.2020.02698 33600699 10.5853/jos.2020.02698 PMC7900391

[47] Thomopoulos C, Parati G, Zanchetti A (2018) Effects of blood pressure-lowering treatment on cardiovascular outcomes and mortality: 14—effects of different classes of anti-hypertensive drugs in older and younger patients: overview and meta-analysis. J Hypertens 36(8):1637–1647. 10.1097/hjh.0000000000001777 29847487 10.1097/HJH.0000000000001777

[48] Alexandre J, Dolladille C, Douesnel L, Font J, Dabrowski R, Shavit L et al (2019) Effects of mineralocorticoid receptor antagonists on atrial fibrillation occurrence: a systematic review, meta-analysis, and meta-regression to identify modifying factors. J Am Heart Assoc. 10.1161/jaha.119.013267 31711383 10.1161/JAHA.119.013267 PMC6915291

[49] Bidel Z, Nazarzadeh M, Canoy D, Copland E, Gerdts E, Woodward M et al (2023) Sex-specific effects of blood pressure lowering pharmacotherapy for the prevention of cardiovascular disease: an individual participant-level data meta-analysis. Hypertension 80(11):2293–2302. 10.1161/hypertensionaha.123.21496 37485657 10.1161/HYPERTENSIONAHA.123.21496

[50] Desbiens LC, Khelifi N, Wang YP, Lavigne F, Beaulieu V, Sidibe A et al (2022) Thiazide diuretics and fracture risk: a systematic review and meta-analysis of randomized clinical trials. JBMR Plus 6(11):e10683. 10.1002/jbm4.10683 36398110 10.1002/jbm4.10683 PMC9664541

[51] Geng C, Mao YC, Qi SF, Song K, Wang HF, Zhang ZY et al (2023) Mineralocorticoid receptor antagonists for chronic heart failure: a meta-analysis focusing on the number needed to treat. Front Cardiovasc Med 10:1236008. 10.3389/fcvm.2023.1236008 38028498 10.3389/fcvm.2023.1236008 PMC10657990

[52] Li S, Zhang X, Dong M, Gong S, Shang Z, Jia X et al (2018) Effects of spironolactone in heart failure with preserved ejection fraction: a meta-analysis of randomized controlled trials. Medicine (Baltimore) 97(35):e11942. 10.1097/md.0000000000011942 30170387 10.1097/MD.0000000000011942 PMC6392615

[53] Sakima A, Arima H, Matayoshi T, Ishida A, Ohya Y (2021) Effect of mineralocorticoid receptor blockade on arterial stiffness and endothelial function: a meta-analysis of randomized trials. Hypertension 77(3):929–937. 10.1161/hypertensionaha.120.16397 33461316 10.1161/HYPERTENSIONAHA.120.16397

[54] Seeley A, Prynn J, Perera R, Street R, Davis D, Etyang AO (2020) Pharmacotherapy for hypertension in Sub-Saharan Africa: a systematic review and network meta-analysis. BMC Med 18(1):75. 10.1186/s12916-020-01530-z 32216794 10.1186/s12916-020-01530-z PMC7099775

[55] Shaman AM, Smyth B, Arnott C, Palmer SC, Mihailidou AS, Jardine MJ et al (2020) Comparative efficacy and safety of BP-lowering pharmacotherapy in patients undergoing maintenance dialysis: a network meta-analysis of randomized, controlled trials. Clin J Am Soc Nephrol 15(8):1129–1138. 10.2215/cjn.12201019 32675281 10.2215/CJN.12201019 PMC7409758

[56] Yuan CY, Gao YC, Lin Y, Liu L, Shen XG, Zou WL et al (2024) Effects of mineralocorticoid receptor antagonists for chronic kidney disease: a systemic review and meta-analysis. Am J Nephrol 55(1):1–17. 10.1159/000534366 37793348 10.1159/000534366

[57] Yi X, Yang S, Yang J, Chen X, Zhang A, Zeng Q et al (2024) Renin-angiotensin-aldosterone system modulators in adults with hypertension: a network meta-analysis of randomized controlled trials. Drugs 23:23. 10.1007/s40265-024-02092-7 10.1007/s40265-024-02092-7 39312177

[58] Hardy ST, Loehr LR, Butler KR, Chakladar S, Chang PP, Folsom AR et al (2015) Reducing the blood pressure-related burden of cardiovascular disease: impact of achievable improvements in blood pressure prevention and control. J Am Heart Assoc 4(10):ARTN e002276. 10.1161/JAHA.115.002276 10.1161/JAHA.115.002276 PMC4845128 26508742

[59] Stamler J, Rose G, Stamler R, Elliott P, Dyer A, Marmot M (1989) Intersalt study findings—public-health and medical-care implications. Hypertension 14(5):570–577. 10.1161/01.Hyp.14.5.570 2807518 10.1161/01.hyp.14.5.570

[60] Shah P, Patel H, Mithawala P, Doshi R (2018) Torsemide versus furosemide in heart failure patients: a meta-analysis of randomized controlled trials. Eur J Intern Med 57:e38–e40. 10.1016/j.ejim.2018.08.015 30177487 10.1016/j.ejim.2018.08.015

[61] Levin A, Ahmed SB, Carrero JJ, Foster B, Francis A, Hall RK et al (2024) Executive summary of the KDIGO 2024 clinical practice guideline for the evaluation and management of chronic kidney disease: known knowns and known unknowns. Kidney Int 105(4):684–701. 10.1016/j.kint.2023.10.016 38519239 10.1016/j.kint.2023.10.016

[62] Cherubini A, Oristrell J, Pla X, Ruggiero C, Ferretti R, Diestre G et al (2011) The persistent exclusion of older patients from ongoing clinical trials regarding heart failure. Arch Intern Med 171(6):550–556 21444844 10.1001/archinternmed.2011.31

[63] Bowling CB, Whitson HE, Johnson TM (2019) The preliminary development of a framework to support inclusion of older adults in research. J Am Geriatr Soc 67(2):342–346. 10.1111/jgs.15785 30693952 10.1111/jgs.15785 PMC6532768

[64] Tahhan AS, Vaduganathan M, Greene SJ, Alrohaibani A, Raad M, Gafeer M et al (2020) Enrollment of older patients, women, and racial/ethnic minority groups in contemporary acute coronary syndrome clinical trials a systematic review. JAMA Cardiol 5(6):714–722. 10.1001/jamacardio.2020.0359 32211813 10.1001/jamacardio.2020.0359

[65] Nanba K, Vaidya A, Rainey WE (2018) Aging and adrenal aldosterone production. Hypertension 71(2):218–223. 10.1161/Hypertensionaha.117.10391 29229745 10.1161/HYPERTENSIONAHA.117.10391 PMC5839673

[66] Hamczyk MR, Nevado RM, Barettino A, Fuster V, Andrés V (2020) Biological versus chronological aging. Focus seminar. J Am Coll Cardiol 75(8):919–930. 10.1016/j.jacc.2019.11.062 32130928 10.1016/j.jacc.2019.11.062

[67] Zmora R, Schreiner PJ, Appiah D, Lloyd-Jones DM, Rana JS, Lewis CE (2017) Clinical risk factors related to biological versus chronological heart age: the coronary artery risk development in young adults (CARDIA) study. In: Circulation, vol 135. Lippincott Williams & Wilkins, Philadelphia

[68] Aidoud A, Gana W, Poitau F, Debacq C, Leroy V, Nkodo JA et al (2023) High prevalence of geriatric conditions among older adults with cardiovascular disease. J Am Heart Assoc 12(2):ARTN e026850. 10.1161/JAHA.122.026850 10.1161/JAHA.122.026850 PMC9939057 36628962

[69] Tom-Ayegunle K, Tcheugui JE, Oshozimhede IE, Orkaby A, Kwak MJ, Goyal P et al (2024) The association of geriatric syndromes with cardiovascular disease: insights from ARIC visit 5 on cardiovascular risks in older adults. Circulation. 10.1161/circ.150.suppl_1.4146141

[70] Sherif NA, Morra ME, Thanh LV, Elsayed GG, Elkady AH, Elshafay A et al (2020) Torasemide versus furosemide in treatment of heart failure: a systematic review and meta-analysis of randomized controlled trials. J Eval Clin Pract 26(3):842–851. 10.1111/jep.13261 31436024 10.1111/jep.13261

[71] Rich MW, Chyun DA, Skolnick AH, Alexander KP, Forman DE, Kitzman DW et al (2016) Knowledge gaps in cardiovascular care of the older adult population: a scientific statement from the American Heart Association, American College of Cardiology, and American Geriatrics Society. Circulation 133(21):2103–2122. 10.1161/Cir.0000000000000380 27067230 10.1161/CIR.0000000000000380

[72] Nanna MG, Chen ST, Nelson AJ, Navar AM, Peterson ED (2020) Representation of older adults in cardiovascular disease trials since the inclusion across the lifespan policy. Jama Intern Med 180(11):1531–1533. 10.1001/jamainternmed.2020.2750 32897289 10.1001/jamainternmed.2020.2750 PMC7489390

[73] Wallach JD, Glick L, Gueorguieva R, O’Malley SS (2024) Evidence of subgroup differences in meta-analyses evaluating medications for alcohol use disorder: an umbrella review. Alcohol Clin Exp Res (Hoboken) 48(1):5–15. 10.1111/acer.15229 38102794 10.1111/acer.15229 PMC10841726

[74] Fusar-Poli P, Radua J (2018) Ten simple rules for conducting umbrella reviews. Evid-Based Ment Health 21(3):95–100. 10.1136/ebmental-2018-300014 30006442 10.1136/ebmental-2018-300014 PMC10270421

[75] Rahimi K, Bidel Z, Nazarzadeh M, Copland E, Canoy D, Wamil M et al (2021) Age-stratified and blood-pressure-stratified effects of blood-pressure-lowering pharmacotherapy for the prevention of cardiovascular disease and death: an individual participant-level data meta-analysis. Lancet 398(10305):1053–1064. 10.1016/S0140-6736(21)01921-8 34461040 10.1016/S0140-6736(21)01921-8 PMC8473559

[76] Steinman MA, Boyd CM, Schmader KE (2021) Expanding evidence for clinical care of older adults beyond clinical trial traditions and finding new approaches. JAMA-J Am Med Assoc 326(6):475–476. 10.1001/jama.2021.12134 10.1001/jama.2021.12134 PMC11208068 34292309

[77] Abdelazeem B, Elbadawy MA, Awad AK, Kheiri B, Kunadi A (2022) The cardiovascular outcomes of finerenone in patients with chronic kidney disease and type 2 diabetes: a meta-analysis of randomized clinical trials. Intractable Rare Dis Res. 10.5582/irdr.2020.01008 35261849 10.5582/irdr.2020.01008 PMC8898394

[78] Abraham B, Megaly M, Sous M, Fransawyalkomos M, Saad M, Fraser R et al (2020) Meta-analysis comparing torsemide versus furosemide in patients with heart failure. Am J Cardiol 125(1):92–99. 10.1016/j.amjcard.2019.09.039 31699358 10.1016/j.amjcard.2019.09.039

[79] Ahmed M, Nudy M, Bussa R, Filippone EJ, Foy AJ (2023) A systematic review and meta-analysis of all sham and placebo controlled trials for resistant hypertension. Eur J Intern Med 113:83–90. 10.1016/j.ejim.2023.04.021 37150718 10.1016/j.ejim.2023.04.021

[80] Albasri A, Hattle M, Koshiaris C, Dunnigan A, Paxton B, Fox SE et al (2021) Association between anti-hypertensive treatment and adverse events: systematic review and meta-analysis. BMJ 372:n189. 10.1136/bmj.n189 33568342 10.1136/bmj.n189 PMC7873715

[81] Alexandrou ME, Papagianni A, Tsapas A, Loutradis C, Boutou A, Piperidou A et al (2019) Effects of mineralocorticoid receptor antagonists in proteinuric kidney disease: a systematic review and meta-analysis of randomized controlled trials. J Hypertens 37(12):2307–2324. 10.1097/hjh.0000000000002187 31688290 10.1097/HJH.0000000000002187

[82] Al-Sadawi M, Tao M, Dhaliwal S, Goldschmit M, Tam E, Mann N (2024) Safety and efficacy of anti-hypertensive medications in patients with heart failure with preserved ejection fraction: a systematic review and meta-analysis. High Blood Press Cardiovasc Prev 31(3):239–249. 10.1007/s40292-024-00646-0 38740725 10.1007/s40292-024-00646-0

[83] Asiimwe IG, Pushpakom S, Turner RM, Kolamunnage-Dona R, Jorgensen AL, Pirmohamed M (2021) Cardiovascular drugs and COVID-19 clinical outcomes: a living systematic review and meta-analysis. Br J Clin Pharmacol 87(12):4534–4545. 10.1111/bcp.14927 34101232 10.1111/bcp.14927 PMC8239929

[84] Bao W, Zhang M, Li N, Yao Z, Sun L (2022) Efficacy and safety of finerenone in chronic kidney disease associated with type 2 diabetes: a systematic review and meta-analysis of randomized clinical trials. Eur J Clin Pharmacol 78(12):1877–1887. 10.1007/s00228-022-03408-w 36273065 10.1007/s00228-022-03408-w

[85] Bazoukis G, Thomopoulos C, Tse G, Tsioufis C (2018) Is there a blood pressure lowering effect of MRAs in heart failure? An overview and meta-analysis. Heart Fail Rev 23(4):547–553. 10.1007/s10741-018-9689-9 29527640 10.1007/s10741-018-9689-9

[86] Bazoukis G, Thomopoulos C, Tsioufis C (2018) Effect of mineralocorticoid antagonists on blood pressure lowering: overview and meta-analysis of randomized controlled trials in hypertension. J Hypertens 36(5):987–994. 10.1097/hjh.0000000000001671 29356711 10.1097/HJH.0000000000001671

[87] Bonsu KO, Arunmanakul P, Chaiyakunapruk N (2018) Pharmacological treatments for heart failure with preserved ejection fraction-a systematic review and indirect comparison. Heart Fail Rev 23(2):147–156. 10.1007/s10741-018-9679-y 29411216 10.1007/s10741-018-9679-y

[88] Boulmpou A, Theodorakopoulou MP, Alexandrou ME, Boutou AK, Papadopoulos CE, Pella E et al (2022) Meta-analysis addressing the impact of cardiovascular-acting medication on peak oxygen uptake of patients with HFpEF. Heart Fail Rev 27(2):609–623. 10.1007/s10741-021-10207-5 35067835 10.1007/s10741-021-10207-5

[89] Chen Q, Wei G, Wang Y, Li X, Zhao Q, Zhu L et al (2024) Efficacy and safety of nonsteroidal mineralocorticoid receptor antagonists for renal and cardiovascular outcomes in patients with chronic kidney disease: a meta-analysis of randomized clinical trials. Front Pharmacol 15:1338044. 10.3389/fphar.2024.1338044 38476327 10.3389/fphar.2024.1338044 PMC10927749

[90] Clark KM, Mahboob F, Evans J, Sun JH, Wang N (2024) Efficacy of guideline-directed medical therapy in heart failure patients with and without chronic kidney disease: a meta-analysis of 63,677 patients. Heart Lung Circ 33(3):281–291. 10.1016/j.hlc.2023.12.013 38365495 10.1016/j.hlc.2023.12.013

[91] Dineva S, Uzunova K, Pavlova V, Filipova E, Kalinov K, Vekov T (2019) Comparative efficacy and safety of chlorthalidone and hydrochlorothiazide-meta-analysis. J Hum Hypertens 33(11):766–774. 10.1038/s41371-019-0255-2 31595024 10.1038/s41371-019-0255-2 PMC6892412

[92] Dineva S, Uzunova K, Pavlova V, Filipova E, Kalinov K, Vekov T (2020) Network meta-analysis of efficacy and safety of chlorthalidone and hydrochlorothiazide in hypertensive patients. Blood Press Monit 04:160–168. 10.1097/mbp.0000000000000486 10.1097/MBP.0000000000000486 PMC7932752 32909966

[93] Ding K, Li Z, Lu Y, Sun L (2023) Efficacy and safety assessment of mineralocorticoid receptor antagonists in patients with chronic kidney disease. Eur J Intern Med 115:114–127. 10.1016/j.ejim.2023.05.038 37328398 10.1016/j.ejim.2023.05.038

[94] Du Y, Cao G, Gu L, Chen Y, Liu J (2023) Tumor risks of finerenone in patients with type 2 diabetes mellitus complicated with chronic kidney disease: a meta-analysis and systematic review of randomized controlled trials. Front Pharmacol 14:1237583. 10.3389/fphar.2023.1237583 38273834 10.3389/fphar.2023.1237583 PMC10808358

[95] Dutta D, Surana V, Bhattacharya S, Aggarwal S, Sharma M (2022) Efficacy and safety of novel non-steroidal mineralocorticoid receptor antagonist finerenone in the management of diabetic kidney disease: a meta-analysis. Indian J Endocrinol Metab 26(3):198–205. 10.4103/ijem.ijem_376_21 36248038 10.4103/ijem.ijem_376_21 PMC9555385

[96] Eid PS, Ibrahim DA, Zayan AH, Elrahman MMA, Shehata MAA, Kandil H et al (2021) Comparative effects of furosemide and other diuretics in the treatment of heart failure: a systematic review and combined meta-analysis of randomized controlled trials. Heart Fail Rev 26(1):127–136. 10.1007/s10741-020-10003-7 32783109 10.1007/s10741-020-10003-7

[97] Elshahat A, Mansour A, Ellabban M, Diaa A, Hassan A, Fawzy A et al (2024) Comparative effectiveness and safety of eplerenone and spironolactone in patients with heart failure: a systematic review and meta-analysis. BMC Cardiovasc Disord 24(1):489. 10.1186/s12872-024-04103-7 39271992 10.1186/s12872-024-04103-7 PMC11395778

[98] Faisal S, Ahmad Ganaie Z, Batool S, Lokhandwala DHI, Hankins J, Chaudhari SS et al (2022) The efficacy of various pharmacological agents on long-term outcomes in patients with heart failure with preserved ejection fraction: a meta-analysis of randomized control trials. Cureus 14(8):e28145. 10.7759/cureus.28145 36148200 10.7759/cureus.28145 PMC9482452

[99] Farmakis IT, Pyrgidis N, Doundoulakis I, Mykoniatis I, Akrivos E, Giannakoulas G (2022) Effects of major antihypertensive drug classes on erectile function: a network meta-analysis. Cardiovasc Drugs Ther 36(5):903–914. 10.1007/s10557-021-07197-9 33945044 10.1007/s10557-021-07197-9

[100] Fatima K, Asad D, Shaikh N, Ansari SA, Kumar G, Rehman HA et al (2023) A Systematic review and meta-analysis on effectiveness of mineralocorticoid receptor antagonists in reducing the risk of atrial fibrillation. Am J Cardiol 199:85–91. 10.1016/j.amjcard.2023.04.038 37269781 10.1016/j.amjcard.2023.04.038

[101] Fernandes BP, Conceicao LSR, Martins-Filho PRS, de Santana Motta DRM, Carvalho VO (2018) Effect of mineralocorticoid receptor antagonists in individuals with heart failure with preserved ejection fraction: a systematic review. J Card Fail 24(9):618–621. 10.1016/j.cardfail.2018.08.006 30194985 10.1016/j.cardfail.2018.08.006

[102] Ferre N, Parada E, Balaguer A, Feliu A, Roque-Figuls M, Franco JVA et al (2022) Pharmacological interventions for preventing complications in patients with idiopathic hypercalciuria: a systematic review. Nefrologia (Engl Ed) 42(5):506–518. 10.1016/j.nefroe.2021.04.014 36792305 10.1016/j.nefroe.2021.04.014

[103] Frankenstein L, Seide S, Tager T, Jensen K, Frohlich H, Clark AL et al (2020) Relative Efficacy of Spironolactone, Eplerenone, and cAnRenone in patients with Chronic Heart failure (RESEARCH): a systematic review and network meta-analysis of randomized controlled trials. Heart Fail Rev 25(2):161–171. 10.1007/s10741-019-09832-y 31364027 10.1007/s10741-019-09832-y

[104] Fu Z, Geng X, Chi K, Song C, Wu D, Liu C et al (2021) Efficacy and safety of finerenone in patients with chronic kidney disease: a systematic review with meta-analysis and trial sequential analysis. Ann Palliat Med 10(7):7428–7439. 10.21037/apm-21-763 34353035 10.21037/apm-21-763

[105] Fukuta H, Goto T, Wakami K, Kamiya T, Ohte N (2019) Effects of mineralocorticoid receptor antagonists on left ventricular diastolic function, exercise capacity, and quality of life in heart failure with preserved ejection fraction: a meta-analysis of randomized controlled trials. Heart Vessels 34(4):597–606. 10.1007/s00380-018-1279-1 30315496 10.1007/s00380-018-1279-1

[106] Ghosal S, Sinha B (2023) Finerenone in type 2 diabetes and renal outcomes: a random-effects model meta-analysis. Front Endocrinol (Lausanne) 14:1114894. 10.3389/fendo.2023.1114894 36742404 10.3389/fendo.2023.1114894 PMC9895809

[107] Ghosal S, Sinha B (2023) Assessing the effects of modern renoprotective agents in preventing progression of renal composite outcomes in patients with type 2 diabetes: a network meta-analysis. Diabetes Therapy 14(2):415–424. 10.1007/s13300-022-01359-0 36566447 10.1007/s13300-022-01359-0 PMC9943809

[108] Gu X, Jiang S, Yang Y, Li W (2024) Effects of finerenone and glucagon-like peptide 1 receptor agonists on cardiovascular and renal outcomes in type 2 diabetes mellitus: a systematic review and meta-analysis. Diabetol Metab Syndr 16(1):14. 10.1186/s13098-023-01251-2 38212831 10.1186/s13098-023-01251-2 PMC10782753

[109] Hall JJ, Eurich DT, Nagy D, Tjosvold L, Gamble JM (2020) Thiazide diuretic-induced change in fasting plasma glucose: a meta-analysis of randomized clinical trials. J Gen Intern Med 35(6):1849–1860. 10.1007/s11606-020-05731-3 32157653 10.1007/s11606-020-05731-3 PMC7280437

[110] Hansen MR, Hrobjartsson A, Videbaek L, Ennis ZN, Pareek M, Paulsen NH et al (2020) Postponement of death by pharmacological heart failure treatment: a meta-analysis of randomized clinical trials. Am J Med 133(6):e280–e289. 10.1016/j.amjmed.2019.11.015 32173347 10.1016/j.amjmed.2019.11.015

[111] Harrington J, Fonarow GC, Khan MS, Hernandez A, Anker S, Bohm M et al (2023) Medication-attributable adverse events in heart failure trials. JACC Heart Fail. 10.1016/j.jchf.2022.11.026 36881395 10.1016/j.jchf.2022.11.026 PMC10084875

[112] Hasegawa T, Nishiwaki H, Ota E, Levack WM, Noma H (2021) Aldosterone antagonists for people with chronic kidney disease requiring dialysis. Cochrane Database Syst Rev 2:CD013109. 10.1002/14651858.cd013109.pub2 33586138 10.1002/14651858.CD013109.pub2 PMC8094170

[113] Ho WY, Hsiao CC, Wu PH, Chen JY, Tu YK, Wu VC et al (2024) Comparison of different medical treatments for primary hyperaldosteronism: a systematic review and network meta-analysis. Ther Adv Chronic Dis 15:20406223241239776. 10.1177/20406223241239775 38511069 10.1177/20406223241239775 PMC10953100

[114] Hu H, Zhao X, Jin X, Wang S, Liang W, Cong X (2022) Efficacy and safety of eplerenone treatment for patients with diabetic nephropathy: a meta-analysis. PLoS ONE 17(3):e0265642. 10.1371/journal.pone.0265642 35324976 10.1371/journal.pone.0265642 PMC8947092

[115] Jiang X, Zhang Z, Li C, Zhang S, Su Q, Yang S et al (2022) Efficacy and safety of non-steroidal mineralocorticoid receptor antagonists in patients with chronic kidney disease and type 2 diabetes: a systematic review incorporating an indirect comparisons meta-analysis. Front Pharmacol 13:896947. 10.3389/fphar.2022.896947 35784710 10.3389/fphar.2022.896947 PMC9243561

[116] Jyotsna F, Mahfooz K, Patel T, Parshant F, Simran F, Harsha F et al (2023) A systematic review and meta-analysis on the efficacy and safety of finerenone therapy in patients with cardiovascular and chronic kidney diseases in type 2 diabetes mellitus. Cureus 15(7):e41746. 10.7759/cureus.41746 37575756 10.7759/cureus.41746 PMC10421409

[117] Kapelios CJ, Murrow JR, Nuhrenberg TG, Montoro Lopez MN (2019) Effect of mineralocorticoid receptor antagonists on cardiac function in patients with heart failure and preserved ejection fraction: a systematic review and meta-analysis of randomized controlled trials. Heart Fail Rev 24(3):367–377. 10.1007/s10741-018-9758-0 30618017 10.1007/s10741-018-9758-0 PMC6477010

[118] Karakasis P, Patoulias D, Popovic DS, Pamporis K, Theofilis P, Nasoufidou A et al (2024) Effects of mineralocorticoid receptor antagonists on new-onset or recurrent atrial fibrillation: a Bayesian and frequentist network meta-analysis of randomized trials. Curr Probl Cardiol 49(9):102742. 10.1016/j.cpcardiol.2024.102742 39002620 10.1016/j.cpcardiol.2024.102742

[119] Kido K, Shimizu M, Hashiguchi M (2019) Comparing torsemide versus furosemide in patients with heart failure: a meta-analysis. J Am Pharm Assoc (2003) 59(3):432–438. 10.1016/j.japh.2019.01.014 30846351 10.1016/j.japh.2019.01.014

[120] Kohjimoto Y, Iba A, Yamashita S, Higuchi M, Deguchi R, Chikazawa I et al (2024) Pharmacotherapy for patients with calcium oxalate stones and abnormal urine chemistry: a systematic review and meta-analysis for the Japanese clinical practice guidelines for the management of urinary stones, third edition. Int J Urol 16:16. 10.1111/iju.15608 10.1111/iju.15608 39415439

[121] Li DF, Gao YL, Liu HC, Huang XC, Zhu RF, Zhu CT (2020) Use of thiazide diuretics for the prevention of recurrent kidney calculi: a systematic review and meta-analysis. J Transl Med 18(1):106. 10.1186/s12967-020-02270-7 32111248 10.1186/s12967-020-02270-7 PMC7048029

[122] Li X, Wu H, Peng H, Jiang H (2022) Comparison the effects of finerenone and SGLT2i on cardiovascular and renal outcomes in patients with type 2 diabetes mellitus: a network meta-analysis. Front Endocrinol (Lausanne) 13:1078686. 10.3389/fendo.2022.1078686 36589800 10.3389/fendo.2022.1078686 PMC9797657

[123] Li J, Chen Y, Wang Y, Liu X, Li P, He Y et al (2024) Impact of guideline-directed medical therapy on systolic blood pressure and cardiovascular outcomes in patients with heart failure and low blood pressure: a systematic review and meta-analysis. Eur J Heart Fail 26(6):1435–1442. 10.1002/ejhf.3208 38606573 10.1002/ejhf.3208

[124] Liu J, Jia W, Yu C (2022) Safety and efficacy of spironolactone in dialysis-dependent patients: meta-analysis of randomized controlled trials. Front Med (Lausanne) 9:828189. 10.3389/fmed.2022.828189 35372414 10.3389/fmed.2022.828189 PMC8970057

[125] Lunney M, Ruospo M, Natale P, Quinn RR, Ronksley PE, Konstantinidis I et al (2020) Pharmacological interventions for heart failure in people with chronic kidney disease. Cochrane Database Syst Rev 2:CD012466. 10.1002/14651858.cd012466.pub2 32103487 10.1002/14651858.CD012466.pub2 PMC7044419

[126] Ma L, Zheng K, Yan J, Cheng W (2021) Efficacy of ARB/HCTZ combination therapy in uncontrolled hypertensive patients compared with ARB monotherapy: a meta-analysis. Int J Hypertens 2021:6670183. 10.1155/2021/6670183 33996152 10.1155/2021/6670183 PMC8096582

[127] Macfarlane TV, Pigazzani F, Flynn RWV, MacDonald TM (2019) The effect of indapamide vs. bendroflumethiazide for primary hypertension: a systematic review. Br J Clin Pharmacol 85(2):285–303. 10.1111/bcp.13787 30312512 10.1111/bcp.13787 PMC6339968

[128] Martin N, Manoharan K, Davies C, Lumbers RT (2021) Beta-blockers and inhibitors of the renin-angiotensin aldosterone system for chronic heart failure with preserved ejection fraction. Cochrane Database Syst Rev 5:CD012721. 10.1002/14651858.cd012721.pub3 34022072 10.1002/14651858.CD012721.pub3 PMC8140651

[129] Martins VM, Ziegelmann PK, Ferrari F, Bottino LG, Lucca MB, Correa HLR et al (2023) Thiazide diuretics alone or combined with potassium-sparing diuretics to treat hypertension: a systematic review and network meta-analysis of randomized controlled trials. J Hypertens 41(7):1108–1116. 10.1097/hjh.0000000000003436 37016911 10.1097/HJH.0000000000003436 PMC10241430

[130] Miles JA, Hanumanthu BK, Patel K, Chen M, Siegel RM, Kokkinidis DG (2019) Torsemide versus furosemide and intermediate-term outcomes in patients with heart failure: an updated meta-analysis. J Cardiovasc Med (Hagerstown) 20(6):379–388. 10.2459/jcm.0000000000000794 30950982 10.2459/JCM.0000000000000794

[131] Morita R, Tsukamoto S, Obata S, Yamada T, Uneda K, Uehara T et al (2023) Effects of sodium-glucose cotransporter 2 inhibitors, mineralocorticoid receptor antagonists, and their combination on albuminuria in diabetic patients. Diabetes Obes Metab. 10.1111/dom.14976 36633511 10.1111/dom.14976

[132] Nguyen BN, Nguyen L, Mital S, Bugden S, Nguyen HV (2023) Comparative efficacy of sodium-glucose co-transporter-2 inhibitors, glucagon-like peptide-1 receptor agonists and non-steroidal mineralocorticoid receptor antagonists in chronic kidney disease and type 2 diabetes: a systematic review and network meta-anal. Diabetes Obes Metab 08:08. 10.1111/dom.15009 10.1111/dom.15009 36751968

[133] Noone C, Leahy J, Morrissey EC, Newell J, Newell M, Dwyer CP et al (2020) Comparative efficacy of exercise and anti-hypertensive pharmacological interventions in reducing blood pressure in people with hypertension: a network meta-analysis. Eur J Prev Cardiol 27(3):247–255. 10.1177/2047487319879786 31615283 10.1177/2047487319879786

[134] Oraii A, Healey JS, Kowalik K, Pandey AK, Benz AP, Wong JA et al (2024) Mineralocorticoid receptor antagonists and atrial fibrillation: a meta-analysis of clinical trials. Eur Heart J 45(10):756–774. 10.1093/eurheartj/ehad811 38195054 10.1093/eurheartj/ehad811

[135] Pamporis K, Karakasis P, Sagris M, Zarifis I, Bougioukas KI, Pagkalidou E et al (2024) Mineralocorticoid receptor antagonists in heart failure with reduced ejection fraction: a systematic review and network meta-analysis of 32 randomized trials. Curr Probl Cardiol 49(7):102615. 10.1016/j.cpcardiol.2024.102615 38692445 10.1016/j.cpcardiol.2024.102615

[136] Patoulias D, Papadopoulos C, Toumpourleka M, Doumas M (2021) Meta-analysis addressing the effect of mineralcorticoid receptor antagonists on the risk for new-onset atrial fibrillation. Am J Cardiol 157:150–152. 10.1016/j.amjcard.2021.07.011 34399968 10.1016/j.amjcard.2021.07.011

[137] Patoulias D, Papadopoulos C, Karagiannis A, Vassilikos V, Doumas M (2022) Cardiovascular outcomes with finerenone according to glycemic status at baseline and prior treatment with newer antidiabetics among patients with type 2 diabetes mellitus. Endocrinol Metab (Seoul) 37(1):170–174. 10.3803/enm.2021.1296 35144333 10.3803/EnM.2021.1296 PMC8901958

[138] Peters R, Yasar S, Anderson CS, Andrews S, Antikainen R, Arima H et al (2019) An investigation of anti-hypertensive class, dementia, and cognitive decline: a meta-analysis. Neurology 11:1–15. 10.1212/wnl.0000000000008732 10.1212/WNL.0000000000008732 PMC7108807 31827004

[139] Sampaio Rodrigues T, Garcia Quarto LJ, Nogueira SC, Koshy AN, Mahajan R, Sanders P et al (2024) Incidence and progression of atrial fibrillation in patients with and without heart failure using mineralocorticoid receptor antagonists: a meta-analysis. Clin Res Cardiol 113(6):884–897. 10.1007/s00392-023-02349-3 38170251 10.1007/s00392-023-02349-3

[140] Shi Q, Nong K, Vandvik PO, Guyatt GH, Schnell O, Ryden L et al (2023) Benefits and harms of drug treatment for type 2 diabetes: systematic review and network meta-analysis of randomised controlled trials. BMJ. 10.1136/bmj-2022-074068 37024129 10.1136/bmj-2022-074068 PMC10077111

[141] Siddiqi AK, Javaid H, Ahmed M, Dhawadi S, Batool L, Zahid M et al (2023) Clinical outcomes with furosemide versus torsemide in patients with heart failure: an updated systematic review and meta-analysis. Curr Probl Cardiol 48(11):101927. 10.1016/j.cpcardiol.2023.101927 37453532 10.1016/j.cpcardiol.2023.101927

[142] Singh S, Goel S, Duhan S, Chaudhary R, Garg A, Tantry US et al (2023) Effect of furosemide versus torsemide on hospitalizations and mortality in patients with heart failure: a meta-analysis of randomized controlled trials. Am J Cardiol 206:42–48. 10.1016/j.amjcard.2023.08.079 37677884 10.1016/j.amjcard.2023.08.079 PMC10824237

[143] Sreenivasan J, Malik A, Khan MS, Lloji A, Hooda U, Aronow WS et al (2024) Pharmacotherapies in heart failure with preserved ejection fraction: a systematic review and network meta-analysis. Cardiol Rev 32(2):114–123. 10.1097/crd.0000000000000484 36576372 10.1097/CRD.0000000000000484

[144] Sreenivasan J, Malik A, Khan MS, Lloji A, Hooda U, Aronow WS et al (2022) Pharmacotherapies in heart failure with preserved ejection fraction: a systematic review and network meta-analysis. Cardiol Rev 27:27. 10.1097/crd.0000000000000484 10.1097/CRD.0000000000000484 36576372

[145] Tager T, Frohlich H, Seiz M, Katus HA, Frankenstein L (2019) READY: relative efficacy of loop diuretics in patients with chronic systolic heart failure—a systematic review and network meta-analysis of randomised trials. Heart Fail Rev 24(4):461–472. 10.1007/s10741-019-09771-8 30874955 10.1007/s10741-019-09771-8

[146] Teixeira L, Felix N, Navalha DDP, Ferreira R, Clemente MRC, Madeira T et al (2024) Torsemide versus furosemide in the treatment of heart failure: a systematic review and meta-analysis of randomized controlled trials. Arq Bras Cardiol 121(6):e20230825. 10.36660/abc.20230825i10.36660/abc.20230825 39046046 10.36660/abc.20230825 PMC12080595

[147] Teles F, Pecanha de Miranda Coelho JA, Albino RM, Vercosa Pacheco FC, Rodrigues de Oliveira E, Silveira MAD et al (2023) Effectiveness of thiazide and thiazide-like diuretics in advanced chronic kidney disease: a systematic review and meta-analysis. Ren Fail 45(1):2163903. 10.1080/0886022x.2022.2163903 36637019 10.1080/0886022X.2022.2163903 PMC9848247

[148] Tian Z, Barbosa CV, Lang H, Bauersachs J, Melk A, Schmidt BMW (2024) Efficacy of pharmacological and interventional treatment for resistant hypertension: a network meta-analysis. Cardiovasc Res 120(1):108–119. 10.1093/cvr/cvad165 37890022 10.1093/cvr/cvad165

[149] Tsukamoto S, Morita R, Yamada T, Urate S, Azushima K, Uneda K et al (2022) Cardiovascular and kidney outcomes of combination therapy with sodium-glucose cotransporter-2 inhibitors and mineralocorticoid receptor antagonists in patients with type 2 diabetes and chronic kidney disease: a systematic review and network meta-analysis. Diabetes Res Clin Pract 194:110161. 10.1016/j.diabres.2022.110161 36403681 10.1016/j.diabres.2022.110161

[150] Wang N, Evans J, Sawant S, Sindone J, Lal S (2022) Sex-specific differences in the efficacy of heart failure therapies: a meta-analysis of 84,818 patients. Heart Fail Rev. 10.1007/s10741-022-10275-1 36198840 10.1007/s10741-022-10275-1

[151] Wei J, Galaviz KI, Kowalski AJ, Magee MJ, Haw JS, Narayan KMV et al (2020) Comparison of cardiovascular events among users of different classes of antihypertension medications: a systematic review and network meta-analysis. JAMA Netw 3(2):e1921618. 10.1001/jamanetworkopen.2019.21618 10.1001/jamanetworkopen.2019.21618 PMC7043193 32083689

[152] Wright JM, Musini VM, Gill R (2018) First-line drugs for hypertension. Cochrane Database Syst Rev 4:CD001841. 10.1002/14651858.cd001841.pub3 29667175 10.1002/14651858.CD001841.pub3 PMC6513559

[153] Wu Y, Lin H, Tao Y, Xu Y, Chen J, Jia Y et al (2022) Network meta-analysis of mineralocorticoid receptor antagonists for diabetic kidney disease. Front Pharmacol 13:967317. 10.3389/fphar.2022.967317 36188560 10.3389/fphar.2022.967317 PMC9523214

[154] Xiang Y, Shi W, Li Z, Yang Y, Wang SY, Xiang R et al (2019) Efficacy and safety of spironolactone in the heart failure with mid-range ejection fraction and heart failure with preserved ejection fraction: a meta-analysis of randomized clinical trials. Medicine (Baltimore) 98(13):e14967. 10.1097/md.0000000000014967 30921200 10.1097/MD.0000000000014967 PMC6456096

[155] Xiang B, Zhang R, Wu X, Zhou X (2022) Optimal pharmacologic treatment of heart failure with preserved and mildly reduced ejection fraction: a meta-analysis. JAMA Netw 5(9):e2231963. 10.1001/jamanetworkopen.2022.31963 10.1001/jamanetworkopen.2022.31963 PMC9490501 36125813

[156] Xie W, Zheng F, Evangelou E, Liu O, Yang Z, Chan Q et al (2018) Blood pressure-lowering drugs and secondary prevention of cardiovascular disease: systematic review and meta-analysis. J Hypertens 36(6):1256–1265. 10.1097/hjh.0000000000001720 29543625 10.1097/HJH.0000000000001720

[157] Xu Y, Qiu Z, Yang R, Wu Y, Cheng X (2018) Efficacy of mineralocorticoid receptor antagonists in postmyocardial infarction patients with or without left ventricular dysfunction: a meta-analysis of randomized controlled trials. Medicine (Baltimore) 97(51):e13690. 10.1097/md.0000000000013690 30572494 10.1097/MD.0000000000013690 PMC6319977

[158] Xu X, Feng J, Cui Y, Li P, Dong J, Liao L (2024) Renal effects and safety between Asian and non-Asian chronic kidney disease and type 2 diabetes treated with nonsteroidal mineralocorticoid antagonists. J Diabetes 16(6):e13566. 10.1111/1753-0407.13566 38753662 10.1111/1753-0407.13566 PMC11098447

[159] Yanai K, Ishibashi K, Morishita Y (2021) Systematic review and meta-analysis of renin-angiotensin-aldosterone system blocker effects on the development of cardiovascular disease in patients with chronic kidney disease. Front Pharmacol 12:662544. 10.3389/fphar.2021.662544 34276363 10.3389/fphar.2021.662544 PMC8283791

[160] Yang P, Shen W, Chen X, Zhu D, Xu X, Wu T et al (2019) Comparative efficacy and safety of mineralocorticoid receptor antagonists in heart failure: a network meta-analysis of randomized controlled trials. Heart Fail Rev 24(5):637–646. 10.1007/s10741-019-09790-5 31030322 10.1007/s10741-019-09790-5

[161] Yang S, Zhao L, Mi Y, He W (2022) Effects of sodium-glucose cotransporter-2 inhibitors and aldosterone antagonists, in addition to renin-angiotensin system antagonists, on major adverse kidney outcomes in patients with type 2 diabetes and chronic kidney disease: a systematic review and ne. Diabetes Obes Metab 24(11):2159–2168. 10.1111/dom.14801 35712807 10.1111/dom.14801

[162] Yang S, Shen W, Zhang HZ, Wang CX, Yu WQ, Wu QH (2023) Efficacy and safety of finerenone for prevention of cardiovascular events in type 2 diabetes mellitus with chronic kidney disease: a meta-analysis of randomized controlled trials. J Cardiovasc Pharmacol 81(1):55–62. 10.1097/fjc.0000000000001364 36027585 10.1097/FJC.0000000000001364

[163] Yang SQ, Zhao X, Zhang J, Liu H, Wang YH, Wang YG (2024) Comparative efficacy and safety of SGLT2is and ns-MRAs in patients with diabetic kidney disease: a systematic review and network meta-analysis. Front Endocrinol (Lausanne) 15:1429261. 10.3389/fendo.2024.1429261 39027482 10.3389/fendo.2024.1429261 PMC11256196

[164] Yang Q, Lang Y, Yang W, Yang F, Yang J, Wu Y et al (2023) Efficacy and safety of drugs for people with type 2 diabetes mellitus and chronic kidney disease on kidney and cardiovascular outcomes: a systematic review and network meta-analysis of randomized controlled trials. Diabetes Res Clin Pract 198:110592. 10.1016/j.diabres.2023.110592 36842477 10.1016/j.diabres.2023.110592

[165] Yasmin F, Aamir M, Najeeb H, Atif AR, Siddiqui AH, Ahsan MN et al (2023) Efficacy and safety of finerenone in chronic kidney disease and type 2 diabetes patients: a systematic review and meta-analysis. Ann Med Surg (Lond) 85(10):4973–4980. 10.1097/ms9.0000000000001180 37811017 10.1097/MS9.0000000000001180 PMC10553111

[166] Zafeiropoulos S, Farmakis IT, Milioglou I, Doundoulakis I, Gorodeski EZ, Konstantinides SV et al (2024) Pharmacological treatments in heart failure with mildly reduced and preserved ejection fraction: systematic review and network meta-analysis. JACC Heart Fail 12(4):616–627. 10.1016/j.jchf.2023.07.014 37656079 10.1016/j.jchf.2023.07.014

[167] Zeng Q, Zhou X, Xu G (2019) Safety evaluation and cardiovascular effect of additional use of spironolactone in hemodialysis patients: a meta-analysis. Drug Des Dev Ther 13:1487–1499. 10.2147/dddt.s189454 10.2147/DDDT.S189454 PMC6504555 31118582

[168] Zhang Y, Wang J, Jiang L, Wang T, Li Z, Fu X et al (2022) Network meta-analysis on the efficacy and safety of finerenone versus SGLT2 inhibitors on reducing new-onset of atrial fibrillation in patients with type 2 diabetes mellitus and chronic kidney disease. Diabetol Metab Syndr. 10.1186/s13098-022-00929-3 36303247 10.1186/s13098-022-00929-3 PMC9609222

[169] Zhang Y, Jiang L, Wang J, Wang T, Chien C, Huang W et al (2022) Network meta-analysis on the effects of finerenone versus SGLT2 inhibitors and GLP-1 receptor agonists on cardiovascular and renal outcomes in patients with type 2 diabetes mellitus and chronic kidney disease. Cardiovasc Diabetol 21(1):232. 10.1186/s12933-022-01676-5 36335326 10.1186/s12933-022-01676-5 PMC9637313

[170] Zhang MZ, Bao W, Zheng QY, Wang YH, Sun LY (2022) Efficacy and safety of finerenone in chronic kidney disease: a systematic review and meta-analysis of randomized clinical trials. Front Pharmacol 13:819327. 10.3389/fphar.2022.819327 35197856 10.3389/fphar.2022.819327 PMC8859447

[171] Zhao X, Ren Y, Li H, Liu X (2019) The effect of diuretics on patients with heart failure: a network meta-analysis: diuretics effect on heart failure patients. J Pharm Pharm Sci 22(1):270–280. 10.18433/jpps30146 31287791 10.18433/jpps30146

[172] Zheng SL, Chan FT, Nabeebaccus AA, Shah AM, McDonagh T, Okonko DO et al (2018) Drug treatment effects on outcomes in heart failure with preserved ejection fraction: a systematic review and meta-analysis. Heart 104(5):407–415. 10.1136/heartjnl-2017-311652 28780577 10.1136/heartjnl-2017-311652 PMC5861385

[173] Zheng Y, Ma S, Huang Q, Fang Y, Tan H, Chen Y et al (2022) Meta-analysis of the efficacy and safety of finerenone in diabetic kidney disease. Kidney Blood Press Res 47(4):219–228. 10.1159/000521908 35034019 10.1159/000521908

[174] Zhu S, Li J, Zhao X (2020) Comparative risk of new-onset hyperkalemia for anti-hypertensive drugs in patients with diabetic nephropathy: a Bayesian network meta-analysis. Int J Clin Pract. 10.1111/ijcp.13940 33332696 10.1111/ijcp.13940

[175] Zhu Y, Liu Y, Cai R, Zheng D, Liang X, Tao M et al (2021) The safety and efficacy of low-dose mineralocorticoid receptor antagonists in dialysis patients: a meta-analysis. Medicine (Baltimore) 100(8):e24882. 10.1097/md.0000000000024882 33663116 10.1097/MD.0000000000024882 PMC7909172

[176] Zhu Y, Song M, Chen T, Yang Z, Liu Y (2022) Effect of finerenone on cardiovascular events in kidney disease and/or diabetes: a meta analysis of randomized control trials. Int Urol Nephrol. 10.1007/s11255-022-03432-w 36571667 10.1007/s11255-022-03432-w

[177] Zonneveld TP, Richard E, Vergouwen MD, Nederkoorn PJ, de Haan R, Roos YB et al (2018) Blood pressure-lowering treatment for preventing recurrent stroke, major vascular events, and dementia in patients with a history of stroke or transient ischaemic attack. Cochrane Database Syst Rev 7:CD007858. 10.1002/14651858.cd007858.pub2 30024023 10.1002/14651858.CD007858.pub2 PMC6513249

