# Supplementary material for: Effectiveness and safety of chronic diuretic use in older adults: an umbrella review of recently published systematic reviews and meta-analyses of randomized-controlled trials
Source: Eur Geriatr Med. 2025 May 25;16(4):1353–87. doi: 10.1007/s41999-025-01229-5 (PMC12378697; doi:10.1007/s41999-025-01229-5)
Supplement: Supplementary file 1 — Supplementary file1 (DOCX 33 KB) [file 41999_2025_1229_MOESM1_ESM.docx]

**Supplementary Table 1.** Literature search.

|  | Ovid MEDLINE(R) ALL <1946 to November 08, 2024> Search date: 11 November 2024 |  |
| --- | --- | --- |
| # | **Searches** | **Results** |
| 1 | diuretics/ or Receptors, Mineralocorticoid/ or exp sulfonamides/ or exp vasopressin/ or exp thiazides/ | 204353 |
| 2 | diuretic*.ab,kf,ti. | 45548 |
| 3 | (Acidum mersalylicum or Acidum tienilicum or Amilorid* or amiloride or Amipramidin* or Amyloride or Azosemide or bemetanide or Bendrofluazide or Bendroflumethiazid* or bendroflumethiazide or Benzhydroflumethiazide or Benzothiadiazine or Benzthiazide or Benzydroflumethiazide or Bumetanid* or bumetanide or burinex or Buthiazide or Canrenoate or Canrenoic acid or canrenone or Chinetazone or Chlorothiazid* or chlorothiazide or Chlorphthalidolone or Chlortalidon* or chlortalidone or Chlorthiazide or cicletanine or cicletanine or Ciclotiazid* or cidum etacrynicum or clofenamide or clopamid* or clopamide or clopamine or clorexolone or Clorotiazida or Clortalidona or conivaptan or cyclopenthiazide or Cyclothiazid* or cyclothiazide or diapamide or Dihydroflumethazide or Diucardin or diuril or enduron or Eplerenon* or eplerenone or Epoxymexrenone or esidrix or Espironolactona or Etacrinic acid or etacrynic acid or Ethacrynate or Ethacrynic acid or etozolin or ezna or fenquizone or finerenone or frusemid* or furosemid* or furosemide or fursemid or Hidroclorotiazida or Hidroflumetiazid or Hidroflumetiazida or Hydrochlorothiazid* or hydrochlorothiazide or Hydroflumethiazid* or hydroflumethiazide or hydromox or hygroton or Idroflumetiazide or indacrinone or Indapamid* or indapamide or isodapamide or lozol or mebutizide or mefruside or Mersal or mersalyl or Metforylthiadiazin or methyclothiazide or Methylenebutyrylphenoxyacetic acid or meticrane or Metindamide or Metolazon* or metolazone or microzide or Muzolimin* or muzolimine or mykrox or naqua or naturetin or oxodoline or ozolinone or phenoxybenzoic acid or Phthalamodine or Phthalamudine or Piretanid* or piretanide or polythiazide or potassium canrenoate or Quinetazona or Quinethazon* or quinethazone or renese or Spironolacton* or spironolactone or Teridin or Theobromin* or theobromine or thiazide or Thienylic acid or Ticrynafen or tienilic acid or Tienilic acid or tizolemid* or tolvaptan or torasemid* or torasemide or torsemid* or Triamteren* or triamterene or trichlormethiazide or Xipamid* or xipamide or zaroxolyn or mannitol or acetazolamide or aceis or angiotensin or aquaretics or MRAs or mineralocorticoid or vaptans or sulfonamides or (vasopressin and antagonist?) or ENaCs or (epithelial adj2 (channel inhibitors or aldosterone)) or mineralocorticoid analogues or potassium-sparing agent? or thiazid*).mp. | 324143 |
| 4 | or/1-3 [diuretics] | 442571 |
| 5 | Inappropriate ADH Syndrome/ or exp nephrolithiasis/ or hypercalciuria/ or diabetes insipidus/ or hyponatremia/ or hypokalemia/ or edema/ or nephrotic syndrome/ or renal insufficiency, chronic/ or Hyperaldosteronism/ or proteinuria/ | 182838 |
| 6 | (Inappropriate ADH Syndrome or "syndrome of inappropriate ADH" or "syndrome of antidiuretic hormone" or SIADH or ((congestive or chronic) adj2 heart failure) or kidney stones or kidney calculi or nephrolithiasis or hypercalciuria or diabetes insipidus or hyponatremia or hypokalemia or edema or nephrotic syndrome or chronic kidney disease or chronic renal insufficiency or ((uncontrolled or resistant) adj2 (hypertension or high blood pressure)) or (ascites and cirrhosis) or Hyperaldosteronism or aldosteronism or proteinuria or proteinuric kidney disease).ab,kf,ti. | 413822 |
| 7 | ((heart or cardiac or cardiovascular or stroke) and (prevention or risk)).mp. or pc.fs. | 2110726 |
| 8 | or/5-7 [disorders/indications leading to chronic administration of diuretics] | 2523185 |
| 9 | (Inappropriate ADH Syndrome/ or exp nephrolithiasis/ or hypercalciuria/ or diabetes insipidus/ or hyponatremia/ or hypokalemia/ or edema/ or nephrotic syndrome/ or renal insufficiency, chronic/ or Hyperaldosteronism/ or proteinuria/) and ci.fs. | 20482 |
| 10 | ((((Inappropriate ADH Syndrome or "syndrome of inappropriate ADH" or "syndrome of antidiuretic hormone" or SIADH or ((congestive or chronic) adj2 heart failure) or kidney stones or kidney calculi or nephrolithiasis or hypercalciuria or diabetes insipidus or hyponatremia or hypokalemia or edema or nephrotic syndrome or chronic kidney disease or chronic renal insufficiency or (uncontrolled or resistant)) adj2 (hypertension or high blood pressure)) or (ascites and cirrhosis) or Hyperaldosteronism or aldosteronism or proteinuria or proteinuric kidney disease) adj1 (drug induced or chemically)).ab,kf,ti. | 20 |
| 11 | 9 or 10 | 20492 |
| 12 | heart failure/ or pulmonary edema/ or hypercalcemia/ | 183822 |
| 13 | (heart failure or (hypertensive adj3 (emergen* or crisis)) or lung edema or acute pulmonary edema or hypercalcemia).ab,kf,ti. | 261542 |
| 14 | 12 or 13 [disorders/indications not leading to chronic administration of diuretics] | 318803 |
| 15 | (diabetes or diabetic* or cancer? or malign* or benign* or tumo?r? or neoplasm? or gout or fracture? or bone? or dementia or falls or cognition or cognitive).mp. [off-target effects] | 7790031 |
| 16 | ae.fs. or (adverse effect? or adverse event? or risk).mp. or incidence.sh. or exp mortality/ or follow-up studies.sh. or prognos:.tw. or predict:.tw. or course:.tw. [adverse events or observational longitudinal design] | 8489666 |
| 17 | (systematic review or meta-analysis).pt. | 367411 |
| 18 | meta-analysis/ or systematic review/ or systematic reviews as topic/ or meta-analysis as topic/ or "meta analysis (topic)"/ or "systematic review (topic)"/ or network meta-analysis/ | 398371 |
| 19 | ((systematic* adj3 (review* or overview*)) or (methodologic* adj3 (review* or overview*))).ti,ab,kf. | 388256 |
| 20 | ((quantitative adj3 (review* or overview* or synthes*)) or (research adj3 (integrati* or overview*))).ti,ab,kf. | 18509 |
| 21 | umbrella review*.ti,ab,kf. | 2402 |
| 22 | (multi* adj2 paramet* adj2 evidence adj2 synthesis).ti,ab,kf. | 15 |
| 23 | (multiparamet* adj2 evidence adj2 synthesis).ti,ab,kf. | 19 |
| 24 | (multi-paramet* adj2 evidence adj2 synthesis).ti,ab,kf. | 13 |
| 25 | or/17-24 [SR filter] | 526691 |
| 26 | 4 and (8 or 11) and 25 | 2425 |
| 27 | and/4,14,25 | 977 |
| 28 | (4 or 11) and 15 and 16 and 25 | 1073 |
| 29 | or/26-28 | 3114 |
| 30 | exp animals/ not humans/ | 5274575 |
| 31 | 29 not 30 | 3099 |
|  | | |
|  | **Ovid Embase Classic+Embase <1947 to 2024 November 08> Search date: 11 November 2024** |  |
| # | **Searches** | **Results** |
| 1 | *diuretic agent/ or *Mineralocorticoid receptor/ or exp *sulfonamide/ or *vasopressin/ or exp *thiazide diuretic agent/ | 189023 |
| 2 | diuretic*.ab,kw,ti. | 73797 |
| 3 | (Acidum mersalylicum or Acidum tienilicum or Amilorid* or amiloride or Amipramidin* or Amyloride or Azosemide or bemetanide or Bendrofluazide or Bendroflumethiazid* or bendroflumethiazide or Benzhydroflumethiazide or Benzothiadiazine or Benzthiazide or Benzydroflumethiazide or Bumetanid* or bumetanide or burinex or Buthiazide or Canrenoate or Canrenoic acid or canrenone or Chinetazone or Chlorothiazid* or chlorothiazide or Chlorphthalidolone or Chlortalidon* or chlortalidone or Chlorthiazide or cicletanine or cicletanine or Ciclotiazid* or cidum etacrynicum or clofenamide or clopamid* or clopamide or clopamine or clorexolone or Clorotiazida or Clortalidona or conivaptan or cyclopenthiazide or Cyclothiazid* or cyclothiazide or diapamide or Dihydroflumethazide or Diucardin or diuril or enduron or Eplerenon* or eplerenone or Epoxymexrenone or esidrix or Espironolactona or Etacrinic acid or etacrynic acid or Ethacrynate or Ethacrynic acid or etozolin or ezna or fenquizone or finerenone or frusemid* or furosemid* or furosemide or fursemid or Hidroclorotiazida or Hidroflumetiazid or Hidroflumetiazida or Hydrochlorothiazid* or hydrochlorothiazide or Hydroflumethiazid* or hydroflumethiazide or hydromox or hygroton or Idroflumetiazide or indacrinone or Indapamid* or indapamide or isodapamide or lozol or mebutizide or mefruside or Mersal or mersalyl or Metforylthiadiazin or methyclothiazide or Methylenebutyrylphenoxyacetic acid or meticrane or Metindamide or Metolazon* or metolazone or microzide or Muzolimin* or muzolimine or mykrox or naqua or naturetin or oxodoline or ozolinone or phenoxybenzoic acid or Phthalamodine or Phthalamudine or Piretanid* or piretanide or polythiazide or potassium canrenoate or Quinetazona or Quinethazon* or quinethazone or renese or Spironolacton* or spironolactone or Teridin or Theobromin* or theobromine or thiazide or Thienylic acid or Ticrynafen or tienilic acid or Tienilic acid or tizolemid* or tolvaptan or torasemid* or torasemide or torsemid* or Triamteren* or triamterene or trichlormethiazide or Xipamid* or xipamide or zaroxolyn or mannitol or acetazolamide or aceis or angiotensin or aquaretics or MRAs or mineralocorticoid or vaptans or sulfonamides or (vasopressin and antagonist?) or ENaCs or (epithelial adj2 (channel inhibitors or aldosterone)) or mineralocorticoid analogues or potassium-sparing agent? or thiazid*).mp. | 536751 |
| 4 | or/1-3 [diuretics] | 686193 |
| 5 | *nephrolithiasis/ or *hypercalciuria/ or *diabetes insipidus/ or *hyponatremia/ or *hypokalemia/ or *edema/ or *nephrotic syndrome/ or *chronic kidney failure/ or *Hyperaldosteronism/ or *proteinuria/ | 174242 |
| 6 | (Inappropriate ADH Syndrome or "syndrome of inappropriate ADH" or "syndrome of antidiuretic hormone" or SIADH or ((congestive or chronic) adj2 heart failure) or kidney stones or kidney calculi or nephrolithiasis or hypercalciuria or diabetes insipidus or hyponatremia or hypokalemia or edema or nephrotic syndrome or chronic kidney disease or chronic renal insufficiency or ((uncontrolled or resistant) adj2 (hypertension or high blood pressure)) or (ascites and cirrhosis) or Hyperaldosteronism or aldosteronism or proteinuria or proteinuric kidney disease).ab,kw,ti. | 652990 |
| 7 | ((heart or cardiac or cardiovascular or stroke) and (prevention or risk)).mp. | 1452898 |
| 8 | or/5-7 [disorders/indications leading to chronic administration of diuretics] | 2076544 |
| 9 | (*nephrolithiasis/ or *hypercalciuria/ or *diabetes insipidus/ or *hyponatremia/ or *hypokalemia/ or *edema/ or *nephrotic syndrome/ or *chronic kidney failure/ or *Hyperaldosteronism/ or *proteinuria/) and chemically induced.mp. | 2001 |
| 10 | ((((Inappropriate ADH Syndrome or "syndrome of inappropriate ADH" or "syndrome of antidiuretic hormone" or SIADH or ((congestive or chronic) adj2 heart failure) or kidney stones or kidney calculi or nephrolithiasis or hypercalciuria or diabetes insipidus or hyponatremia or hypokalemia or edema or nephrotic syndrome or chronic kidney disease or chronic renal insufficiency or (uncontrolled or resistant)) adj2 (hypertension or high blood pressure)) or (ascites and cirrhosis) or Hyperaldosteronism or aldosteronism or proteinuria or proteinuric kidney disease) adj1 (drug induced or chemically)).ab,kw,ti. | 50 |
| 11 | 9 or 10 | 2049 |
| 12 | *heart failure/ or *lung edema/ or *hypercalcemia/ | 167111 |
| 13 | (heart failure or (hypertensive adj3 (emergen* or crisis)) or lung edema or acute pulmonary edema or hypercalcemia).ab,kw,ti. | 436515 |
| 14 | 12 or 13 [disorders/indications not leading to chronic administration of diuretics] | 474337 |
| 15 | (diabetes or diabetic* or cancer? or malign* or benign* or tumo?r? or neoplasm? or gout or fracture? or bone? or dementia or falls or cognition or cognitive).mp. [off-target effects] | 11338237 |
| 16 | (adverse effect? or adverse event? or risk).mp. or incidence.sh. or exp mortality/ or follow-up studies.sh. or prognos:.tw. or predict:.tw. or course:.tw. [adverse events or observational longitudinal design] | 10554509 |
| 17 | meta-analysis/ or "systematic review"/ or network meta-analysis/ | 635526 |
| 18 | ((systematic* adj3 (review* or overview*)) or (methodologic* adj3 (review* or overview*))).ti,ab,kw. | 463854 |
| 19 | ((quantitative adj3 (review* or overview* or synthes*)) or (research adj3 (integrati* or overview*))).ti,ab,kw. | 21217 |
| 20 | umbrella review*.ti,ab,kw. | 2447 |
| 21 | (multi* adj2 paramet* adj2 evidence adj2 synthesis).ti,ab,kw. | 34 |
| 22 | (multiparamet* adj2 evidence adj2 synthesis).ti,ab,kw. | 18 |
| 23 | (multi-paramet* adj2 evidence adj2 synthesis).ti,ab,kw. | 29 |
| 24 | or/17-23 [SR filter] | 756566 |
| 25 | 4 and (8 or 11) and 24 | 5899 |
| 26 | and/4,14,24 | 2476 |
| 27 | (4 or 11) and 15 and 16 and 24 | 3492 |
| 28 | or/25-27 | 7735 |
| 29 | (animal/ or animal experiment/ or animal model/ or nonhuman/) not human/ | 7546996 |
| 30 | 28 not 29 | 7669 |
