# Supplementary material for: Effectiveness and safety of chronic diuretic use in older adults: an umbrella review of recently published systematic reviews and meta-analyses of randomized-controlled trials
Source: Eur Geriatr Med. 2025 May 25;16(4):1353–87. doi: 10.1007/s41999-025-01229-5 (PMC12378697; doi:10.1007/s41999-025-01229-5)
Supplement: Supplementary file 2 — Supplementary file2 (DOCX 25 KB) [file 41999_2025_1229_MOESM2_ESM.docx]

**Table 1.** PICOS table.

| Population | Include: adults (18 years or older).  Exclude: children (≤18 years old). |
| --- | --- |
| Interventions, exposures | **Include:** diuretics defined by WHO ATC code for class diuretics (C03AA01 bendroflumethiazide; C03AA02 hydroflumethiazide; C03AA03 hydrochlorothiazide; C03AA04 chlorothiazide; C03AA05 polythiazide; C03AA06 trichlormethiazide; C03AA07 cyclopenthiazide; C03AA08 methyclothiazide; C03AA09 cyclothiazide; C03AA13 mebutizide; C03BA02 quinethazone; C03BA03 clopamide; C03BA04 chlortalidone; C03BA05 mefruside; C03BA07 clofenamide; C03BA08 metolazone; C03BA09 meticrane; C03BA10 xipamide; C03BA11 indapamide; C03BA12 clorexolone; C03BA13 fenquizone; C03BC01 mersalyl; C03BD01 theobromine; C03BX03 cicletanine; C03CA01 furosemide; C03CA02 bumetanide; C03CA03 piretanide; C03CA04 torasemide; C03CC01 etacrynic acid; C03CC02 tienilic acid; C03CD01 muzolimine; C03CX01 etozolin; C03DA01 spironolactone C03DA02 potassium canrenoate; C03DA03 canrenone; C03DA04 eplerenone; C03DA05 finerenone; C03DB01 amiloride; C03DB02 triamterene  Comparisons: diuretic vs no diuretic/placebo; diuretic subclass comparison.  **Exclude:** C03XA01 tolvaptan and C03XA02 conivaptan. |
| Context/setting | **Include:** community-dwelling, long-term care facilities, residential care, nursing homes.  **Exclude:** reviews on the effects of diuretic use in acute conditions (e.g. acute congestive heart failure) or temporary timeframes/conditions (e.g., perioperatively, during pregnancy). |
| Outcomes | **Include:** efficacy, safety and tolerability outcomes (e.g., mortality, quality of life, adverse drug reactions such as falls) expressed as Odds Ratio, Hazard Ratio, Risk Ratio, Mean Difference, Standardized Mean Difference or Weighted Mean Difference.  **Exclude:** cost-efficacy studies. |
| Study type | **Include:** peer-reviewed, quantitative systematic reviews and (network) meta-analyses in English language, reporting quantitatively on data from ≥2 randomized controlled trials (reporting OR, HR, RR, MD, SMD or WMD). The term “systematic” refers to explicit, reproducible methods for identification of primary research studies and critical assessment and synthesis of studies that meet the eligibility criteria.  **Exclude:** non-systematic (e.g., “conventional”, “narrative” or “scoping”) reviews or reviews that incorporate theoretical studies or text and opinion as their primary source of evidence, protocol papers, non-English full-text, records of studies which seem to be eligible but for which data are incomplete or the publication related to the record cannot be obtained; cost-efficacy studies. |

ATC: Anatomical Therapeutic Chemical; HR: Hazard Ratio; MD: Mean Difference; OR: Odds Ratio; RR: Risk Ratio; SMD: Standardized Mean Difference; WHO: World Health Organization; WMD: Weighted Mean Difference.
