# Supplementary material for: Effectiveness and safety of chronic diuretic use in older adults: an umbrella review of recently published systematic reviews and meta-analyses of randomized-controlled trials
Source: Eur Geriatr Med. 2025 May 25;16(4):1353–87. doi: 10.1007/s41999-025-01229-5 (PMC12378697; doi:10.1007/s41999-025-01229-5)
Supplement: Supplementary file 3 — Supplementary file3 (DOCX 56 KB) [file 41999_2025_1229_MOESM3_ESM.docx]

**Supplementary Table 2.** Risk of bias (RoB) assessment based on the standardized Joanna Briggs Institute (JBI) Critical Appraisal Checklist For Systematic Reviews And Research Syntheses.

| Review | Domain  1. | Domain  2. | Domain  3. | Domain  4. | Domain  5. | Domain  6. | Domain  7. | Domain  8. | Domain  9. | Domain  10. | Domain  11. | Overall RoB |
| --- | --- | --- | --- | --- | --- | --- | --- | --- | --- | --- | --- | --- |
| Abdelazeem 2022 | yes | yes | unclear | yes | no | unclear | unclear | yes | unclear | yes | yes | moderate |
| Abraham 2020 | yes | yes | yes | yes | yes | unclear | yes | yes | yes | yes | no | low |
| Ahmed 2023 | yes | yes | yes | yes | yes | yes | yes | yes | yes | yes | yes | low |
| Albasri 2021 | yes | yes | yes | yes | yes | yes | yes | yes | yes | yes | yes | low |
| Alexandre 2019 | yes | yes | yes | yes | yes | yes | yes | yes | yes | yes | yes | low |
| Alexandrou 2019 | yes | yes | yes | yes | yes | unclear | unclear | yes | yes | yes | yes | low |
| Al-Sadawi 2024 | yes | yes | yes | yes | yes | yes | yes | yes | unclear | yes | yes | low |
| Asiimwe 2021 | yes | yes | yes | yes | yes | no | no | yes | yes | n.a. | unclear | moderate |
| Bao 2022 | yes | yes | unclear | yes | yes | yes | yes | yes | no | yes | yes | low |
| Bazoukis 2018a | yes | yes | yes | yes | yes | yes | unclear | yes | yes | yes | yes | low |
| Bazoukis 2018b | yes | yes | yes | no | yes | unclear | unclear | yes | yes | no | no | moderate |
| Bidel 2023 | yes | yes | yes | yes | yes | unclear | unclear | unclear | unclear | yes | yes | moderate |
| Bonsu 2018 | yes | yes | yes | yes | yes | yes | yes | yes | yes | yes | yes | low |
| Boulmpou 2022 | yes | yes | yes | yes | yes | yes | yes | yes | yes | yes | yes | low |
| Chen 2024 | yes | yes | yes | yes | yes | yes | yes | yes | yes | yes | yes | low |
| Clark 2024 | yes | yes | yes | yes | yes | yes | yes | yes | yes | yes | yes | low |
| Desbiens 2022 | yes | yes | yes | yes | yes | yes | yes | yes | yes | yes | yes | low |
| Dineva 2019 | yes | yes | yes | yes | yes | yes | yes | yes | yes | yes | yes | low |
| Dineva 2020 | yes | yes | yes | yes | yes | no | unclear | unclear | no | no | no | high |
| Ding 2023 | yes | yes | yes | yes | yes | yes | yes | yes | yes | yes | unclear | low |
| Du 2023 | yes | yes | yes | yes | yes | yes | yes | yes | yes | yes | yes | low |
| Dutta 2022 | yes | yes | yes | yes | yes | yes | yes | yes | no | no | yes | low |
| Eid 2021 | yes | yes | yes | yes | yes | yes | yes | yes | yes | yes | yes | low |
| Elshahat 2024 | yes | yes | yes | yes | unclear | yes | yes | unclear | unclear | yes | yes | low |
| Faisal 2022 | no | no | unclear | yes | yes | unclear | yes | yes | unclear | yes | yes | moderate |
| Farmakis 2022 | yes | yes | yes | yes | yes | yes | yes | yes | yes | yes | yes | low |
| Fatima 2023 | yes | yes | yes | yes | yes | yes | yes | yes | yes | yes | yes | low |
| Fernandes 2018 | yes | yes | yes | yes | yes | unclear | unclear | yes | unclear | no | yes | moderate |
| Ferre 2022 | yes | yes | unclear | yes | yes | yes | yes | yes | yes | yes | yes | low |
| Frankenstein 2020 | yes | yes | yes | yes | yes | yes | yes | unclear | yes | yes | yes | low |
| Fu 2021 | yes | yes | yes | yes | yes | yes | yes | yes | yes | yes | yes | low |
| Fukuta 2019 | yes | yes | yes | yes | yes | yes | yes | yes | yes | yes | yes | low |
| Geng 2023 | yes | yes | yes | yes | yes | yes | yes | yes | yes | yes | yes | low |
| Ghosal 2023a | yes | yes | yes | no | yes | unclear | unclear | unclear | yes | yes | yes | moderate |
| Ghosal 2023b | yes | yes | yes | yes | yes | yes | no | yes | yes | yes | no | low |
| Gu 2024 | yes | yes | yes | yes | yes | yes | yes | yes | unclear | yes | yes | low |
| Hall 2020 | yes | yes | yes | yes | yes | yes | no | no | yes | no | no | moderate |
| Hansen 2020 | no | yes | yes | yes | yes | unclear | yes | yes | yes | yes | no | low |
| Harrington 2023 | yes | unclear | unclear | unclear | unclear | unclear | unclear | yes | no | yes | yes | high |
| Hasegawa 2021 | yes | yes | yes | yes | yes | yes | yes | yes | yes | yes | yes | low |
| Ho 2024 | yes | yes | yes | yes | yes | yes | yes | yes | unclear | yes | yes | low |
| Hu 2022 | yes | yes | yes | yes | yes | yes | yes | yes | yes | yes | yes | low |
| Jiang 2022 | yes | yes | yes | yes | yes | yes | yes | yes | yes | yes | yes | low |
| Jyotsna 2023 | yes | yes | yes | yes | yes | yes | yes | yes | yes | yes | yes | low |
| Kapelios 2019 | yes | yes | yes | yes | yes | yes | yes | yes | yes | yes | yes | low |
| Karakasis 2024 | yes | yes | yes | yes | yes | yes | yes | yes | yes | yes | yes | low |
| Kido 2019 | yes | yes | yes | yes | yes | yes | yes | yes | yes | yes | yes | low |
| Kohjimoto 2024 | yes | yes | yes | unclear | yes | yes | unclear | yes | yes | yes | yes | low |
| Li 2018 | yes | yes | yes | yes | yes | yes | yes | yes | unclear | yes | yes | low |
| Li 2020 | yes | yes | yes | yes | yes | yes | yes | yes | yes | yes | yes | low |
| Li 2022 | yes | yes | yes | yes | yes | yes | yes | yes | yes | no | no | low |
| Li 2024 | yes | yes | yes | unclear | yes | yes | unclear | yes | yes | yes | unclear | low |
| Liu 2022 | yes | yes | yes | yes | yes | yes | yes | yes | yes | yes | yes | low |
| Lunney 2020 | yes | yes | yes | yes | yes | yes | yes | yes | yes | yes | yes | low |
| Ma 2021 | yes | yes | yes | yes | yes | yes | yes | yes | yes | yes | no | low |
| Macfarlane 2019 | yes | yes | unclear | yes | yes | yes | yes | yes | yes | yes | yes | low |
| Martin 2021 | yes | yes | yes | yes | yes | yes | yes | yes | yes | yes | yes | low |
| Martins 2023 | yes | yes | yes | yes | yes | yes | yes | yes | yes | yes | unclear | low |
| Miles 2019 | yes | yes | yes | no | yes | unclear | unclear | yes | yes | yes | yes | low |
| Morita 2023 | yes | yes | yes | yes | yes | yes | yes | yes | yes | yes | n.a. | low |
| Musini 2019 | yes | yes | yes | yes | yes | yes | yes | yes | yes | yes | yes | low |
| Nguyen 2023 | yes | yes | unclear | yes | unclear | yes | yes | yes | yes | yes | yes | low |
| Noone 2020 | yes | yes | yes | yes | unclear | yes | yes | unclear | unclear | yes | yes | low |
| Oraii 2024 | yes | yes | yes | yes | yes | yes | yes | yes | yes | yes | yes | low |
| Pamporis 2024 | yes | yes | yes | yes | yes | yes | yes | yes | yes | yes | yes | low |
| Patoulias 2021 | yes | unclear | yes | no | unclear | unclear | unclear | yes | unclear | yes | no | high |
| Patoulias 2022 | yes | yes | yes | yes | yes | yes | yes | yes | yes | yes | yes | low |
| Peters 2019 | yes | yes | yes | yes | unclear | yes | yes | yes | yes | yes | yes | low |
| Sakima 2021 | yes | yes | yes | yes | yes | yes | yes | yes | yes | yes | yes | low |
| Sampaio Rodrigues 2024 | yes | yes | yes | yes | yes | yes | yes | yes | yes | yes | yes | low |
| Seeley 2020 | yes | yes | yes | yes | yes | yes | yes | yes | yes | yes | yes | low |
| Shah 2018 | yes | unclear | unclear | unclear | unclear | unclear | unclear | yes | unclear | yes | yes | high |
| Shaman 2020 | yes | yes | yes | yes | yes | yes | yes | yes | yes | yes | yes | low |
| Sherif 2020 | yes | yes | yes | yes | yes | yes | yes | yes | yes | yes | yes | low |
| Shi 2023 | yes | yes | yes | yes | yes | yes | yes | yes | yes | yes | yes | low |
| Siddiqi 2023 | yes | yes | yes | yes | yes | yes | yes | yes | yes | yes | yes | low |
| Singh 2023 | yes | yes | yes | yes | yes | yes | yes | yes | yes | yes | yes | low |
| Sreenivasan 2022 | yes | yes | yes | yes | yes | yes | yes | yes | yes | yes | yes | low |
| Sreenivasan 2024 | yes | yes | yes | yes | yes | yes | yes | yes | yes | yes | yes | low |
| Täger 2019 | yes | yes | yes | yes | yes | yes | yes | unclear | yes | yes | yes | low |
| Teixeira 2024 | yes | yes | yes | yes | yes | yes | yes | yes | yes | yes | yes | low |
| Teles 2023 | yes | yes | yes | yes | yes | yes | yes | yes | no | yes | yes | low |
| Thomopoulos 2018 | yes | unclear | unclear | unclear | unclear | yes | yes | yes | unclear | yes | yes | moderate |
| Tian 2024 | yes | yes | yes | yes | yes | yes | yes | yes | yes | yes | yes | low |
| Tsukamoto 2022 | yes | yes | yes | yes | yes | yes | yes | yes | yes | yes | yes | low |
| Wang 2022 | yes | yes | yes | yes | yes | yes | unclear | yes | yes | yes | yes | low |
| Wei 2020 | yes | yes | yes | yes | yes | yes | yes | yes | yes | yes | yes | low |
| Wright 2018 | yes | yes | yes | yes | yes | yes | yes | yes | yes | yes | yes | low |
| Wu 2022 | yes | yes | yes | yes | yes | yes | yes | yes | yes | yes | yes | low |
| Xiang 2019 | yes | yes | yes | yes | yes | yes | yes | yes | yes | yes | yes | low |
| Xiang 2022 | yes | yes | yes | yes | unclear | yes | yes | yes | yes | yes | yes | low |
| Xie 2018 | yes | yes | yes | yes | yes | yes | yes | yes | yes | yes | yes | low |
| Xu 2018 | yes | yes | yes | yes | yes | yes | yes | yes | no | n.a. | yes | low |
| Xu 2024 | yes | yes | yes | yes | yes | yes | yes | yes | unclear | yes | yes | low |
| Yanai 2021 | yes | yes | yes | no | no | no | no | yes | yes | yes | yes | moderate |
| Yang 2019 | yes | yes | yes | yes | yes | yes | yes | unclear | yes | yes | yes | low |
| Yang 2022 | yes | yes | yes | yes | yes | yes | yes | yes | yes | yes | yes | low |
| Yang 2023a | yes | yes | yes | yes | yes | yes | yes | yes | n.a. | yes | yes | low |
| Yang 2023b | yes | yes | yes | yes | yes | yes | yes | yes | yes | yes | yes | low |
| Yang 2024 | yes | yes | yes | yes | yes | yes | yes | yes | unclear | yes | yes | low |
| Yasmin 2023 | yes | yes | yes | yes | yes | yes | yes | yes | yes | yes | yes | low |
| Yi 2024 | yes | yes | yes | yes | yes | yes | yes | yes | yes | yes | yes | low |
| Yuan 2024 | yes | yes | yes | yes | yes | yes | yes | unclear | yes | yes | unclear | low |
| Zafeiropoulos 2024 | yes | yes | yes | yes | yes | yes | yes | yes | yes | yes | yes | low |
| Zeng 2019 | yes | yes | yes | yes | yes | yes | yes | yes | yes | yes | yes | low |
| Zhang 2019 | yes | yes | yes | yes | yes | yes | yes | yes | yes | n.a. | yes | low |
| Zhang 2022a | yes | yes | yes | yes | yes | yes | yes | yes | yes | yes | yes | low |
| Zhang 2022b | yes | yes | yes | yes | yes | yes | yes | yes | yes | yes | yes | low |
| Zhang 2022c | yes | yes | yes | yes | yes | yes | yes | yes | yes | yes | yes | low |
| Zhao 2019 | yes | yes | yes | yes | yes | yes | yes | yes | unclear | n.a. | yes | low |
| Zheng 2018 | yes | yes | yes | yes | yes | yes | yes | yes | yes | n.a. | yes | low |
| Zheng 2022 | yes | yes | yes | yes | yes | yes | yes | yes | yes | yes | yes | low |
| Zhong 2021 | yes | yes | yes | yes | yes | yes | yes | yes | yes | yes | yes | low |
| Zhu 2020 | yes | yes | yes | yes | yes | yes | yes | yes | yes | n.a. | yes | low |
| Zhu 2021 | yes | yes | unclear | yes | yes | yes | yes | yes | yes | yes | yes | low |
| Zhu 2022 | yes | yes | yes | yes | yes | yes | yes | yes | yes | n.a. | yes | low |
| Zonneveld 2018 | yes | yes | yes | yes | yes | yes | yes | yes | unclear | yes | yes | low |

Domain 1: Is the review question clearly and explicitly stated?

Domain 2: Were the inclusion criteria appropriate for the review question?

Domain 3: Was the search strategy appropriate?

Domain 4: Were the sources and resources used to search for studies adequate?

Domain 5: Were the criteria for appraising studies appropriate?

Domain 6: Was critical appraisal conducted by two or more reviewers independently?

Domain 7: Were there methods to minimize errors in data extraction?

Domain 8: Were the methods used to combine studies appropriate?

Domain 9: Was the likelihood of publication bias assessed?

Domain 10: Were recommendations for policy and/or practice supported by the reported data?

Domain 11: Were the specific directives for new research appropriate?

*n.a.: not applicable*
