# Supplementary material for: Effectiveness and safety of chronic diuretic use in older adults: an umbrella review of recently published systematic reviews and meta-analyses of randomized-controlled trials
Source: Eur Geriatr Med. 2025 May 25;16(4):1353–87. doi: 10.1007/s41999-025-01229-5 (PMC12378697; doi:10.1007/s41999-025-01229-5)
Supplement: Supplementary file 4 — Supplementary file4 (DOCX 135 KB) [file 41999_2025_1229_MOESM4_ESM.docx]

**Table 3b.** Summary of findings table, narrative summary non-pooled data comparing diuretic to no diuretic therapy.

| **Review first author, year of publication** | **Outcome category** | **Specific outcome** | **Diuretic indication, population** | **Diuretic intervention** | **Com parison** | **Effect**  **(metric, 95% CI)** | **Narrative summaries non-pooled data**  **(number of effect estimates)** |
| --- | --- | --- | --- | --- | --- | --- | --- |
| Martins et al. 2023 | bio chemistry | plasma glucose | HT, primary | thiazide | placebo | MD 7.01 (4.21 to 9.80) | **Change in serum glucose and new-onset diabetes**  In patients with primary HT, serum glucose was higher with thiazides compared to placebo (n=1); fasting glucose was higher with thiazides compared to placebo (n=1); new-onset diabetes risk was comparable with diuretics and placebo, irrespective of age (younger vs older than 65 years; n=1) (1). |
| Hall et al. 2020 |  | plasma glucose, fasting | not specified |  |  | WMD 0.2 (0.15 to 0.25) |  |
| Zhang et al. 2019 |  | diabetes, new-onset | not specified, older adults | diuretics (nonspecified) |  | OR 1.4 (0.92 to 2.5) |  |
| Xiang et al. 2019 | bio chemistry | hyper calcemia | HFmrEF and HFpEF | spironolactone | control | OR 2.56 (1.54 to 4.27) | **Hypercalcemia**  In patients with HFmrEF or HFpEF, the odds of spironolactone were higher with spironolactone compared to control (n=1). |
| Yang et al. 2022 | bio chemistry | hyper kalemia | CKD and T2D | MRA, nonselective | control | OR 3.22 (1.43 to 7.66) | **Hyperkalemia**  In patients with CKD and T2D, the odds of hyperkalemia were higher with nonselective and nonsteroidal MRAs compared to control (both n=1); in patients with HFrEF, the odds of hyperkalemia were higher with eplerenone compared to no eplerenone and with spironolactone compared to no spironolactone (both n=1); in patients after MI, the risk of hyperkalemia was higher with MRAs compared to control (n=1); in patients with HT, the risk of hyperkalemia was higher with MRAs compared to placebo (n=1); the risk of hyperkalemia was comparable with MRAs compared to placebo or standard medical care (n=1). |
|  |  |  |  | MRA, nonsteroidal |  | OR 2.27 (2.02 to 2.56) |  |
| Pamporis et al. 2024 |  |  | HFrEF | eplerenone | no eplerenone | RR 1.88 (1.53 to 2.30) |  |
|  |  |  |  | spironolactone | no spironolactone | RR 2.44 (1.50 to 3.97) |  |
| Yi et al. 2024 |  |  | HT | MRA | placebo | OR 2.68 (1.99 to 3.62) |  |
| Sampaio Rodrigues et al. 2024 |  |  | not specified |  | placebo or standard medical care | OR 1.80 (0.93 to 3.48) |  |
| Xu et al. 2018 |  |  | post MI |  | control | RR 2.05 (1.6 to 2.61) |  |
| Harrington et al. 2023 | bio chemistry | hypo kalemia | HF | MRA | placebo | OR 0.4 (0.23 to 0.69) | **Hypokalemia**  In patients with HF, the odds of hypokalemia were lower for MRA compared to placebo (n=1); in patients with HT, the risk of hypokalemia with diuretics was comparable for diuretics compared to control (n=1). |
| Albasri et al. 2021 |  |  | HT | diuretics (nonspecified) | control | RR 10.73 (0.32 to 354.58) |  |
| Yang et al. 2022 | bio chemistry | hypo natremia | CKD and T2D | MRA, nonsteroidal | control | OR 16.56 (2.78 to 455.19) | **Hyponatremia**  In patients with CKD and T2D, the odds of hyponatremia were higher with nonsteroidal MRAs compared to control (n=1). |
| Chen et al. 2024 | bio chemistry | potassium, increase | CKD | MRA, nonsteroidal | placebo | RR 5.21 (2.57 to 10.56) | **Change in serum potassium**  In patients with CKD with or without T2D and in DKD, MRAs increased serum potassium compared to control (n=2), no MRA (n=1) or placebo (n=1); in patients with CKD, the risk of an increase in serum potassium was higher with nonsteroidal MRAs compared to placebo (n=1); in patients with ESKD requiring dialysis, effects on serum potassium were comparable for MRA compared to placebo (n=1) and spironolactone compared to control (n=1); in patients with HFpEF and/or HFmrEF, serum potassium was higher with MRA or spironolactone compared to control (both n=1); in patients with primary HT, serum potassium was lower with thiazides compared to placebo (n=1). |
| Yuan et al. 2023 |  | serum potassium |  | MRA | control | MD 0.18 (0.14 to 0.22) |  |
| Ding et al. 2023 |  |  |  | MRA, nonsteroidal | no nonsteroidal MRA | MD 0.16 (0.13 to 0.20) |  |
| Zhang et al. 2022_3 |  |  | CKD and T2D | finerenone | control | MD 0.17 (0.1 to 0.24) |  |
| Hu et al. 2022 |  |  | DKD | eplerenone | placebo | MD 0.15 (0.01 to 0.29) |  |
| Hasegawa et al. 2021 |  |  | ESKD requiring dialysis | MRA |  | MD 0.21 (-0.06 to 0.47) |  |
| Zeng et al. 2019 |  |  |  | spironolactone | control | MD 0 (-0.18 to 0.1) |  |
| Xiang et al. 2019 |  |  | HFmrEF and HFpEF |  |  | MD 0.25 (0.18 to 0.33) |  |
| Kapelios et al. 2019 |  |  | HFpEF | MRA |  | MD 0.23 (0.19 to 0.28) |  |
| Martins et al. 2023 |  |  | HT, primary | thiazide | placebo | MD -0.49 (-0.56 to -0.42) |  |
| Martins et al. 2023 | bio chemistry | uric acid | HT, primary | thiazide | placebo | MD 0.95 (0.73 to 1.17) | **Change in serum uric acid**  In patients with primary HT, effects on serum uric acid were comparable with thiazides compared to placebo (n=1). |
| Karakasis et al. 2024 | cardio vascular | atrial fibrillation | CVD | MRA | control | RR 0.75 (0.66 to 0.87) | **Effect on atrial fibrillation**  The risk of atrial fibrillation, but not the risk for recurrent atrial fibrillation was lower with MRA compared to control (both n=1); in patients with CVD, the risk of atrial fibrillation was lower with MRA compared to control (n=1); in patients with CKD and T2D, the risk of new-onset atrial fibrillation was lower with finerenone compared to placebo (n=1) but not compared to control (n=1); the odds of new-onset atrial fibrillation were lower for diuretics compared to control (n=1) and for spironolactone compared to control (n=1); the risk of new-onset atrial fibrillation was comparable with MRA, eplerenone and spironolactone (all n=1) compared to placebo; the risk of recurrent atrial fibrillation was lower for MRAs compared to control (n=1). MRAs may have favourable effects on atrial fibrillation, but irrespective of age (no significant influence in subgroup and meta-regression analyses (2)). |
| Fatima et al. 2023 |  |  | not specified |  |  | RR 0.77 (0.65 to 0.91) |  |
|  |  | atrial fibrillation, new onset |  |  |  | RR 0.84 (0.61 to 1.16) |  |
| Yang et al. 2023_1 |  |  | CKD and T2D | finerenone |  | RR 0.62 (0.19 to 2) |  |
| Zhang et al. 2022_1 |  |  |  |  | placebo | RR 0.79 (0.62 to 0.99) |  |
| Alexandre et al. 2019 |  |  | not specified | diuretic (nonspecified) | control | OR 0.37 (0.23 to 0.59) |  |
| Patoulias et al. 2021 |  |  |  | eplerenone | placebo | RR 0.83 (0.3 to 2.3) |  |
|  |  |  |  | MRA |  | RR 0.81 (0.62 to 1.05) |  |
| Alexandre et al. 2019 |  |  |  | spironolactone | control | OR 0.62 (0.45 to 0.86) |  |
| Patoulias et al. 2021 |  |  |  |  | placebo | RR 0.89 (0.6 to 1.33) |  |
| Fatima et al. 2023 |  | atrial fibrillation, recurrent |  | MRA | control | RR 0.73 (0.59 to 0.90) |  |
| Teles et al. 2023 | cardio vascular | BP, mean | CKD, advanced | thiazide(like) | no treatment, other diuretics, or antihyper tensives | MD -6.18 (-7.77 to -4.59) | **Effect on BP regulation**  In patients with advanced CKD, mean BP was lower with thiazide(like) diuretics compared to no treatment, other diuretics, or antihypertensives (n=1); in patients with uncontrolled HT, BP response rate was higher with low and high dose hydrochlorothiazide added to ARB compared to ARB therapy alone (both n=1), but the rate of BP target achievement was comparable with low and high dose hydrochlorothiazide added to ARB therapy compared to ARB therapy alone (both n=1). In persons with ESKD requiring dialysis, there were no age-related differences in BP effects of MRAs (3). A subgroup analysis of a systematic review among individuals in Sub-Saharan individuals with HT showed that the BP effects of diuretics were independent of age (4). |
| Ma et al. 2021 |  | BP, response rate | HT, uncontrolled | ARB/HCTZ high dose | ARB | RR 1.78 (1.63 to 1.95) |  |
|  |  |  |  | ARB/HCTZ low dose |  | RR 1.5 (1.42 to 1.59) |  |
|  |  | BP, target achievement |  | ARB/HCTZ high dose |  | RR 1.16 (0.7 to 1.9) |  |
|  |  |  |  | ARB/HCTZ low dose |  | RR 1.08 (0.72 to 1.64) |  |
| Thomopoulos et al. 2018 | cardio vascular | composite | age <65 years | diuretic (nonspecified) | control | RR 0.82 (0.7 to 0.96) | **CV composite outcomes**  both in patients younger and older than 65 years, the risk of a composite CV outcome was lower with diuretics compared to no diuretics (both n=1); the risk of composite CV outcomes was lower in patients with CKD and/or T2D when using MRAs compared to placebo (n=2) or control (n=1); in patients with CKD and T2D, the risk of composite CV outcomes was lower with MRAs on top of SGLT2is compared to SGLT2is alone (n=1). A Cochrane review showed that thiazides as first-line primary prevention in healthy adults ≥60 years old with moderate to severe HT may reduce all-cause, CV, cerebrovascular and coronary morbidity (5). |
|  |  |  | age >65 years |  |  | RR 0.77 (0.69 to 0.87) |  |
| Chen et al. 2024 |  |  | CKD | MRA, nonsteroidal | placebo | RR 0.88 (0.80 to 0.95) |  |
| Tsukamoto et al. 2022 |  |  | CKD and T2D | MRA |  | RR 0.88 (0.81 to 0.97) |  |
|  |  |  |  | SGLT2i/MRA | SGLT2i | RR 0.76 (0.6 to 0.96) |  |
| Patoulias et al. 2022 |  |  | T2D | finerenone | control | RR 0.87 (0.8 to 0.95) |  |
| Wright et al. 2018 | cardio vascular | coronary events | HT, first line therapy | thiazide, high-dose | placebo or no antihyper tensive treatment | RR 1.01 (0.85 to 1.2) | **Coronary events**  in patients with HT, the risk of coronary events with first-line low-dose, but not high-dose thiazides was lower compared to placebo or no antihypertensive treatment (both n=1). |
|  |  |  |  | thiazide, low-dose |  | RR 0.72 (0.61 to 0.84) |  |
| Bao et al. 2022 | cardio vascular | CV events | CKD and T2D | finerenone | placebo | RR 0.88 (0.8 to 0.96) | **CV events**  in patients with CKD and T2D, and in patients with advanced CKD (on or on dialysis), the risk of CV events was lower with finerenone (n=2) or steroidal MRAs (n=1) compared to placebo; the risk of CV events was lower with diuretics compared to no diuretics (n=1). |
| Abdelazeem et al. 2022 |  |  |  |  |  | RR 0.88 (0.80 to 0.96) |  |
| Yanai et al. 2021 |  |  | CKD, pre or on dialysis | MRA, steroidal |  | OR 0.60 (0.50 to 0.73) |  |
| Wei et al. 2020 |  |  | not specified | diuretic (nonspecified) |  | RR 0.73 (0.62 to 0.85) |  |
| Fu et al. 2021 | cardio vascular | CVD | CKD | finerenone | placebo | RR 0.92 (0.85 to 0.99) | **CVD**  in patients with CKD and/or T2D the risk of CVD was lower with finerenone compared to placebo (n=1) or control (n=1); in patients with ESKD requiring dialysis the risk of CVD was lower with MRA compared to placebo (n=1) and with spironolactone compared to control (n=1); the risk of CVD was lower with diuretics compared to no diuretics (n=1). An individual participant-level data meta-analysis showed that there may not be age-dependent effects of thiazides for the prevention of CVD (6). |
| Zhang et al. 2022_3 |  |  | CKD and T2D |  | control | RR 0.88 (0.8 to 0.95) |  |
| Hasegawa et al. 2021 |  |  | ESKD requiring dialysis | MRA | placebo | RR 0.38 (0.18 to 0.76) |  |
| Liu et al. 2022 |  |  |  | spironolactone | control | RR 0.54 (0.35 to 0.85) |  |
| Xie et al. 2018 |  |  | not specified | diuretics (nonspecified) | placebo | RR 0.77 (0.66 to 0.9) |  |
| Hu et al. 2022 | cardio vascular | DBP | DKD | eplerenone | placebo | MD -0.06 (-1.98 to 1.85) | **Change in DBP**  in patients with HFpEF (n=1), ESKD requiring dialysis (n=1), (non)resistant HT (n=3) and CKD (n=3), DBP was lower with MRAs compared to placebo or control; in patients with DKD, DBP was comparable with eplerenone and placebo (n=1); in patients with ESKD requiring dialysis, DBP with spironolactone was comparable (n=1) or lower (n=1) compared to control; in patients with a history of stroke or TIA, DBP was comparable with diuretics and control (n=1); in patients with uncontrolled HT, DBP was lower with low-dose and high-dose hydrochlorothiazide added to ARB therapy compared to ARB therapy alone (both n=1); in hypertensive patients in Sub-Saharan Africa, DBP was lower with diuretics compared to placebo (n=1); in patients with HT, DBP was lower with thiazides as first line therapy compared to placebo (n=1) and with thiazides compared to exercise therapy (n=1); in patients with resistant HT, office and 24hr DBP was lower with MRA or spironolactone compared to placebo (all n=1). |
| Shaman et al. 2020 |  |  | ESKD requiring dialysis | MRA |  | MD -3.7 (-7.5 to 0.1) |  |
| Liu et al. 2022 |  |  |  | spironolactone | control | MD -0.12 (-3.52 to 3.27) |  |
| Zeng et al. 2019 |  |  |  |  |  | MD -4.01 (-6.9 to -1.12) |  |
| Kapelios et al. 2019 |  |  | HFpEF | MRA |  | MD -2.91 (-4.15 to -1.67) |  |
| Zonneveld et al. 2018 |  |  | history of stroke or TIA | diuretic (nonspecified) |  | MD 0.01 (-0.22 to 0.24) |  |
| Bazoukis et al. 2018_1 |  |  | HT | MRA | placebo | MD -3.79 (-4.85 to -2.72) |  |
| Noone et al. 2020 |  |  |  | thiazide | exercise therapy | MD -4.53 (-7.11 to -1.98) |  |
| Wright et al. 2018 |  |  | HT, first line therapy |  | placebo | MD -5.71 (-5.99 to -5.42) |  |
| Bazoukis et al. 2018_1 |  |  | HT, non-resistant | MRA |  | MD -4.04 (-5.39 to -2.7) |  |
|  |  |  | HT, resistant |  |  | MD -3.55 (-6.1 to -1) |  |
| Seeley et al. 2020 |  |  | HT, sub Saharan Africa | diuretic (nonspecified) |  | MD -7.94 (-14.6 to -1.27) |  |
| Ma et al. 2021 |  |  | HT, uncontrolled | ARB/HCTZ high dose | ARB | MD -4.16 (-4.75 to -3.58) |  |
|  |  |  |  | ARB/HCTZ low dose |  | MD -2.91 (-3.31 to -2.51) |  |
| Yuan et al. 2023 |  |  | CKD | MRA | control | MD -1.27 (-1.99 to -0.56) |  |
| Ding et al. 2023 |  |  |  |  | no MRA | MD -2.42 (-3.49 to -1.34) |  |
|  |  |  |  | MRA, nonsteroidal | no nonsteroidal MRA | MD -2.32 (-2.83 to -1.81) |  |
| Ahmed et al. 2023 |  | DBP, 24hr | HT, resistant | MRA | placebo | MD -10.56 (-12.82 to -8.30) |  |
| Tian et al. 2023 |  |  |  | spironolactone |  | MD -3.72 (-6.25 to -1.19) |  |
|  |  | DBP, office |  |  |  | MD -4.68 (-8.88 to -0.47) |  |
| Ahmed et al. 2023 |  |  |  | MRA |  | MD -11.97 (-16.41 to -7.54) |  |
| Yi et al. 2024 | cardio vascular | edema | HT | MRA | placebo | OR 0.64 (0.29 to 1.42) | **Edema**  in patients with HT, the odds of edema were comparable with MRA and placebo (n=1); in patients with CKD, the risk of edema was lower with MRA compared to control (n=1). |
| Yuan et al. 2023 |  |  | CKD |  | control | RR 0.65 (0.56 to 0.75) |  |
| Yasmin et al. 2023 | cardio vascular | HF | CKD | finerenone | placebo | OR 0.79 (0.68 to 0.92) | **HF-related outcomes**  in patients with CKD and an patients with CKD requiring dialysis, the risk of HF was lower with finerenone compared to placebo (both n=1); in patients with CKD and T2D, the risk of HF was comparable between finerenone and control (n=1); in patients with HFpEF, the odds of worsening of HF were comparable between MRA and placebo (n=1). |
| Yang et al. 2023_1 |  |  | CKD and T2D |  | control | RR 0.91 (0.74 to 1.12) |  |
| Zhu et al. 2021 |  |  | ESKD requiring dialysis |  |  | RR 0.78 (0.67 to 0.92) |  |
| Al-Sadawi et al. 2024 |  | HF, worsening | HFpEF | MRA | placebo | OR 0.53 (0.26 to 1.05) |  |
| Yang et al. 2023_1 | cardio vascular | HT, new-onset | CKD and T2D | finerenone | control | RR 0.71 (0.62 to 0.81) | **New-onset HT**  in patients with CKD and T2D, the risk of new-onset HT was lower with finerenone compared to control (n=1). |
| Zonneveld et al. 2018 | cardio vascular | MACE | history of stroke or TIA | diuretic (nonspecified) | control | RR 0.9 (0.78 to 1.04) | **MACE**  the risk of MACE in was lower with thiazides compared to placebo, both in men and women (both n=1); in patients with a history of stroke or TIA, MACE risk was comparable with diuretics compared to control (n=1). Meta-regression analysis in a systematic review of RCTs in individuals with HF, meta-regression analysis showed no age-related differences in effects of MRAs on MACE (7). |
| Bidel et al. 2023 |  | MACE, men | not specified | thiazide | placebo | HR 0.81 (0.74 to 0.87) |  |
|  |  | MACE, women |  |  |  | HR 0.78 (0.70 to 0.86) |  |
| Nguyen et al. 2023 | cardio vascular | MI | CKD and T2D | MRA, nonsteroidal | placebo | RR 0.9 (0.74 to 1.11) | **MI and revascularization**  the risk/odds of MI and revascularization was comparable with nonsteroidal MRAs compared to placebo or control in patients with CKD and/or T2D (n=3); in patients with a history of stroke or TIA, the risk was lower with diuretics compared to control (n=1); revascularization risk was lower with diuretics compared to placebo (n=1); in patients after MI, revascularization risk was comparable with MRAs and control (n=1). |
| Zonneveld et al. 2018 |  |  | history of stroke or TIA | diuretic (nonspecified) | control | RR 0.9 (0.72 to 1.11) |  |
| Yang et al. 2022 |  | MI, non-fatal | CKD and T2D | MRA, nonsteroidal |  | OR 0.92 (0.74 to 1.13) |  |
| Shi et al. 2023 |  |  | T2D |  | standard treatment | OR 0.91 (0.74 to 1.12) |  |
| Wei et al. 2020 |  | revasculari zation | not specified | diuretic (nonspecified) | placebo | RR 0.67 (0.47 to 0.95) |  |
| Xu et al. 2018 |  |  | post MI | MRA | control | RR 1.09 (0.79 to 1.5) |  |
| Liu et al. 2022 | cardio vascular | SBP | ESKD requiring dialysis | spironolactone | control | MD -4.61 (-10.78 to 1.56) | **Change in SBP**  in patients with HFpEF (n=1), (non)resistant HT (n=3) and CKD (n=3), SBP was lower with MRAs compared to placebo or control; in patients with diabetes and proteinuric kidney disease, SBP was lower with MRA added to SGLT2i therapy compared to SGLT2i therapy alone (n=1); in patients with a history of stroke or TIA, SBP was comparable with diuretics and control (n=1); in patients with ESKD requiring dialysis, SBP with spironolactone was comparable (n=1) or lower (n=1) compared to control; in patients with uncontrolled HT, SBP was lower with low-dose and high-dose hydrochlorothiazide added to ARB therapy compared to ARB therapy alone (both n=1); in hypertensive patients in Sub-Saharan Africa, SBP was lower with diuretics compared to placebo (n=1); in patients with HT, SBP was lower with thiazides as first line therapy compared to placebo (n=1) and with thiazides compared to exercise therapy (n=1); in patients with resistant HT, office and 24hr SBP was lower with MRA compared to placebo (both n=1); in patients with resistant HT, office SBP, but not 24hr SBP was lower with spironolactone compared to placebo (both n=1). |
| Zeng et al. 2019 |  |  |  |  |  | MD -6.97 (-10.56 to -3.37) |  |
| Kapelios et al. 2019 |  |  | HFpEF | MRA |  | MD -4.75 (-8.94 to -0.56) |  |
| Zonneveld et al. 2018 |  |  | history of stroke or TIA | diuretic (nonspecified) |  | MD -0.01 (-0.39 to 0.37) |  |
| Bazoukis et al. 2018_1 |  |  | HT | MRA | placebo | MD -9.7 (-12.78 to -6.61) |  |
| Noone et al. 2020 |  |  |  | thiazide | exercise therapy | MD -9.11 (-14.26 to -4.36) |  |
| Wright et al. 2018 |  |  | HT, first line therapy |  | placebo | MD -13.04 (-13.53 to -12.55) |  |
| Bazoukis et al. 2018_1 |  |  | HT, non-resistant | MRA |  | MD -9.11 (-12.87 to -5.36) |  |
|  |  |  | HT, resistant |  |  | MD -10.96 (-17.37 to -4.56) |  |
| Seeley et al. 2020 |  |  | HT, sub Saharan Africa | diuretic (nonspecified) |  | MD -14.37 (-26.44 to -2.29) |  |
| Ma et al. 2021 |  |  | HT, uncontrolled | ARB/HCTZ high dose | ARB | MD -9.1 (-11.78 to -6.42) |  |
|  |  |  |  | ARB/HCTZ low dose |  | MD -5.69 (-6.66 to -4.73) |  |
| Morita et al. 2022 |  |  | proteinuric kidney disease in diabetes | SGLT2i/MRA | SGLT2i | MD -5.8 (-9.53 to -2.06) |  |
| Yuan et al. 2023 |  |  | CKD | MRA | control | MD -3.88 (-5.11 to -2.65) |  |
| Ding et al. 2023 |  |  |  | MRA, nonsteroidal | no nonsteroidal MRA | MD -4.59 (-6.72 to -2.46) |  |
|  |  |  |  | MRA, steroidal | no steroidal MRA | MD -4.89 (-7.07 to -2.71) |  |
| Ahmed et al. 2023 |  | SBP, 24hr | HT, resistant | MRA | placebo | MD -5.48 (-8.48 to -2.58) |  |
| Tian et al. 2023 |  |  |  | spironolactone |  | MD -9.74 (-19.89 to 0.42) |  |
|  |  | SBP, office |  |  |  | MD -13.85 (-21.85 to -5.85) |  |
| Ahmed et al. 2023 |  |  |  | MRA |  | MD -4.14 (-5.62 to -2.65) |  |
| Geng et al. 2023 | cardio vascular | stroke | CHF | MRA | placebo | RR 1.10 (0.82 to 1.47) | **Stroke**  the odds/risk of stroke were lower with diuretics compared to placebo (n=2); in patients with CHF, stroke risk was comparable with MRAs and placebo (n=1); in patients with a history of stroke or TIA, stroke risk was lower with diuretics compared to control (n=1); in patients with HT, stroke risk was reduced with low-dose and high-dose thiazides as first line therapy compared to placebo or no antihypertensive treatment (both n=1); in patients with T2D and/or CKD, non-fatal stroke risk was comparable with nonsteroidal MRAs and control or standard treatment (both n=1). Results for potential age-related differences in stroke risk reduction showed inconsistent results: in on review, the risk was reduced in older, but not in younger (cut-off 65 years) adults (8), whereas in another review, the risk was reduced both in individuals older and younger than 60 years (9). |
| Wright et al. 2018 |  |  | HT, first line therapy | thiazide, high-dose | placebo or no antihyper tensive treatment | RR 0.47 (0.37 to 0.61) |  |
| Wright et al. 2018 |  |  |  | thiazide, low-dose |  | RR 0.68 (0.6 to 0.77) |  |
| Zhong et al. 2021 |  |  | not specified | diuretic (nonspecified) | placebo | OR 0.68 (0.59 to 0.77) |  |
| Wei et al. 2020 |  |  |  |  |  | RR 0.63 (0.53 to 0.76) |  |
| Yang et al. 2022 |  | stroke, non-fatal | CKD and T2D | MRA, nonsteroidal | control | OR 1.00 (0.82 to 1.22) |  |
| Shi et al. 2023 |  |  | T2D |  | standard treatment | OR 1.00 (0.82 to 1.22) |  |
| Zonneveld et al. 2018 |  | stroke, recurrent | history of stroke or TIA | diuretic (nonspecified) | control | RR 0.81 (0.7 to 0.93) |  |
| Peters et al. 2020 | cognition | cognitive decline | not specified | diuretic (nonspecified) | control | OR 0.81 (0.59 to 1.12) | **Cognitive decline and new-onset dementia**  the risk of a new dementia diagnosis was lower compared to control with diuretics in at least 1 year follow-up (n=1), but not in at least 5 years follow-up (n=1); in patients with a history of stroke or TIA, the risk of a new dementia diagnosis was comparable between diuretics and control (n=1); the risk of cognitive decline was comparable between diuretics and control (n=1). |
| Zonneveld et al. 2018 |  | dementia, new-onset | history of stroke or TIA |  |  | RR 0.88 (0.73 to 1.06) |  |
| Peters et al. 2020 |  |  | not specified, follow up >1 year |  |  | OR 0.83 (0.72 to 0.96) |  |
|  |  |  | not specified, follow-up >5 years |  |  | OR 0.84 (0.55 to 1.29) |  |
| Xiang et al. 2019 | functional | 6MWD | HFmrEF and HFpEF | spironolactone | control | SMD 0.45 (0.27 to 0.64) | **6MWD**  in patients with HFpEF, 6MWD was comparable with MRAs or spironolactone (n=3) or smaller (n=1) compared to control or placebo; in patients with HFmrEF or HFpEF, 6MWD was larger with spironolactone compared to control (n=1). |
| Kapelios et al. 2019 |  |  | HFpEF | MRA |  | MD -11.56 (-21 to -2.1) |  |
| Fernandes et al. 2018 |  |  |  |  | placebo | MD -7.46 (-15.52 to 0.6) |  |
| Li et al. 2018 |  |  |  | spironolactone | control | MD -10.84 (-28.47 to 6.8) |  |
| Boulmpou et al. 2022 |  |  |  |  | placebo | WMD 25.72 (-46.22 to 97.67) |  |
| Kapelios et al. 2019 | functional | peak VO_2_ | HFpEF | MRA | control | MD 1.22 (-0.33 to 2.77) | **Peak VO_2_ and VE/VCO_2_**  in patients with HFpEF, peak VO_2_ (n=2) and VE/VCO_2_ (n=1) were comparable with MRAs or spironolactone compared to placebo or control. |
| Boulmpou et al. 2022 |  |  |  | spironolactone | placebo | WMD 0.39 (-0.72 to 1.5) |  |
| Fernandes et al. 2018 |  | VE/VCO_2_ |  |  |  | MD 0.62 (-0.96 to 2.19) |  |
| Xiang et al. 2019 | heart bio markers | BNP | HFmrEF and HFpEF | spironolactone | control | MD -44.80 (-73.44 to -16.17 ) | **Heart biomarkers**  in patients with HFmrEF or HFpEF, serum BNP and fibrosis marker PICP were lower with spironolactone compared to control (both n=1); fibrosis marker PIIINR was lower compared to control only in patients with HFpEF (n=1); in patients with HFpEF, BNP levels and BNP/NT-proBNP ratio were comparable between MRAs and placebo or control (both n=1); in patients with CKD, NT-proBNP levels were comparable between nonsteroidal MRAs and placebo (n=1). |
| Fukuta et al. 2019 |  |  | HFpEF | MRA | placebo | WMD 5226 (-15416 to 25869) |  |
| Kapelios et al. 2019 |  | BNP/NT-proBNP |  |  | control | MD 0 (-0.29 to 0.28) |  |
| Chen et al. 2024 |  | NT-proBNP | CKD | MRA, nonsteroidal | placebo | WMD -278.89 (-638.03 to 80.24) |  |
| Xiang et al. 2019 |  | PICP (fibrosis marker) | HFmrEF and HFpEF | spironolactone | control | MD -27.04 (-40.77 to -13.32) |  |
|  |  | PIIINR (fibrosis marker) | HFpEF |  |  | SMD -0.37 (-0.15 to -0.59) |  |
| Sakima et al. 2021 | heart ultrasound | augmen tation index | not specified | MRA | control | MD -6.74 (-10.26 to -3.21) | **Heart ultrasound markers**  augmentation index was lower with MRAs compared to control (n=1); FMD was larger with MRAs compared to control (n=1); in patients with ESKD requiring dialysis, left ventricle mass was lower with MRAs compared to placebo (n=1), and LVEF was comparable with MRAs compared to placebo (n=1), while LVEF with spironolactone was comparable (n=1) or larger (n=1) compared to control, and LVMi was lower with spironolactone compared to control (n=2); PWV was lower with MRAs compared to control (n=1); in patients with CKD and T2D, LVEF was comparable with MRAs and control (n=1), in patients after MI, LVEF was higher with MRAs compared to control (n=1); in patients with HFmrEF and LVEF, HFpEF, E/A ratio and E/e' were comparable with spironolactone and control (all n=1), while LVEDD was higher with spironolactone compared to control (n=1); in patients with HFpEF, LVMi, LVEF, DT and E/A were comparable with MRAs compared to control (all n=1), while E' was larger (n=1) and E/e' (n=2) and LAVi (n=1) and LVEDD (n=1) were smaller with MRAs compared to placebo or control; in patients with HFpEF, DT (n=1), E/A (n=1) and LVEF (n=1) were comparable with spironolactone and control; in patients with HFpEF, E/e' was smaller with spironolactone compared to control or placebo (both n=1), while LVEF was comparable between spironolactone and placebo (n=1). MRAs may have favourable effects on augmentation index, flow-mediated dilation (FMD) and PWV, but subgroup analyses show this is irrespective of age (10). Spironolactone may have a favourable effect on LVEDD in individuals with HFmrEF or HFpEF, and on E/e’ in persons with HFpEF, but subgroup analysis showed no significant difference between older and younger (70 year cut-off) individuals (11). |
| Kapelios et al. 2019 |  | DT | HFpEF |  |  | MD -8.38 (-21.76 to 5.00) |  |
| Li et al. 2018 |  |  |  | spironolactone |  | MD 1.04 (-8.27 to 10.35) |  |
| Fukuta et al. 2019 |  | E' |  | MRA | placebo | WMD 0.455 (0.232 to 0.679) |  |
| Xiang et al. 2019 |  | E/A | HFmrEF and HFpEF | spironolactone | control | SMD 0.08 (-0.11 to 0.27) |  |
| Kapelios et al. 2019 |  |  | HFpEF | MRA |  | MD -0.04 (-0.08 to 0.0) |  |
| Li et al. 2018 |  |  |  | spironolactone |  | MD -0.05 (-0.1 to 0) |  |
| Xiang et al. 2019 |  | E/e' | HFmrEF and HFpEF |  |  | SMD -0.1 (-0.22 to 0.01) |  |
| Kapelios et al. 2019 |  |  | HFpEF | MRA |  | MD -1.37 (-1.02 to -1.72) |  |
| Fernandes et al. 2018 |  |  |  |  | placebo | MD -1.62 (-2.19 to -1.06) |  |
| Li et al. 2018 |  |  |  | spironolactone | control | MD -1.38 (-2.03 to -0.73) |  |
| Boulmpou et al. 2022 |  |  |  |  | placebo | WMD -1.64 (-2.42 to -0.86) |  |
| Sakima et al. 2021 |  | FMD | not specified | MRA | control | MD 1.18 (0.14 to 2.23) |  |
| Kapelios et al. 2019 |  | LAVI | HFpEF |  |  | MD -1.12 (-1.91 to -0.33) |  |
| Hasegawa et al. 2021 |  | left ventricle mass | ESKD requiring dialysis |  | placebo | SMD -0.42 (-0.78 to -0.05) |  |
| Xiang et al. 2019 |  | LVEDD | HFmrEF and HFpEF | spironolactone | control | SMD 0.03 (0.21 to 0.27) |  |
| Kapelios et al. 2019 |  |  | HFpEF | MRA |  | MD -0.78 (-1.34 to -0.22) |  |
| Yang et al. 2023_2 |  | LVEF | CKD and T2D |  |  | RR 0.92 (0.83 to 1.09) |  |
| Hasegawa et al. 2021 |  |  | ESKD requiring dialysis |  | placebo | MD 3.15 (-0.74 to 7.04) |  |
| Liu et al. 2022 |  |  |  | spironolactone | control | MD 2.63 (-0.03 to 5.29) |  |
| Zeng et al. 2019 |  |  |  |  |  | MD 4.91 (2.58 to 7.24) |  |
| Xiang et al. 2019 |  |  | HFmrEF and HFpEF |  |  | SMD 0.08 (-0.11 to 0.27) |  |
| Kapelios et al. 2019 |  |  | HFpEF | MRA |  | MD 0.62 (-0.65 to 1.88) |  |
| Fernandes et al. 2018 |  |  |  | spironolactone | placebo | MD 0.7 (-1.45 to 2.84) |  |
| Xu et al. 2018 |  |  | post MI | MRA | control | MD 3.33 (0.91 to 5.75) |  |
| Liu et al. 2022 |  | LVMI | ESKD requiring dialysis | spironolactone |  | MD -6.28 (-10.29 to -2.28) |  |
| Zeng et al. 2019 |  |  |  |  |  | MD -0.58 (-0.82 to -0.34) |  |
| Kapelios et al. 2019 |  |  | HFpEF | MRA |  | MD -0.12 (-0.50 to 0.27) |  |
| Sakima et al. 2021 |  | PWV | not specified |  |  | MD -0.75 (-1.12 to -0.39) |  |
| Zhu et al. 2022 | hospitali zation | all cause | CKD or diabetes | finerenone | control | RR 0.97 (0.93 to 1.01) | **All-cause hospitalization**  in patients with HFmrEF or HFpEF, but not in patients with HFpEF, the odds of all-cause hospitalization were lower with spironolactone compared to control (both n=1); in a network meta-analysis (Pamporis 2024), the hazard of all cause hospitalization in patients with HFrEF was lower with spironolactone compared to no spironolactone (n=1), but the hazard was comparable with canrenone and eplerenone compared with no treatment (both n=1); in patients with CKD with or without diabetes and in patients with DKD, the risk of all-cause hospitalization was comparable between finerenone and placebo or control (both n=1), and with MRAs compared to no MRAs (n=1). |
| Dutta et al. 2022 |  |  | DKD |  | placebo | RR 0.94 (0.88 to 1.01) |  |
| Xiang et al. 2019 |  |  | HFmrEF and HFpEF | spironolactone | control | OR 0.84 (0.73 to 0.95) |  |
| Li et al. 2018 |  |  | HFpEF |  |  | OR 1.00 (0.8 to 1.25) |  |
| Pamporis et al. 2024 |  |  | HFrEF | eplerenone | no eplerenone | HR 0.83 (0.69 to 1.00) |  |
|  |  |  |  | spironolactone | no spironolactone | HR 0.51 (0.26 to 0.98) |  |
|  |  |  |  | canrenone | no canrenone | HR 0.89 (0.53 to 1.48) |  |
| Ding et al. 2023 |  |  | CKD | MRA | no MRA | HR 0.96 (0.91 to 1.01) |  |
| Yang et al. 2019 | hospitali zation | CVD-related | CKD and T2D | finerenone | placebo | OR 0.78 (0.66 to 0.92) | **CVD-related hospitalization**  in patients with CKD and T2D, the odds of CVD-related hospitalization were lower with finerenone compared to placebo (n=1); in patients with HFpEF, the risk of CVD-related hospitalization was comparable for MRA and spironolactone compared to placebo (both n=1), while the risk was comparable with spironolactone compared to no spironolactone (n=1). |
| Al-Sadawi et al. 2024 |  |  | HFpEF | MRA |  | OR 0.84 (0.48 to 1.46) |  |
| Faisal et al. 2022 |  |  |  | spironolactone |  | RR 0.97 (0.63 to 1.51) |  |
| Pamporis et al. 2024 |  |  | HFrEF |  | no spironolactone | HR 0.56 (0.37 to 0.84) |  |
| Yang et al. 2022 | hospitali zation | HF-related | CKD and T2D | MRA, nonsteroidal | control | OR 0.78 (0.66 to 0.92) | **HF-related hospitalization**  in patients with T2D and CKD but not without CKD, nonsteroidal MRAs, the odds of HF-related hospitalization were lower compared to control or standard treatment (both n=1); in patients with HFpEF, the odds of HF-related hospitalization were lower with MRAs compared to placebo (n=1). |
| Sreenivasan et al. 2024 |  |  | HFpEF | MRA | placebo | OR 0.81 (0.66 to 0.98) |  |
| Shi et al. 2023 |  |  | T2D | MRA, nonsteroidal | standard treatment | OR 1.00 (0.82 to 1.22) |  |
| Fukuta et al. 2019 | hospitali zation | hyperkalemia-related | HFpEF | MRA | placebo | RR 7.00 (0.381 to 129.00) | **Hyperkalemia-related hospitalization**  in patients with T2D and/or CKD, the risk/odds of hyperkalemia-related hospitalization was higher with steroidal MRAs compared to standard treatment (n=1) and with finerenone compared to placebo (n=1); in patients with HFpEF, the risk of hyperkalemia-related hospitalization was comparable between MRAs and placebo (n=1). |
| Shi et al. 2023 |  |  | T2D | MRA, nonsteroidal | standard treatment | OR 5.92 (3.02 to 11.62) |  |
| Jyotsna et al. 2023 |  |  | CKD and T2D | finerenone | placebo | RR 5.94 (4.04 to 8.75) |  |
| Hu et al. 2022 | kidney | albuminuria, 24hr | DKD | eplerenone | placebo | MD -12.8 (-23.91 to -1.69) | **24hr albuminuria**  24hr albuminuria was lower with eplerenone compared to placebo in patients with DKD (n=1), with MRAs compared to placebo in patients with proteinuric kidney disease (n=1), and with MRAs added to SGLT2i therapy compared to SGLT2i therapy alone in patients with diabetes and proteinuric kidney disease (n=1). |
| Alexandrou et al. 2019 |  |  | proteinuric kidney disease | MRA |  | MD -32.47 (-41.1 to -23.85) |  |
| Morita et al. 2022 |  |  | proteinuric kidney disease in diabetes | SGLT2i/MRA | SGLT2i | MD -34.19 (-41.08 to -27.3) |  |
| Ferre et al. 2022 | kidney | calciuria, 24hr | idiopathic hypercalciuria | thiazide(like) | usual treatment | SMD -0.93 (-1.87 to 0.02) | **24hr calciuria**  in patients with idiopathic hypercalciuria, 24hr calcium excretion was comparable with thiazide(like) diuretics compared to usual treatment (n=1); in patients with kidney stones, 24hr calcium excretion was lower with thiazides compared to placebo (n=1). |
| Li et al. 2020 |  |  | kidney stones | thiazide | placebo | SMD -18.59 (-25.11 to -12.08) |  |
| Gu et al. 2024 | kidney | CKD, progression | T2D | finerenone | placebo | HR 0.78 (0.66 to 0.92) | **CKD progression**  in patients with T2D, the risk of CKD progression was lower with finerenone compared to placebo (n=1). |
| Yang et al. 2022 | kidney | composite | CKD and T2D | finerenone | control | OR 0.76 (0.66 to 0.88) | **Composite kidney outcome**  in patients with CKD and T2D, the odds/risk of developing a composite kidney outcome was lower with finerenone and MRAs compared to control (both n=1), and comparable for nonselective MRAs compared to control (n=1) and MRAs added to SGLT2i therapy compared to SGLT2i therapy alone (n=1). |
| Yang et al. 2023_2 |  |  |  | MRA |  | RR 0.77 (0.68 to 0.88) |  |
| Yang et al. 2022 |  |  |  | MRA, nonselective |  | OR 1.58 (0.54 to 4.84) |  |
| Tsukamoto et al. 2022 |  |  |  | SGLT2i/MRA | SGLT2i | RR 0.85 (0.75 to 1.43) |  |
| Teles et al. 2023 | kidney | GFR | CKD, advanced | thiazide(like) | no treatment, other diuretics or antihyper tensives | MD -2.62 (-3.78 to -1.45) | **GFR**  in patients with advanced CKD, GFR was lower with thiazide(like) diuretics compared to no treatment, other diuretics or antihypertensives (n=1). |
| Yuan et al. 2023 | kidney | eGFR | CKD | MRA | control | MD -1.99 (-3.28 to -0.70) | **Effect on eGFR**  in patients with CKD and/or T2D, eGFR was reduced with MRA compared to control (n=1), with nonsteroidal but not steroidal MRAs compared to no (nonsteroidal) MRAs (both n=1), and with finerenone compared to control (n=1); the risk/odds/hazard of an eGFR reduction of >40% (n=1) or > 57% were lower for MRA or finerenone compared to no MRA or control (both n=1); the risk/odds of an eGFR <15 was lower with finerenone compared to placebo (n=1) or compared to control (n=1); in patients with CKD and T2D, eGFR slope was comparable with MRAs and control (n=1). |
| Ding et al. 2023 |  |  |  | MRA, nonsteroidal | no nonsteroidal MRA | MD -2.50 (-2.88 to -2.12) |  |
|  |  |  |  | MRA, steroidal | no steroidal MRA | MD -1.03 (-2.56 to 0.50) |  |
| Zhang et al. 2022_3 |  |  | CKD and T2D | finerenone | control | MD -2.44 (-2.82 to -2.05) |  |
| Yang et al. 2022 |  | eGFR, <15 |  |  |  | OR 0.81 (0.67 to 0.99) |  |
| Jyotsna et al. 2023 |  |  |  |  | placebo | RR 0.82 (0.72 to 0.94) |  |
| Yuan et al. 2023 |  | eGFR, >40% reduction | CKD | MRA | control | RR 0.85 (0.78 to 0.92) |  |
| Ding et al. 2023 |  | eGFR, >57% reduction |  |  | no MRA | HR 0.71 (0.60 to 0.83) |  |
| Yang et al. 2022 |  |  | CKD and T2D | finerenone | control | OR 0.7 (0.59 to 0.82) |  |
|  |  | eGFR, slope |  | MRA |  | MD 1.31 (0.89 to 1.74) |  |
| Yuan et al. 2023 | kidney | ESKD | CKD | MRA | control | RR 0.87 (0.74 to 1.01) | **ESKD**  in patients withT2D and/or CKD, the odds/risk of ESKD with MRA, finerenone or nonsteroidal MRAs compared to control or standard treatment was lower (n=3) or comparable (n=1); in patients with DKD, the risk of ESKD was higher with nonsteroidal MRAs compared to placebo (n=1). |
| Yang et al. 2022 |  |  | CKD and T2D | finerenone |  | OR 0.8 (0.64 to 0.99) |  |
| Yang et al. 2024 |  |  | DKD | MRA, nonsteroidal | placebo | RR 1.25 (1.02 to 1.55) |  |
| Zhang et al. 2022_3 |  |  | CKD and T2D | finerenone | control | RR 0.8 (0.65 to 0.99) |  |
| Shi et al. 2023 |  |  | T2D | MRA, nonsteroidal | standard treatment | OR 0.83 (0.75 to 0.92) |  |
| Hu et al. 2022 | kidney | fibrosis (laminin) | DKD | eplerenone | placebo | MD -14.1 (-23.61 to -4.59) | **Kidney fibrosis**  in patients with DKD, kidney fibrosis (laminin) was lower with eplerenone compared to placebo (n=1). |
| Pamporis et al. 2024 | kidney | injury | HFrEF | eplerenone | no eplerenone | RR 0.91 (0.50 to 1.67) | **Kidney injury and kidney failure**  in patients with HFrEF, the risk of kidney injury was comparable with and without eplerenone (n=1) and with and without spironolactone (n=1); in patients with HF, the odds of kidney failure were comparable with MRA and placebo (n=1); in patients with CHF, the risk of acute kidney failure was comparable with canrenone, eplerenone and spironolactone compared to placebo or standard medical care (all n=1); in patients with CKD and T2D, the risk of kidney failure was comparable between finerenone and placebo (n=1); in patients with CKD, the risk of chronic failure was lower with MRAs compared to control (n=1); in patients with CKD with and without T2D, the risk of acute kidney failure was comparable between MRA and control (n=2). |
|  |  |  |  | spironolactone | no spironolactone | RR 1.96 (0.90 to 4.27) |  |
| Harrington et al. 2023 |  | kidney failure | HF | MRA | placebo | OR 0.93 (0.59 to 1.46) |  |
| Jyotsna et al. 2023 |  |  | CKD and T2D | finerenone |  | RR 0.91 (0.81 to 1.02) |  |
| Yuan et al. 2023 |  | kidney failure, acute | CKD | MRA | control | RR 0.94 (0.79 to 1.13) |  |
| Frankenstein et al. 2020 |  |  | CHF | canrenone | placebo or standard medical care | HR 2.97 (0.46 to 19.17) |  |
|  |  |  |  | eplerenone |  | HR 0.7 (0.05 to 9.78) |  |
|  |  |  |  | spironolactone |  | HR 1.51 (0.11 to 20.05) |  |
| Yang et al. 2023_2 |  |  | CKD and T2D | MRA | control | RR 0.94 (0.78 to 1.12) |  |
| Yuan et al. 2023 |  | kidney failure, chronic | CKD |  |  | RR 0.86 (0.79 to 0.93) |  |
| Ferre 2022 | kidney | kidney stone formation rate | idiopathic hypercalciuria | thiazide | control | MD -0.16 (-0.30 to -0.03) | **Effects on kidney stones**  in patients with idiopathic hypercalciuria, thiazides increased the likeliness of being kidney stone-free compared to control (n=1), and reduced the risk of kidney stone incidence (n=1); in patients with calcium oxalate stones, the risk of recurrence of kidny stones was lower with thiazides compared to no thiazides (n=1); in patients with a history of kidney stones, the risk of recurrent kidney stones was lower with thiazides compared to placebo (n=1). |
| Kohjimoto et al. 2024 |  | kidney stone recurrence | calcium oxalate stones |  | no thiazides | RR 0.48 (0.37 to 0.62) |  |
| Ferre 2022 |  | kidney stone-free | idiopathic hypercalciuria |  | control | RR 1.64 (1.27 to 2.13) |  |
| Li et al. 2020 |  | kidney stones, incidence | kidney stones |  | placebo | RR 0.44 (0.33 to 0.58) |  |
| Ferre et al. 2022 |  | kidney stones, number of stone-free patients | idiopathic hypercalciuria | thiazide(like) | usual treatment | RR 1.61 (1.33 to 1.96) |  |
| Yuan et al. 2023 | kidney | proteinuria (24hr) | CKD | MRA | control | MD -0.20 (-0.28 to -0.12) | **24hr proteinuria**  in patients with CKD, 24hr proteinuria was lower with MRAs compared to control (n=1); in patients with DKD, 24hr proteinuria was lower with eplerenone compared to placebo (n=1). |
| Hu et al. 2022 |  |  | DKD | eplerenone | placebo | MD -37.6 (-73.3 to -1.9) |  |
| Hasegawa et al. 2021 | kidney | residual kidney function, urine volume/day | ESKD requiring dialysis | MRA | placebo | MD -36.23 (-114.61 to 42.15) | **Residual kidney function**  in patients with ESKD requiring dialysis, residual kidney function (urinary volume) was comparable with MRA compared to placebo (n=1). |
| Zhang et al. 2022_3 | kidney | UACR | CKD and T2D | finerenone | control | MD -0.3 (-0.32 to -0.28) | **UACR**  in patients with CKD and/or T2D and in patients with proteinuric kidney disease, UACR was lower with MRAs and finerenone (n=8 and n=1, respectively) compared to control or placebo. |
| Yang et al. 2023_2 |  |  | CKD and T2D, eGFR <45 | MRA |  | MD -31 (-33.6 to -28.5) |  |
|  |  |  | CKD and T2D, eGFR >45 |  |  | MD -46.8 (-15.7 to -79) |  |
|  |  |  | CKD and T2D, UACR <300 mg/g |  |  | MD -62.3 (-111 to -13) |  |
|  |  |  | CKD and T2D, UACR >300 mg/g |  |  | MD -31.52 (-33.28 to -29.73) |  |
| Alexandrou et al. 2019 |  |  | proteinuric kidney disease |  | placebo | MD -53.93 (-97 to -28.86) |  |
|  |  |  |  |  |  | SMD -0.67 (-1.24 to -0.11) |  |
| Morita et al. 2022 |  |  | proteinuric kidney disease in diabetes |  |  | MD -32.97 (-36.27 to -29.68) |  |
| Yuan et al. 2023 |  |  | CKD |  | control | MD -90.90 (-140.17 to -41.64) |  |
| Yang et al. 2022 | mortality | all cause | CKD and T2D | MRA, nonselective | control | OR 0.68 (0.08 to 4.68) | **All-cause mortality**  in patients with CKD and T2D, the odds of all cause mortality was comparable between nonselective (n=1) and nonsteroidal (n=1) MRAs; in patients with T2D, all cause mortality was comparable between nonsteroidal MRAs and standard medical treatment (n=1); in patients with ESKD requiring dialysis, all cause mortality was lower with spironolactone compared to control (n=1); in patients with HFmrEF or HFpEF, all cause mortality was comparable between spironolactone and control; in patients with HFpEF, all cause mortality was comparable between MRA (n=1) or spironolactone (n=1) and control; in patients with HFrEF, all cause mortality was comparable between azosemide (n=1), bumetanide (n=1), thiazides (n=1) and torasemide (n=1) compared to placebo; in patients with HFrEF, the risk of all cause mortality was comparable with furosemide compared to placebo (n=1), and the hazard ratio lower for eplerenone compared to no eplerenone (n=1) and for spironolactone compared to no spironolactone (n=1); in patients with a history of stroke or TIA, the risk of all cause mortality were comparable with diuretics compared to control (n=1), and lower with low-dose thiazides compared to placebo or no antihypertensive treatment (n=1); in patients with HT, the odds of all cause mortality were lower for MRAs compared to placebo (n=1), but comparable for thiazides compared to placebo (n=1); in patients with HT, all cause mortality risk was comparable with high-dose thiazides as 1st line therapy compared to placebo or no antihypertensive treatment (n=1); A subgroup analysis of a systematic review showed that in adults with HT, MRA use was associated with reduced risk of all-cause mortality in older, but not younger individuals (cut-off 65 years) (12); one review (8) reported CV mortality in older (≥65 years old), but not in younger adults (<65 years old). A Cochrane review showed that thiazides as first-line primary prevention in healthy adults ≥60 years old with moderate to severe HT may reduce all-cause, CV, cerebrovascular and coronary mortality (5); in patients with Covid-19, the odds of all cause mortality was comparable with and without diuretic treatment (n=1); in patients after MI, the risk of all cause mortality was comparable between canrenone (n=1) and spironolactone (n=1) compared to control, whereas the risk of all cause mortality was lower with MRAs (n=1) and eplerenone (n=1) compared to control; in patients with HF, MRAs postponed all cause mortality compared to placebo (n=1). |
|  |  |  |  | MRA, nonsteroidal |  | OR 0.89 (0.79 to 1) |  |
| Zeng et al. 2019 |  |  | ESKD requiring dialysis | spironolactone |  | OR 0.4 (0.24 to 0.66) |  |
| Xiang et al. 2019 |  |  | HFmrEF and HFpEF |  |  | RR 0.72 (0.31 to 1.69) |  |
| Zheng et al. 2018 |  |  | HFpEF | MRA |  | RR 0.92 (0.79 to 1.08) |  |
| Li et al. 2018 |  |  |  | spironolactone |  | OR 0.91 (0.76 to 1.1) |  |
| Täger et al. 2019 |  |  | HFrEF | azosemide | placebo | RR 0.98 (0.17 to 5.88) |  |
|  |  |  |  | bumetanide |  | RR 2.94 (0.32 to 25.00) |  |
| Pamporis et al. 2024 |  |  |  | eplerenone | no eplerenone | HR 0.78 (0.66 to 0.91) |  |
| Täger et al. 2019 |  |  |  | furosemide | placebo | RR 1.00 (0.20 to 5.00) |  |
| Pamporis et al. 2024 |  |  |  | spironolactone | no spironolactone | HR 0.79 (0.65 to 0.97) |  |
| Täger et al. 2019 |  |  |  | thiazide | placebo | RR 1.04 (0.14 to 8.03) |  |
|  |  |  |  | torasemide |  | RR 0.81 (0.15 to 4.27) |  |
| Zonneveld et al. 2018 |  |  | history of stroke or TIA | diuretics (nonspecified) | control | RR 0.98 (0.91 to 1.05) |  |
| Wright et al. 2018 |  |  |  | thiazide, low-dose | placebo or no antihyper tensive treatment | RR 0.89 (0.82 to 0.97) |  |
| Yi et al. 2024 |  |  | HT | MRA | placebo | OR 0.83 (0.74 to 0.92) |  |
|  |  |  |  | thiazide |  | OR 1.00 (0.89 to 1.12) |  |
| Wright et al. 2018 |  |  | HT, first line therapy | thiazide, high-dose | placebo or no antihyper tensive treatment | RR 0.9 (0.76 to 1.05) |  |
| Asiimwe et al. 2021 |  |  | not specified, patients with Covid-19 | diuretic (nonspecified) | no diuretic | OR 1.01 (0.59 to 1.74) |  |
| Xu et al. 2018 |  |  | post MI | canrenone | control | RR 0.66 (0.4 to 1.1) |  |
|  |  |  |  | eplerenone |  | RR 0.87 (0.78 to 0.97) |  |
|  |  |  |  | MRA |  | RR 0.84 (0.76 to 0.94) |  |
|  |  |  |  | spironolactone |  | RR 0.72 (0.4 to 1.31) |  |
| Shi et al. 2023 |  |  | T2D | MRA, nonsteroidal | standard treatment | OR 0.89 (0.79 to 1.00) |  |
| Hansen et al. 2020 |  | all cause (post ponement) | HF | MRA | placebo | SMD 41.3 (14.3 to 68.4) |  |
| Thomopoulos et al. 2018 | mortality | CV | age <65 years | diuretic (nonspecified) | control | RR 0.85 (0.74 to 0.98) | **CV mortality**  the risk of CV mortality was lower with diuretics compared to placebo (n=1); in patients <65 years, but not in patients >65 years, the risk of CV mortality was reduced with diuretics compared to control; in patients with HFpEF, the odds of CV mortality were comparable with MRAs and placebo (n=1); in patients with ESKD requiring dialysis, the risk/odds of CV mortality with MRAs or spironolactone was lower compared to placebo or control (all n=1); in patients with T2D and in patients with CKD or diabetes, the risk/odds of CV mortality with finerenone and nonsteroidal MRAs was comparable with control or standard treatment (all n=1); in patients with a history of MI, CV mortality risk was lower with MRA and with eplerenone (both n=1), but comparable for canrenone (n=1) compared to control; in patients with HT, CV mortality risk was lower with MRAs, but not with thiazides compared to placebo (both n=1). One review (8) reported CV mortality in older (≥65 years old), but not in younger adults (<65 years old). |
|  |  |  | age >65 years |  |  | RR 0.93 (0.67 to 1.31) |  |
| Zhu et al. 2022 |  |  | CKD or diabetes | finerenone |  | RR 0.88 (0.76 to 1.01) |  |
| Zhu et al. 2021 |  |  | ESKD requiring dialysis | MRA |  | RR 0.46 (0.28 to 0.76) |  |
| Nguyen et al. 2023 |  |  | CKD and T2D | MRA, nonsteroidal | placebo | RR 0.88 (0.76 to 1.02) |  |
| Zeng et al. 2019 |  |  | ESKD requiring dialysis | spironolactone | control | OR 0.4 (0.22 to 0.72) |  |
| Sreenivasan et al. 2024 |  |  | HFpEF | MRA | placebo | OR 0.90 (0.70 to 1.14) |  |
| Yi et al. 2024 |  |  | HT |  |  | OR 0.79 (0.68 to 0.93) |  |
|  |  |  |  | thiazide |  | OR 0.94 (0.66 to 1.35) |  |
| Wei et al. 2020 |  |  | not specified | diuretic (nonspecified) |  | RR 0.78 (0.69 to 0.88) |  |
| Xu et al. 2018 |  |  | post MI | canrenone | control | RR 0.7 (0.41 to 1.18) |  |
|  |  |  |  | eplerenone |  | RR 0.85 (0.75 to 0.96) |  |
|  |  |  |  | MRA |  | RR 0.84 (0.74 to 0.94) |  |
| Hasegawa et al. 2021 |  |  | ESKD requiring dialysis |  | placebo | RR 0.37 (0.22 to 0.64) |  |
| Shi et al. 2023 |  |  | T2D | MRA, nonsteroidal | standard treatment | OR 0.88 (0.75 to 1.02) |  |
| Xu et al. 2018 | mortality | HF-related | post MI | MRA | control | RR 0.78 (0.61 to 0.99) | **HF-related mortality**  in patients after MI, the risk of HF-related mortality was lower with MRAs compared to control (n=1). |
| Jyotsna et al. 2023 | mortality | kidney-related | T2D and CKD | finerenone | placebo | RR 0.56 (0.17 to 1.82) | **Kidney-related mortality**  in patients with T2D and CKD, the risk of kidney-related mortality was comparable between finerenone and control (n=1). |
| Peters et al. 2020 | mortality | mortality or drop-out | not specified | diuretic (nonspecified) | control | OR 0.95 (0.79 to 1.13) | **Mortality or drop-out**  the risk of drop-out or mortality was comparable between diuretics and control (n=1). |
| Zonneveld et al. 2018 | mortality | vascular | history of stroke or TIA | diuretics (nonspecified) | control | RR 0.85 (0.76 to 0.95) | **Vascular mortality**  in patients with a history of stroke or TIA, vascular mortality was lower with diuretics compared to control (n=1). |
| Martin et al. 2021 | quality of life | HRQoL, KCCQ score | HFpEF | MRA | placebo | MD -0.78 (-28.02 to 26.46) | **Quality of life**  in patients with HFpEF, quality of life (KCCQ, QoL score or MLHFQ score) was comparable for MRAs or spironolactone compared to placebo (n=4 and n=1, respectively). |
|  |  | HRQoL, MLHFQ score |  |  |  | MD 0.84 (-2.3 to 3.98) |  |
| Boulmpou et al. 2022 |  |  |  | spironolactone |  | WMD 0.75 (0.02 to 1.4) |  |
| Fukuta et al. 2019 |  | QoL score |  | MRA |  | SMD -0.018 (-0.184 to 0.147) |  |
| Martin et al. 2021 |  |  |  |  |  | SMD 0.05 (-0.23 to 0.34) |  |
| Zhong et al. 2021 | quality of life | tolerability | not specified | diuretic (nonspecified) | placebo | OR 1.86 (0.56 to 4.59) | **Tolerability**  tolerability was comparable for diuretics compared to placebo (n=1). |
| Thomopoulos et al. 2018 | various AEs | any AE | age <65 years | diuretic (nonspecified) | control | RR 1.88 (0.69 to 5.17) | **Any AE**  in patients >65 years, but not in patients <65 years, the risk of AEs was higher with diuretics compared to control (both n=1); in patients with calcium oxalate stones, the risk of AEs was higher with thiazides compared to no thiazides (n=1); in patients with HF, the odds of AEs was comparable with MRAs compared to placebo (n=1); in patients with HFrEF, the risk of AEs was comparable between eplerenone and no eplerenone (n=1), but higher for spironolactone compared to no spironolactone (n=1); in patients with uncontrolled HT, the risk of AEs was increased with high dose (n=1), but not with low-dose thiazides (n=1) on top of ARB compared to ARB therapy alone. In older (≥65 years), but not in younger (<65 years) adults, the risk of AEs associated with diuretic use may be increased (RR 1.88 (0.69 to 5.17) and RR 2.6 (1.38 to 4.88), respectively) (8). |
|  |  |  | age >65 years |  |  | RR 2.6 (1.38 to 4.88) |  |
| Kohjimoto et al. 2024 |  |  | calcium oxalate stones | thiazide | no thiazides | RR 2.95 (1.23 to 7.08) |  |
| Harrington et al. 2023 |  |  | HF | MRA | placebo | OR 0.99 (0.9 to 1.08) |  |
| Pamporis et al. 2024 |  |  | HFrEF | eplerenone | no eplerenone | RR 0.98 (0.92 to 1.04) |  |
|  |  |  |  | spironolactone | no spironolactone | RR 1.13 (1.02 to 1.26) |  |
| Ma et al. 2021 |  |  | HT, uncontrolled | ARB/HCTZ high dose | ARB | RR 1.17 (1.02 to 1.34) |  |
|  |  |  |  | ARB/HCTZ low dose |  | RR 1.01 (0.93 to 1.1) |  |
| Yuan et al. 2023 | various AEs | breast disorders | CKD | MRA | control | RR 1.39 (0.43 to 4.48) | **Breast disorders**  comparable risk for MRA vs control in patients with CKD (n=1); and breast pain: higher odds of MRAs vs placebo in patients with HF (n=1) |
| Harrington et al. 2023 |  | breast pain | HF |  | placebo | OR 10.38 (1.32 to 80.64) |  |
| Hu et al. 2022 | various AEs | cough | DKD | eplerenone | placebo | RR 1.02 (0.42 to 2.46) | **Cough**  comparable risk for eplerenone (n=1) and MRA vs placebo (n=1) |
| Harrington et al. 2023 |  |  | HF | MRA |  | OR 0.83 (0.7 to 2.59) |  |
| Kohjimoto et al. 2024 | various AEs | dis continuation | calcium oxalate stones | thiazide | no thiazides | RR 3.36 (0.70 to 16.19) | **Discontinuation**  In patients with calcium oxalate stones, the risk of drug discontinuation was comparable with thiazide compared to no thiazide use (n=1); in patients with CKD and T2D, the risk of discontinuation was higher with MRAs compared with control (n=1), but not for finerenone compared to placebo (n=1); in patients with ESKD, the risk of discontinuation was higher with MRAs compared to placebo (n=1); in patients with HF, the odds of discontinuation were comparable with MRA compared to placebo (n=1); in patients with HFpEF, the risk of discontinuation was comparable for MRAs compared to placebo (n=1); in patients with HFrEF, the risk of discontinuation was comparable for eplerenone compared to no eplerenone (n=1), but higher for spironolactone compared to no spironolactone (n=1); in patients with uncontrolled HT, the risk of discontinuation was comparable for hydrochlorothiazide on top of ARB therapy (both low-dose (n=1) and high-dose (n=1)); in patients with HT, the odds of or risk of discontinuation was comparable for MRAs compared to placebo (n=2) or control (n=1). |
| Yang et al. 2023_2 |  |  | CKD and T2D | MRA | control | RR 1.21 (1.05 to 1.38) |  |
| Shaman et al. 2020 |  |  | ESKD requiring dialysis |  | placebo | RR 4.05 (1.16 to 14.2) |  |
| Harrington et al. 2023 |  |  | HF |  |  | OR 0.89 (0.27 to 2.89) |  |
| Martin et al. 2021 |  |  | HFpEF |  |  | RR 1.1 (1 to 1.21) |  |
| Pamporis et al. 2024 |  |  | HFrEF | eplerenone | no eplerenone | RR 1.04 (0.95 to 1.13) |  |
|  |  |  |  | spironolactone | no spironolactone | RR 1.80 (1.29 to 2.51) |  |
| Bazoukis et al. 2018_1 |  |  | HT | MRA | control | RR 0.75 (0.52 to 1.09) |  |
| Yi et al. 2024 |  |  |  |  | placebo | OR 1.13 (0.76 to 1.68) |  |
| Bazoukis et al. 2018_1 |  |  |  |  |  | RR 1.93 (0.89 to 4.19) |  |
| Ma et al. 2021 |  |  | HT, uncontrolled | ARB/HCTZ high dose | ARB | RR 1.16 (0.7 to 1.9) |  |
|  |  |  |  | ARB/HCTZ low dose |  | RR 1.08 (0.72 to 1.64) |  |
| Jyotsna et al. 2023 |  |  | CKD and T2D | finerenone | placebo | RR 1.62 (0.84 to 3.10) |  |
| Yi et al. 2024 | various AEs | dizziness | HT | MRA | placebo | OR 1.01 (0.72 to 1.40) | **Dizziness**  in patients with HT, the odds of dizziness were comparable for MRAs compared to placebo (n=1). |
| Zhu et al. 2022 | various AEs | drug-related | CKD or diabetes | finerenone | control | RR 1.37 (1.27 to 1.48) | **Drug-related AEs**  in patients with CKD or diabetes, the risk of drug-related AEs was higher for finerenone compared to control (n=1); in patients with CKD and T2D, the risk of drug-related AEs was higher for finerenone compared to placebo (n=1); in patients with uncontrolled HT, the risk of drug-related AEs was higher with low-dose hydrochlorothiazide on top of ARB compared to ARB alone (n=1) and higher for high-dose compared to low-dose hydrochlorothiazide (n=1). |
| Ma et al. 2021 |  |  | HT, uncontrolled | ARB/HCTZ high dose | ARB/HCTZ low dose | RR 1.52 (1.01 to 2.29) |  |
|  |  |  |  | ARB/HCTZ low dose | ARB | RR 1.39 (1.12 to 1.72) |  |
| Jyotsna et al. 2023 |  |  | CKD and T2D | finerenone | placebo | RR 1.40 (1.33 to 1.46) |  |
| Farmakis et al. 2022 | various AEs | erectile dysfunction | not specified | thiazide | placebo | OR 0.23 (0.04 to 1.28) | **Erectile dysfunction**  the odds of erectile dysfunction were comparable with thiazides compared to placebo (n=1). |
| Desbiens et al. 2022 | various AEs | fracture, any site | not specified | thiazide(like) | placebo | RR 0.98 (0.8 to 1.2) | **Fractures**  the risk of any fracture was comparable with thiazide(like) diuretics and placebo (n=1); the risk of osteoporotic fractures was comparable for thiazides compared to placebo (n=1). Thiazide(like) diuretics may have a neutral effect on fracture risk, irrespective of age (13). |
|  |  | fractures, osteoporotic |  | thiazide |  | RR 0.71 (0.48 to 1.06) |  |
| Albasri et al. 2021 | various AEs | gout | HT | diuretic (nonspecified) | control | RR 4.48 (0.79 to 26.54) | **Gout**  in patients with HT, the risk of gout was comparable with diuretics compared control (n=1). |
| Dutta et al. 2022 | various AEs | gynecomastia | DKD | finerenone | placebo | RR 0.99 (0.63 to 1.57) | **Gynecomastia**  the odds of gynecomastia were higher with MRAs compared to placebo or standard medical care (n=1); in patients with DKD, the risk of gynecomastia was comparable with finerenone compared to placebo (n=1); in patients with ESKD requiring dialysis, the risk of gynecomastia was higher with MRA or spironolactone compared to control (both n=1), and higher with MRA compared to placebo (n=1); in patients with HFmrEF or HFpEF, the risk of gynecomastia was higher with spironolactone compared to control (n=1); in patients with HF, the risk of gynecomastia was higher with MRA compared to placebo (n=1); in patients with HFrEF, gynecomastia risk was higher with spironolactone compared to no spironolactone (n=1), but gynecomastia risk was comparable with eplerenone compared to no eplerenone (n=1). |
| Zhu et al. 2021 |  |  | ESKD requiring dialysis | MRA | control | RR 6.74 (2.86 to 15.9) |  |
| Hasegawa et al. 2021 |  |  |  |  | placebo | RR 5.95 (1.93 to 18.28) |  |
| Liu et al. 2022 |  |  |  | spironolactone | control | RR 4.36 (1.9 to 10.03) |  |
| Harrington et al. 2023 |  |  | HF | MRA | placebo | OR 7.61 (3.6 to 16.12) |  |
| Xiang et al. 2019 |  |  | HFmrEF and HFpEF | spironolactone | control | OR 7.82 (3.82 to 16.01) |  |
| Pamporis et al. 2024 |  |  | HFrEF | eplerenone | no eplerenone | RR 0.81 (0.47 to 1.41) |  |
|  |  |  |  | spironolactone | no spironolactone | RR 7.01 (3.57 to 13.79) |  |
| Sampaio Rodrigues et al. 2024 |  |  | not specified | MRA | placebo or standard medical care | OR 5.19 (1.26 to 21.34) |  |
| Zhang et al. 2022_2 | various AEs | HF-related AEs | CKD | finerenone | placebo | RR 0.79 (0.67 to 0.92) | **HF-related AEs**  in patients with CKD, the risk of HF-related AEs was lower with finerenone compared to placebo (n=1). |
| Bazoukis et al. 2018_1 | various AEs | hyper kalemia-related AEs | HT | MRA | placebo | RR 5.79 (1.67 to 20.03) | **Hyperkalemia-related AEs**  in patients with HT, the risk of hyperkalemia-related AEs was higher with MRAs compared to placebo (n=1). |
| Shi et al. 2023 | various AEs | hypoglycemia, severe | T2D | MRA, nonsteroidal | standard treatment | OR 0.64 (0.43 to 0.96) | **Severe hypoglycemia**  in patients with T2D, the odds of severe hypoglycemia were lower with nonsteroidal MRAs compared to standard treatment (n=1). |
| Harrington et al. 2023 | various AEs | hypotension | HF | MRA | placebo | OR 1.26 (0.81 to 1.95) | **Hypotension**  in patients with HF, the odds of hypotension were comparable with MRAs compared to placebo; in patients with HFrEF, the risk of hypotension was comparable with eplerenone compared to no eplerenoe (n=1); in patients with CKD, the risk of hypotension was higher with MRAs compared to control (n=1); in patients with ESKD requiring dialysis, the risk of hypotension was comparable with MRAs compared to control (n=1); in patients with HT the odds of hypotension were comparable with MRA compared to placebo (n=1). |
| Yuan et al. 2023 |  |  | CKD |  | control | RR 1.80 (1.41 to 2.31) |  |
| Shaman et al. 2020 |  |  | ESKD requiring dialysis |  | control | RR 1.16 (0.66 to 2.04) |  |
| Pamporis et al. 2024 |  |  | HFrEF | eplerenone | no eplerenone | RR 1.24 (0.86 to 1.79) |  |
| Yi et al. 2024 |  |  | HT | MRA | placebo | OR 1.90 (0.12 to 7.86) |  |
| Harrington et al. 2023 | various AEs | musculo skeletal disorders | HF | MRA | placebo | OR 0.94 (0.8 to 1.11) | **Musculoskeletal AEs**  in patients with HF, the risk of musculoskeletal disorders was comparable with MRAs compared to placebo (n=1). |
| Du et al. 2024 | various AEs | neoplasm, any | CKD and T2D | finerenone | placebo | OR 0.97 (0.83 to 1.14) | **Neoplasms**  a meta-analysis and systematic review of RCTs in patients with T2D and CKD (Du et al 2024) showed that compared to placebo, the risk of any, benign, in situ or malignant neoplasms was comparable with placebo (all n=1), but the risk of malignant neoplasms of the urinary tract was higher was finerenone compared to placebo (n=1). |
|  |  | neoplasm, benign |  |  |  | OR 0.94 (0.50 to 1.80) |  |
|  |  | neoplasm,  in situ |  |  |  | OR 0.14 (0.01 to 2.17) |  |
|  |  | neoplasm, malignant |  |  |  | OR 1.03 (0.86 to 1.23) |  |
|  |  | neoplasm, malignant of urinary tract |  |  |  | OR 1.69 (1.07 to 2.67) |  |
| Yuan et al. 2023 | various AEs | reproductive system disorders | CKD | MRA | control | RR 1.23 (0.45 to 3.37) | **Reproductive disorders**  in patients with CKD, the risk of reproductive system disorders was comparable with MRA compared to control (n=1). |
| Zhang et al. 2022_1 | various AEs | serious AEs | CKD and T2D | finerenone | placebo | RR 0.94 (0.9 to 0.99) | **Serious AEs**  in patients with CKD and T2D, the risk of serious AEs was lower with finerenone compared to placebo (n=2); in patients with DKD, the risk of serious AEs was lower with finerenone compared to control (n=1); in patients with uncontrolled HT, the risk of serious AEs with low and high dose hydrochlorothiazide on top of ARB compared to ARB therapy alone was comparable (both n=1); in patients with idipathic hypercalciuria, the risk of serious AEs was comparable with thiazide(like) diuretics compared to usual treatment (n=1). |
| Dutta et al. 2022 |  |  | DKD |  | control | RR 0.91 (0.84 to 0.97) |  |
| Ma et al. 2021 |  |  | HT, uncontrolled | ARB/HCTZ high dose | ARB | RR 0.7 (0.35 to 1.4) |  |
|  |  |  |  | ARB/HCTZ low dose |  | RR 0.77 (0.47 to 1.27) |  |
| Ferre et al. 2022 |  |  | idiopathic hypercalciuria | thiazide(like) | usual treatment | RR 5.00 (0.6 to 41.88) |  |
| Jyotsna et al. 2023 |  |  | CKD and T2D | finerenone | placebo | RR 0.95 (0.92 to 0.97) |  |

6MWD: six minute walking distance; AE: adverse event; AKI: acute kidney injury; ARB: angiotensin receptor blocker; BNP: brain natriuretic peptide; BP: blood pressure; CI: confidence interval; CKD: chronic kidney disease; CV: cardiovascular; CVD: cardiovascular disease; DBP: diastolic blood pressure; DKD: diabetic kidney disease; DT: deceleration time; E/A: the ratio of peak velocity blood flow from left ventricular relaxation in early diastole (the E wave) to peak velocity flow in late diastole caused by atrial contraction (the A wave marker of the function of the left ventricle of the heart), a marker of the function of the left ventricle of the heart; E/e’: peak E-wave velocity divided by the peak e’ velocity to estimate left ventricular end-diastolic function; E’: proxy for LV diastolic function; eGFR: estimated glomerular filtration rate; ESKD: end-stage kidney disease; FMD: flow-mediated dilation; GFR: glomerular filtration rate; HCTZ: hydrochlorothiazide; HF: heart failure; HFmrEF: heart failure with mid-range ejection fraction; HFpEF: heart failure with preserved ejection fraction; HFrEF: heart failure with reduced ejection fraction; HR: hazard ratio; HRQoL: health-related quality of life; HT: hypertension; KCCQ: Kansas City Cardiopathy Questionnaire; LAVI: left atrial volume index to measure left atrial size; LVEDD: left ventricular end-diastolic diameter; LVEF: left ventricular ejection fraction; LVMi: left ventricular mass index; MACE: major adverse cardiovascular event; MD: mean difference; MI: myocardial infarction; MLHFQ: Minnesota Living with Heart Failure Questionnaire; MRA: mineralocorticoid antagonist; NT-proBNP: N-terminal pro b-type natriuretic peptide; NYHA: New York Heart Association; OR: odds ratio; peak VO2: the oxygen uptake at the maximal level of tolerated exercise; PICP: procollagen type I C-terminal propeptide; PIIINP: amino-terminal peptide of procollagen type-III; PWV: pulse wave velocity; QoL: quality of life; RR: risk ratio; SBP: systolic blood pressure; SGLT2i: sodium-glucose cotransporter-2 inhibitor; SMD: standardized mean difference; T2D: type 2 diabetes mellitus; TIA: transient ischemic attack; UACR: urinary albumin to creatinine ratio; VE/VCO_2_: the ratio of minute ventilation (VE) to carbon dioxide (CO_2_), ventilatory efficiency during exercise; WMD: weighted mean difference.

1. Zhang J, Tong A, Dai Y, Niu J, Yu F, Xu F. Comparative risk of new-onset diabetes mellitus for antihypertensive drugs in elderly: A Bayesian network meta-analysis. J Clin Hypertens (Greenwich). 2019;21(8):1082-90. <http://dx.doi.org/10.1111/jch.13598>.

2. Alexandre J, Dolladille C, Douesnel L, Font J, Dabrowski R, Shavit L, et al. Effects of Mineralocorticoid Receptor Antagonists on Atrial Fibrillation Occurrence: A Systematic Review, Meta-Analysis, and Meta-Regression to Identify Modifying Factors. J Am Heart Assoc. 2019;8(22) (no pagination). <http://dx.doi.org/10.1161/jaha.119.013267>.

3. Shaman AM, Smyth B, Arnott C, Palmer SC, Mihailidou AS, Jardine MJ, et al. Comparative Efficacy and Safety of BP-Lowering Pharmacotherapy in Patients Undergoing Maintenance Dialysis: A Network Meta-Analysis of Randomized, Controlled Trials. Clin J Am Soc Nephrol. 2020;15(8):1129-38. <http://dx.doi.org/10.2215/cjn.12201019>.

4. Seeley A, Prynn J, Perera R, Street R, Davis D, Etyang AO. Pharmacotherapy for hypertension in Sub-Saharan Africa: a systematic review and network meta-analysis. BMC Med. 2020;18(1):75. <http://dx.doi.org/10.1186/s12916-020-01530-z>.

5. Musini VM, Tejani AM, Bassett K, Puil L, Wright JM. Pharmacotherapy for hypertension in adults 60 years or older. Cochrane Database Syst Rev. 2019;6:CD000028. <http://dx.doi.org/10.1002/14651858.cd000028.pub3>.

6. Bidel Z, Nazarzadeh M, Canoy D, Copland E, Gerdts E, Woodward M, et al. Sex-Specific Effects of Blood Pressure Lowering Pharmacotherapy for the Prevention of Cardiovascular Disease: An Individual Participant-Level Data Meta-Analysis. Hypertension. 2023;80(11):2293-302. <https://doi.org/10.1161/hypertensionaha.123.21496>.

7. Geng C, Mao YC, Qi SF, Song K, Wang HF, Zhang ZY, et al. Mineralocorticoid receptor antagonists for chronic heart failure: a meta-analysis focusing on the number needed to treat. Front Cardiovasc Med. 2023;10(no pagination):1236008. <https://doi.org/10.3389/fcvm.2023.1236008>.

8. Thomopoulos C, Parati G, Zanchetti A. Effects of blood pressure-lowering treatment on cardiovascular outcomes and mortality: 14 - effects of different classes of antihypertensive drugs in older and younger patients: overview and meta-analysis. J Hypertens. 2018;36(8):1637-47. <http://dx.doi.org/10.1097/hjh.0000000000001777>.

9. Zhong XL, Dong Y, Xu W, Huang YY, Wang HF, Zhang TS, et al. Role of Blood Pressure Management in Stroke Prevention: A Systematic Review and Network Meta-Analysis of 93 Randomized Controlled Trials. J. 2021;23(1):1-11. <http://dx.doi.org/10.5853/jos.2020.02698>.

10. Sakima A, Arima H, Matayoshi T, Ishida A, Ohya Y. Effect of Mineralocorticoid Receptor Blockade on Arterial Stiffness and Endothelial Function: A Meta-Analysis of Randomized Trials. Hypertension. 2021;77(3):929-37. <http://dx.doi.org/10.1161/hypertensionaha.120.16397>.

11. Li S, Zhang X, Dong M, Gong S, Shang Z, Jia X, et al. Effects of spironolactone in heart failure with preserved ejection fraction: A meta-analysis of randomized controlled trials. Medicine (Baltimore). 2018;97(35):e11942. <http://dx.doi.org/10.1097/md.0000000000011942>.

12. Yi X, Yang S, Yang J, Chen X, Zhang A, Zeng Q, et al. Renin-Angiotensin-Aldosterone System Modulators in Adults with Hypertension: A Network Meta-Analysis of Randomized Controlled Trials. Drugs. 2024;23:23. <https://doi.org/10.1007/s40265-024-02092-7>.

13. Desbiens LC, Khelifi N, Wang YP, Lavigne F, Beaulieu V, Sidibe A, et al. Thiazide Diuretics and Fracture Risk: A Systematic Review and Meta-Analysis of Randomized Clinical Trials. JBMR Plus. 2022;6(11):e10683. <http://dx.doi.org/10.1002/jbm4.10683>.
