# Supplementary material for: Effectiveness and safety of chronic diuretic use in older adults: an umbrella review of recently published systematic reviews and meta-analyses of randomized-controlled trials
Source: Eur Geriatr Med. 2025 May 25;16(4):1353–87. doi: 10.1007/s41999-025-01229-5 (PMC12378697; doi:10.1007/s41999-025-01229-5)
Supplement: Supplementary file 5 — Supplementary file5 (DOCX 70 KB) [file 41999_2025_1229_MOESM5_ESM.docx]

**Table 4b.** Summary of findings table, narrative summary non-pooled data comparing different diuretics or diuretic (sub)classes.

| **Review first author, year of publication** | **Outcome category** | **Specific outcome** | **Diuretic indication, population** | **Diuretic A** | **Diuretic B** | **Effect**  **(metric, 95% CI)** | **Narrative summary** |
| --- | --- | --- | --- | --- | --- | --- | --- |
| Yang et al. 2019 | biochemistry | hyperkalemia | CKD and T2D | finerenone | eplerenone | OR 0.77  (0.45 to 1.32) | In patients with CKD and T2D, the risk of **hyperkalemia** was comparable with finerenone and spironolactone (n=1), and lower for finerenone compared to spironolactone (n=1). In patients with primary hyperaldosteronism, the odds of **hyperkalemia** were lower with eplerenone compared to spironolactone (n=1). |
|  |  |  |  |  | spironolactone | OR 0.23  (0.13 to 0.39) |  |
| Ho et al. 2024 |  |  | primary hyper aldosteronism | eplerenone |  | OR 0.51  (0.05 to 4.98) |  |
| Täger et al. 2019 | biochemistry | hypokalemia | HFrEF | azosemide | furosemide | RR 3.04  (0.6 to 15.3) | In a network meta-analysis of RCTs, the risk of developing **hypokalemia** associated with diuretic use in patients with chronic systolic HF was studied (Täger 2019). The risk was comparable between different loop diuretics. |
|  |  |  |  | bumetanide | azosemide | RR 0.05  (0.00 to 1.51) |  |
|  |  |  |  |  | furosemide | RR 0.15  (0.01 to 3.06) |  |
|  |  |  |  | spironolactone | azosemide | RR 0.33  (0.00 to 24.11) |  |
|  |  |  |  |  | bumetanide | RR 6.59  (0.05 to 962.82) |  |
|  |  |  |  |  | furosemide | RR 1.00  (0.02 to 53.46) |  |
|  |  |  |  | thiazide | azosemide | RR 0.52  (0.07 to 3.62) |  |
|  |  |  |  |  | bumetanide | RR 10.37  (0.54 to 199.75) |  |
|  |  |  |  |  | furosemide | RR 1.57  (0.53 to 4.64) |  |
|  |  |  |  |  | spironolactone | RR 1.57  (0.03 to 97.19) |  |
|  |  |  |  | torasemide | azosemide | RR 1.00  (0.03 to 36.57) |  |
|  |  |  |  |  | bumetanide | RR 20.02  (0.25 to 1630.58) |  |
|  |  |  |  |  | furosemide | RR 3.04  (0.12 to 75.77) |  |
|  |  |  |  |  | spironolactone | RR 3.04  (0.02 to 506.62) |  |
|  |  |  |  |  | thiazide | RR 1.93  (0.06 to 57.46) |  |
| Alexandrou et al. 2019 | biochemistry | potassium | proteinuric kidney disease | MRA | other diuretic | MD 0.36  (0.16 to 0.55) | In patients with proteinuric kidney disease, MRAs increased **serum potassium** compared to other diuretics (n=1). In patients with HF, spironolactone increased **serum potassium** compared to the loop diuretics azosemide (n=1), bumetanide (n=1), etozolin (n=1) and furosemide (n=1), and compared to the thiazide bendroflumethiazide (n=1). In patients with hypertension (n=1) and with CHD (n=1), chlorthalidone reduced **serum potassium** compared to hydrochlorothiazide. In patients with primary hyperaldosteronism eplerenone (n=1) and esaxerenone (n=1) reduced **serum potassium** compared to spironolactone. |
| Hu et al. 2022 |  |  | DKD | eplerenone | hydrochloro thiazide or spironolactone | MD 0.19  (0.14 to 0.24) |  |
| Eid et al. 2021 |  |  | HF | spironolactone | azosemide | MD 0.62  (0.28 to 0.96) |  |
|  |  |  |  |  | bendroflume thiazide | MD 0.60  (0.20 to 1.00) |  |
|  |  |  |  |  | bumetanide | MD 0.61  (0.25 to 0.97) |  |
|  |  |  |  |  | etozolin | MD 0.652  (0.12 to 0.92) |  |
|  |  |  |  |  | furosemide | MD 0.30  (0.05 to 0.55) |  |
| Dineva et al. 2020 |  |  | HT | chlorthalidone | hydrochloro thiazide | WMD -0.23  (-0.27 to -0.19) |  |
| Dineva et al. 2019 |  |  | HT or coronary heart disease |  |  | MD -0.22  (-0.32 to -0.11) |  |
| Ho et al. 2024 |  |  | primary hyper aldosteronism | eplerenone | spironolactone | MD -0.20  (-0.37 to -0.03) |  |
|  |  |  |  | esaxerenone |  | MD -0.30  (-0.83 to 0.23) |  |
| Eid et al. 2021 | biochemistry | sodium | HF | bumetanide | furosemide | MD 0.30  (0.05 to 0.5) | In patients with HF, bumetanide increased **serum sodium** compared to furosemide (n=1). |
| Teixeira et al. 2024 | cardio vascular | body weight | HF | torasemide | furosemide | MD 2.81  (-1.70 to 7.32) | In patients with HF, the effects on **body weight** were comparable for torasemide and furosemide (n=1). |
| Eid et al. 2021 | cardio vascular | collagen volume fraction | HF | torasemide | furosemide | SMD -0.206  (-0.867 to -3.253) | In patients with HF, torasemide reduced **collagen volume fraction** compared to furosemide (n=1). |
| Elshahat et al. 2024 | cardio vascular | composite | HF | eplerenone | spironolactone | HR 0.95  (0.72 to 1.25) | In patients with HF, the risk of developing a **composite CV outcome** was comparable between eplerenone and spironolactone (n=1). |
| Macfarlane et al. 2019 | cardio vascular | coronary events | HT, primary | indapamide | bendroflume thiazide | RR 0.73  (0.3 to 1.79) | In patients with primary HT, the risk of **coronary events** (n=1) or **CV events** (n=1) was comparable between indapamide and bendroflumethiazide. |
|  |  | CV events |  |  |  | RR 0.89  (0.67 to 1.18) |  |
| Hu et al. 2022 | cardio vascular | DBP | DKD | eplerenone | hydrochloro thiazide or spironolactone | MD -0.71  (-2.00 to 0.59) | In patients with DKD, the effect on DBP was comparable for eplerenone comparared to either hydrochlorothiazide or spironolactone (n=1). In patients with HT or CHD (n=1) but not in patients with HT (n=1), chlorthalidone reduced **DBP** compared to hydrochlorothiazide. In patients with primary HT, the effects on **DBP** were comparable with indapamide and bendroflumethiazide (n=1). In patients with HF, the effect on **DBP** was comparable between torasemide and furosemide (n=2). In patients with primary hyperaldosteronism, eplerenone (n=1), but not esaxerenone (n=1) reduced **DBP** compared to spironolactone. |
| Sherif et al. 2019 |  |  | HF | torasemide | furosemide | SMD 0.331  (-0.203 to 0.866) |  |
| Dineva et al. 2019 |  |  | HT or CHD | chlorthalidone | hydrochloro thiazide | WMD -2.41  (-3.87 to -0.95) |  |
| Dineva et al. 2020 |  |  | HT |  |  | WMD -0.67  (-1.92 to 0.57) |  |
| Macfarlane et al. 2019 |  |  | HT, primary | indapamide | bendroflume thiazide | MD -0.4  (-0.93 to 0.14) |  |
| Teixeira et al. 2024 |  |  | HF | torasemide | furosemide | MD 0.18  (-2.48 to 2.84) |  |
| Ho et al. 2024 |  |  | primary hyper aldosteronism | eplerenone | spironolactone | MD -4.63  (-8.87 to -0.40) |  |
|  |  |  | primary hyper aldosteronism | esaxerenone |  | MD -0.90  (-7.43 to 9.23) |  |
| Eid et al. 2021 | cardio vascular | diuresis and water excretion | HF | amiloride | furosemide | MD -355.16  (-1015.06 to 304.74) | A systematic review and meta-analysis of RCTs in patients with HF (Eid 2021) found no difference in **diuresis and water excretion** between furosemide and the loop diuretics azosemide (n=1), bumetanide (n=1) and torasemide (n=1), the potassium sparing diuretic amiloride (n=1) or the thiazide hydrochlorothiazide (n=1). |
|  |  |  |  | azosemide |  | MD 25  (-561.02 to 611.02) |  |
|  |  |  |  | bumetanide |  | MD 76.79  (-340.97 to 494.55) |  |
|  |  |  |  | hydrochloro thiazide |  | MD -442.00  (-1380.09 to 496.09) |  |
|  |  |  |  | torasemide |  | MD 314.59  (-51.18 to 680.36) |  |
| Sherif et al. 2019 | cardio vascular | heart rate | HF | torasemide | furosemide | SMD -0.093  (-0.349 to 0.162) | In patients with HF, the effect on heart rate was comparable with torasemide and furosemide (n=1). |
| Eid et al. 2021 | cardio vascular | natriuresis and sodium excretion | HF | amiloride | furosemide | MD -23.52  (-75.38 to 28.34) | A systematic review and meta-analysis of RCTs in patients with HF (Eid 2021) found no difference in **natriuresis and sodium excretion** between furosemide and the loop diuretics azosemide (n=1), bumetanide (n=1) and torasemide (n=1), the potassium sparing diuretic amiloride (n=1); **sodium excretion** increase (n=1), **urinary volume** (n=1) and **weight loss** (n=1) were comparable between torasemide and furosemide. Compared to furosemide, torasemide had comparable effects on **edema** (n=2), or reduced **edema** (n=1). The odds of improved NYHA class was comparable for torasemide and furosemide (n=1). In patients with HF, the odds of NYHA class improvement were higher for torasemide compared to furosemide (n=2), or comparable (n=1). The likeliness of improving 1 or more NYHA classes was comparable between torasemide and furosemide (n=1). |
|  |  |  |  | azosemide |  | MD -6.00  (-32.91 to 20.91) |  |
|  |  |  |  | bumetanide |  | MD 12.60  (-4.29 to 29.49) |  |
|  |  |  |  | torasemide |  | MD -53  (-31.95 to 30.90) |  |
| Sherif et al. 2019 |  | sodium excretion increase |  |  |  | SMD 0.18  (-0.33 to 0.7) |  |
|  |  | urinary volume |  |  |  | SMD -0.25  (-0.5 to 0.05) |  |
|  |  | weight loss |  |  |  | SMD 0.03  (-0.12 to 0.18) |  |
|  |  | edema improvement |  |  |  | OR 0.74  (0.35 to 1.54) |  |
| Eid et al. 2021 |  |  |  |  |  | RR 1.54  (1.01 to 2.35) |  |
| Sherif et al. 2019 |  | edema worsening |  |  |  | OR 0.81  (0.24 to 2.79) |  |
| Kido et al. 2019 |  | NYHA class improvement |  |  |  | OR 1.44  (1.18 to 1.76) |  |
| Miles et al. 2019 |  |  |  |  |  | OR 0.91  (0.61 to 1.35) |  |
| Abraham et al. 2020 |  |  |  |  |  | OR 3.31  (1.57 to 6.99) |  |
| Teixeira et al. 2024 |  | NYHA, improvement of ≥ 1 NYHA class |  |  |  | RR 1.25  (0.92 to 1.68) |  |
| Yang et al. 2022 | cardio vascular | SBP | CKD and T2D | MRA, nonsteroidal | MRA, steroidal | WMD -10.37  (-20.19 to -0.54) | In patients with CKD and T2D, **SBP** was lower with nonsteroidal compared to steroidal MRAs (n=1). In patients with DKD, **SBP** was higher in eplerenone compared to hydrochlorothiazide or spironolactone (n=1). In patients with HT or CHD chlorthalidone reduced **SBP** compared to hydrochlorothiazide (n=1). In patients with (primary) HT, the effects on **SBP** were comparable with indapamide and bendroflumethiazide (n=1) and with chlorthalidone and hydrochlorothiazide (n=1). In patients with HF, the effects on **SBP** was comparable between torasemide and furosemide (n=2). In patients with primary hyperaldosteronism, the effects on **SBP** were comparable between eplerenone (n=1) or esaxerenone (n=1) compared to spironolactone. |
| Hu et al. 2022 |  |  | DKD | eplerenone | hydrochlorothiazide or spironolactone | MD 3.7  (2.43 to 4.97) |  |
| Sherif et al. 2019 |  |  | HF | torasemide | furosemide | SMD 0.163  (-0.27 to 0.618) |  |
| Teixeira et al. 2024 |  |  |  |  |  | MD 2.68  (-0.32 to 5.68) |  |
| Dineva et al. 2020 |  |  | HT | chlorthalidone | hydrochlorothiazide | WMD -2.35  (-5.52 to 0.83) |  |
| Dineva et al. 2019 |  |  | HT or CHD |  |  | WMD -3.26  (-4.58 to -1.94) |  |
| Macfarlane et al. 2019 |  |  | HT, primary | indapamide | bendroflumethiazide | MD -0.26  (-0.79 to 0.27) |  |
| Ho et al. 2024 |  |  | primary hyper aldosteronism | eplerenone | spironolactone | MD -10.07  (-22.76 to 2.62) |  |
|  |  |  |  | esaxerenone |  | MD -2.90  (-22.73 to 16.93) |  |
| Macfarlane et al. 2019 | cardio vascular | stroke | HT, primary | indapamide | bendroflumethiazide | RR 2.21  (1.19 to 4.11) | In patients with primary HT, **stroke** risk was higher with indapamide compared to bendroflumethiazide (n=1). |
| Eid et al. 2021 | heart biomarkers | BNP | HF | azosemide | furosemide | MD -33.87  (-46.33 to -21.40) | In patients with HF, **serum BNP** was higher with furosemide compared to azosemide (n=2) and to torasemide (n=2); the effect on **BNP** was comparable for furosemide compared to spironolactone (n=1). **BNP/nt proBNP ratio** was higher in furosemide compared to torasemide (n=1). In patients with HF, the odds of a >30% reduction in NTproBNP were similar between finerenone and eplerenone (n=1). Similarly, in patients with CKD this risk was comparable (n=1). |
| Zhao et al. 2019 |  |  |  | furosemide | azosemide | MD 18.00  (8.44 to 27.56) |  |
|  |  |  |  | torasemide | furosemide | MD -57.55  (-92.7 to -22.4) |  |
| Eid et al. 2021 |  |  |  | spironolactone |  | MD -7.00  (-73.75 to 59.75) |  |
|  |  |  |  | torasemide |  | MD -64.00  (-122.57 to -5.43) |  |
| Singh et al. 2023 |  | BNP/NT proBNP |  |  |  | SMD -0.34  (-0.76 to 0.07) |  |
| Teixeira et al. 2024 |  | NT-proBNP |  |  |  | MD -226.86  (-443.69 to -10.02) |  |
| Chen et al. 2024 |  | NT-proBNP, 30% reduction | CKD | finerenone | eplerenone | RR 1.03  (0.84 to 1.25) |  |
| Yang et al. 2019 |  |  | HF |  |  | OR 1.33  (0.81 to 2.17) |  |
| Teixeira et al. 2024 | heart ultrasound | LVEDV | HF | torasemide | furosemide | MD -16.06  (-34.32 to 2.21) | In patients with HF, **LVEDV** was lower with torasemide compared to furosemide (n=1); LVMI was comparable between both treatments (n=1). |
|  |  | LVMI |  |  |  | MD -4.70  (-10.18 to 0.79) |  |
| Zhao et al. 2019 | heart ultrasound | LVEF | HF | furosemide | azosemide | MD -0.6  (-0.87 to -0.32) | In patients with HF, effects on **LVEF** for torasemide compared to furosemide were inconsistent (n=2 higher LVEF with torasemide, n=1 comparable effect); LVEF was lower with furosemide compared to azosemide (n=1). |
|  |  |  |  |  | furosemide | MD 0.59  (1.15 to 2.34) |  |
| Sherif et al. 2019 |  |  |  |  |  | SMD -0.15  (-0.46 to 0.16) |  |
| Teixeira et al. 2024 |  |  |  |  |  | MD 4.51  (2.94 to 6.07) |  |
| Singh et al. 2023 | Hospitali zation | all cause | HF | torasemide | furosemide | RR 0.94  (0.90 to 0.99) | In patients with HF, the odds for and risk of **all cause hospitalization** (n=1 and n=2, respectively) was lower with torasemide compared to furosemide. |
| Teixeira et al. 2024 |  |  |  |  |  | RR 0.72  (0.60 to 0.88) |  |
| Siddiqi et al. 2023 |  |  |  |  |  | OR 0.84  (0.73 to 0.98) |  |
| Täger et al. 2019 | hospi  talization | HF-related | HFrEF | azosemide | furosemide | RR 0.58  (0.32 to 1.04) | In patients with HFrEF, the risk of **HF related hospitalization** was comparable between azosemide and furosemide (n=1) and between azosemide and torasemide (n=1). |
|  |  |  |  | torasemide | azosemide | RR 0.7  (0.35 to 1.4) |  |
| Yang et al. 2019 | hospi talization | CV | HF | finerenone | eplerenone | OR 0.05  (0.01 to 0.2) | In patients with HF, the risk of **CV related hospitalization** was lower with torasemide compared to furosemide (n=2), and lower with finerenone compared to eplerenone (n=1). The risk of **HF related re-hospitalization** was higher with torasemide compared to furosemide (n=1), while the risk for **CV-related re-hospitalization** was comparable between torasemide and furosemide (n=2). Hospital stay length was shorter with torasemide compared to furosemide (n=1). |
| Singh et al. 2023 |  |  |  | torasemide | furosemide | RR 0.74  (0.61 to 0.88) |  |
| Teixeira et al. 2024 |  |  |  |  |  | RR 0.72  (0.60 to 0.88) |  |
| Sherif et al. 2019 |  | hospital stay length |  |  |  | SMD -0.379  (-0.562 to -0.197) |  |
| Kido et al. 2019 |  | readmissions, CV-related |  |  |  | OR 0.83  (0.62 to 1.12) |  |
| Shah et al. 2018 |  |  |  |  |  | OR 1.89  (1.15 to 3.13) |  |
|  |  | readmissions, HF-related |  |  |  | OR 3.03  (2.00 to 4.55) |  |
| Yang et al. 2019 | kidney | composite | CKD and T2D | MRA, nonsteroidal | MRA, steroidal | OR 0.48  (0.15 to 1.41) | In patients with CKD and T2D, the odds for a **composite kidney outcome** was comparable for nonsteroidal compared to steroidal MRAs (n=1). |
| Hu et al. 2022 | kidney | eGFR | DKD | eplerenone | hydrochloro thiazide | MD -3.00  (-26.7 to 20.7) | In patients with DKD, the effect on **eGFR** was comparable with eplerenone compared to hydrochlorothiazide (n=1); in patients with HF, the effect on **eGFR** was comparable with furosemide compared to azosemide (n=1); in patients with proteinuric kidney disease the effect op **eGFR** was comparable with MRAs compared to other diuretics (n=1). |
| Eid et al. 2021 |  |  | HF | furosemide | azosemide | MD 0.113  (-0.081 to 0.307) |  |
| Alexandrou et al. 2019 |  |  | proteinuric kidney disease | MRA | other diuretic | MD 0.88  (-2.42 to 4.17) |  |
| Yang et al. 2022 | kidney | eGFR, >57% reduction | CKD and T2D | MRA, nonsteroidal | MRA, steroidal | OR 0.44  (0.14 to 1.31) | In patients with CKD and T2D, the risk of a **>57% eGFR reduction** was comparable between nonsteroidal and steroidal MRAs (n=1). |
| Hu et al. 2022 | kidney | fibrosis (laminin) | DKD | eplerenone | spironolactone | MD -8.22  (-11.48 to -4.95) | In patients with DKD, **kidney fibrosis (laminin)** was lower with eplerenone compared to spironolactone (n=1). |
| Yang et al. 2022 | kidney | kidney failure | CKD and T2D | MRA, nonsteroidal | MRA, steroidal | OR 0.69  (0.03 to 16.83) | In patients with CKD and T2D, the odds for **kidney failure** were comparable with nonsteroidal compared to steroidal MRAs (n=1). |
| Frankenstein et al. 2020 | kidney | AKI | HF, chronic | eplerenone | canrenone | HR 0.23  (0.00 to 21.24) | In a network meta-analysis of RCTs, the risk of **AKI** associated with diuretic use in patients with chronic systolic HF was studied (n=6; Täger 2019). The risk was comparable between different diuretics; in a systematic review and meta-analysis of RCTs in patients with chronic HF, the risk of **AKI** was comparable between different MRAs (n=3; Frankenstein 2020). |
|  |  |  |  | spironolactone | canrenone | HR 0.51  (0.01 to 43.53) |  |
|  |  |  |  |  | eplerenone | HR 2.17  (0.01 to 404.15) |  |
| Täger et al. 2019 |  |  | HFrEF | spironolactone | furosemide | RR 1.00  (0.02 to 53.46) |  |
|  |  |  |  | thiazide |  | RR 0.59  (0.05 to 7.29) |  |
|  |  |  |  |  | spironolactone | RR 0.59  (0.01 to 65.18) |  |
|  |  |  |  | torasemide | furosemide | RR 0.29  (0.08 to 1.03) |  |
|  |  |  |  |  | spironolactone | RR 0.29  (0.00 to 18.67) |  |
|  |  |  |  |  | thiazide | RR 0.49  (0.03 to 8.2) |  |
| Hu et al. 2022 | kidney | microalbuminuria | DKD | eplerenone | spironolactone | MD -7.58  (-9.62 to -5.55) | in patients with DKD, **microalbuminuria** was reduced with eplerenone compared to spironolactone (n=1). |
| Sherif et al. 2019 | kidney | potassium excretion | HF | torasemide | furosemide | SMD 0.64  (0.1 to 1.17) | In patients with HF, **potassium excretion** was comparable with torasemide and with furosemide (n=1). |
| Hu et al. 2022 | kidney | proteinuria, 24hr | DKD | eplerenone | hydrochloro thiazide or spironolactone | MD -19.39  (-23.52 to -15.27) | In patients with DKD, **24hr proteinuria** was comparable for eplerenone compared to hydrochlorothiazide or spironolactone (n=1). |
| Alexandrou et al. 2019 | kidney | UACR | proteinuric kidney disease | MRA | other diuretic | MD -6.2  (-43.51 to 31.1) | In patients with proteinuric kidney disease, the effect on **UACR** was comparable between MRAs compared to other diuretics (n=1). |
| Yang et al. 2022 | mortality | all cause | CKD and T2D | MRA, nonsteroidal | MRA, steroidal | OR 1.32  (0.19 to 11.32) | In patients with CKD and T2D, **all cause mortality** was comparable between nonsteroidal and steroidal MRAs (n=1); in . |
| Zhao et al. 2019 | mortality | all cause | HF | furosemide | azosemide | OR 0.98  (0.48 to 2.02) | In patients with HF, the odds for **all cause mortality** were comparable for furosemide compared to azosemide (n=1) and for torasemide compared to furosemide (n=1). |
| Shah et al. 2018 |  |  |  | torasemide | furosemide | OR 1.22  (0.78 to 1.92) |  |
| Singh et al. 2023 | mortality | all cause | HF | torasemide | furosemide | RR 0.98  (0.87 to 1.10) | In a network meta-analysis of RCTs, the risk of **all cause mortality** associated with diuretic use in patients with chronic systolic HF was studied (n=15; Täger 2019). The risk was comparable between different diuretics; in patients with HF, **all cause mortality** was comparable between torasemide and furosemide (n=1); in patients with primary HT, the risk of **all cause mortalit**y was comparable between indapamide and bendroflumethiazide (n=1). |
| Täger et al. 2019 |  |  | HFrEF | azosemide |  | RR 0.99  (0.5 to 8.2) |  |
|  |  |  |  | bumetanide | azosemide | RR 2.97  (0.59 to 15.05) |  |
|  |  |  |  |  | furosemide | RR 2.93  (0.67 to 12.82) |  |
|  |  |  |  | spironolactone | azosemide | RR 1.02  (0.02 to 57.4) |  |
|  |  |  |  |  | bumetanide | RR 0.34  (0.00 to 23.81) |  |
|  |  |  |  |  | furosemide | RR 1.00  (0.02 to 53.46) |  |
|  |  |  |  | thiazide | azosemide | RR 1.06  (0.25 to 4.29) |  |
|  |  |  |  |  | bumetanide | RR 0.36  (0.06 to 3.56) |  |
|  |  |  |  |  | furosemide | RR 1.04  (0.3 to 3.56) |  |
|  |  |  |  |  | spironolactone | RR 1.04  (0.02 to 67.05) |  |
|  |  |  |  | torasemide | azosemide | RR 0.82  (0.38 to 1.79) |  |
|  |  |  |  |  | bumetanide | RR 0.28  (0.06 to 1.28) |  |
|  |  |  |  |  | furosemide | RR 0.81  (0.55 to 1.2) |  |
|  |  |  |  |  | spironolactone | RR 0.81  (0.01 to 44.23) |  |
|  |  |  |  |  | thiazide | RR 0.78  (0.21 to 2.83) |  |
| Macfarlane et al. 2019 |  |  | HT, primary | indapamide | bendroflumethiazide | RR 0.82  (0.57 to 1.18) |  |
| Yang et al. 2019 | mortality | CV | CKD and T2D | finerenone | eplerenone | OR 0.02  (0.00 to 0.39) | In patients with CKD and T2D, the odds for **CV mortality** were lower for finerenone compared to eplerenone (n=1) and compared to spironolactone (n=1); in patients with HF, the odds for **CV mortality** were comparable for torasemide compared to furosemide (n=1); in a network meta-analysis of RCTs, the risk of **CV mortality** associated with diuretic use in patients with chronic systolic HF was studied (Täger 2019). The risk was comparable between different loop diuretics; in patients with primary HT, the risk of **CV mortality** was comparable for indapamide compared to bendroflumethiazide (n=1). |
|  |  |  |  |  | spironolactone | OR 0.01  (0.00 to 0.14) |  |
| Abraham et al. 2020 |  |  | HF | torasemide | furosemide | OR 0.55  (0.2 to 1.54) |  |
| Täger et al. 2019 |  |  | HFrEF | azosemide | furosemide | RR 0.65  (0.27 to 1.61) |  |
|  |  |  |  | bumetanide | azosemide | RR 5.04  (0.43 to 89.23) |  |
|  |  |  |  |  | furosemide | RR 3.3  (0.33 to 32.74) |  |
|  |  |  |  | spironolactone | azosemide | RR 1.53  (0.03 to 90.23) |  |
|  |  |  |  |  | bumetanide | RR 0.3  (0.00 to 29.91) |  |
|  |  |  |  |  | furosemide | RR 1.00  (0.02 to 53.46) |  |
|  |  |  |  | thiazide | azosemide | RR 1.6  (0.32 to 8.26) |  |
|  |  |  |  |  | bumetanide | RR 0.32  (0.03 to 3.92) |  |
|  |  |  |  |  | furosemide | RR 1.05  (0.26 to 70.46) |  |
|  |  |  |  |  | spironolactone | RR 1.05  (0.02 to 70.46) |  |
|  |  |  |  | torasemide | azosemide | RR 0.99  (0.23 to 3.47) |  |
|  |  |  |  |  | bumetanide | RR 0.2  (0.02 to 2.29) |  |
|  |  |  |  |  | furosemide | RR 0.65  (0.27 to 1.56) |  |
|  |  |  |  |  | spironolactone | RR 0.65  (0.01 to 38.1) |  |
|  |  |  |  |  | thiazide | RR 0.62  (0.12 to 3.16) |  |
| Macfarlane et al. 2019 |  |  | HT, primary | indapamide | bendroflumethiazide | RR 0.82  (0.55 to 1.2) |  |
| Macfarlane et al. 2019 | mortality | non-CV | HT, primary | indapamide | bendroflumethiazide | RR 0.81  (0.54 to 1.22) | In patients with primary HT, the risk of non-CV mortality was comparable for indapamide compared to bendroflumethiazide (n=1). |
| Zhao et al. 2019 | various AEs | any AE | HF | furosemide | azosemide | OR 0.69  (0.21 to 2.24) | In patients with HF, the odds of **adverse events** was comparable between furosemide and azosemide (n=1) and lower for finerenone compared to spironolactone (n=1). In patients with a history of MI, the odds of adverse events was lower for finerenone compared to spironolactone (n=1). In patients with HF, the odds of **discontinuation** was comparable between eplerenone and spironolactone (n=1) . The risk of **drug-related AEs** was comparable between torasemide and furosemide (n=1) The risk of **hyperkalemia-related AEs** was higher with MRAs compared to other diuretics in patients with HT (n=1). In patients with HF, the risk of **gynecomastia** was higher for spironolactone compared to eplerenone (n=2). Also in patients with primary hyperaldosteronism, the risk of **gynecomastia** was higher with spironolactone compared to eplerenone (n=1). |
|  |  |  |  |  | torasemide | OR 0.66  (0.47 to 0.93) |  |
| Xu et al. 2018 |  |  | post MI | finerenone | spironolactone | OR 0.21  (0.16 to 0.28) |  |
| Bazoukis et al. 2018_1 |  | hyperkalemia-related | HT | MRA | other diuretic | RR 3.73  (1.72 to 8.09) |  |
| Elshahat et al. 2024 |  | discontinuation | HF | eplerenone | spironolactone | RR 0.75  (0.21 to 2.65) |  |
| Abraham et al. 2020 |  | drug-related |  | torasemide | furosemide | OR 1.27  (0.65 to 2.48) |  |
| Elshahat et al. 2024 |  | gynecomastia |  | eplerenone | spironolactone | RR 0.07  (0.02 to 0.31) |  |
| Frankenstein et al. 2020 |  |  | HF, chronic |  |  | RR 0.09  (0.02 to 0.48) |  |
| Ho et al. 2024 |  |  | primary hyper aldosteronism |  |  | RR 4.69  (3.58 to 6.14) |  |

AE: adverse event; AKI: acute kidney injury; BNP: brain natriuretic peptide; CHD: coronary heart disease; CI: confidence interval; CKD: chronic kidney disease; CV: cardiovascular; DBP: diastolic blood pressure; DKD: diabetic kidney disease; eGFR: estimated glomerular filtration rate; HF: heart failure; HFrEF: heart failure with reduced ejection fraction; HR: hazard ratio; HT: hypertension; LVEDV: left ventricular end diastolic volume; LVEF: left ventricular ejection fraction; LVMI: left ventricular mass index; MD: mean difference; MI: myocardial infarction; MRA: mineralocorticoid receptor antagonist; NT-proBNP: N-terminal pro b-type natriuretic peptide; NYHA: New York Heart Association; OR: odds ratio; RCT: randomized controlled trial; RR: risk ratio; SBP: systolic blood pressure; SMD: standardized mean difference; T2D: type 2 diabetes mellitus; UACR: urinary albumin to creatinine ratio; WMD: weighted mean difference.
